# Discovery of 3-Arylquinoxaline Derivatives as Potential Anti-Dengue Virus Agents

**DOI:** 10.3390/ijms20194786

**Published:** 2019-09-26

**Authors:** Chih-Hua Tseng, Cheng-Ruei Han, Kai-Wei Tang

**Affiliations:** 1 School of Pharmacy, College of Pharmacy, Kaohsiung Medical University, Kaohsiung 807, Taiwan; st88036942017@gmail.com (C.-R.H.); dadaking1107@gmail.com (K.-W.T.); 2 Department of Fragrance and Cosmetic Science, College of Pharmacy, Kaohsiung Medical University, Kaohsiung 807, Taiwan; 3 Drug Development and Value Creation Research Center, Kaohsiung Medical University, Kaohsiung 807, Taiwan; 4 Department of Medical Research, Kaohsiung Medical University Hospital, Kaohsiung 807, Taiwan; 5 Department of Pharmacy, Kaohsiung Municipal Ta-Tung Hospital, Kaohsiung 801, Taiwan

**Keywords:** 3-arylquinoxaline, dengue virus, ribavirin, cyclooxygenase-2

## Abstract

We designed and synthesized a series of novel 3-arylquinoxaline derivatives and evaluated their biological activities as potential dengue virus (DENV) replication inhibitors. Among them, [3-(4-methoxyphenyl)quinoxalin-2-yl](phenyl)methanol (**19a**), [6,7-dichloro-3-(4-methoxyphenyl)quinoxalin-2-yl](phenyl)methanol (**20a**), and (4-methoxyphenyl)(3-phenylquinoxalin-2-yl)methanone (**21b**) were found to significantly inhibit the DENV RNA expression in Huh-7-DV-Fluc cells with a potency better than that of ribavirin. Compound **19a** reduced DENV replication in both viral protein and messenger RNA (mRNA) levels in a dose-dependent manner and exhibited no significant cell cytotoxicity. Notably, compound **19a** exhibited a half maximal effective concentration (EC_50_) value at 1.29 ± 0.74 μM. We further observed that the inhibitory effect of **19a** on DENV replication was due to suppression of DENV-induced cyclooxygenase-2 (COX-2) expression. Docking studies also showed that **19a** caused hydrophobic interactions at the active sites with Arg29, Glu31, Tyr116, Leu138, Pro139, Lys454, Arg455, and Gln529. The calculated lowest binding energy between the **19a** and COX-2 was −9.10 kcal/mol. In conclusion, compound **19a** might be a potential lead compound for developing an anti-DENV agent.

## 1. Introduction

Dengue virus (DENV) infections significantly increased in the past decades and, with more than half the world’s population living in areas at risk of infection, the World Health Organization estimated the true number of cases to exceed 50 million annually [1]. There are four serotypes of dengue, DENV-1, DENV-2, DENV-3, and DENV-4, and the infection severity ranges from the self-limiting dengue fever (DF) to the more serious dengue hemorrhagic fever (DHF) and dengue shock syndrome (DSS), with reported ranges of 1–5% to 10–30% of cases resulting in death, respectively [2,3,4]. There is no antiviral drug currently available for the treatment of dengue, although potential anti-DENV drugs being tested in human clinical trials are at different stages of development. Some drugs entered into such trials include chloroquine, celgosivir, balapiravir, prednisolone, lovastatin, ivermectin, and ribavirin; however, none of these are yet approved for treatment against the virus [5]. Therefore, the development of supplemental agents or more effective and safer agents is required for DV therapy.

Cyclooxygenase-2 (COX-2) is one of the important mediators of inflammation in response to viral infection, and it contributes to viral replication, for example, cytomegalovirus or hepatitis C virus replication [6,7,8,9,10]. More recently, Lin et al. revealed that COX-2 is an important factor for DENV replication and can serve as a potential target for developing therapeutic agents against DENV infection [11].

**Figure 1:**
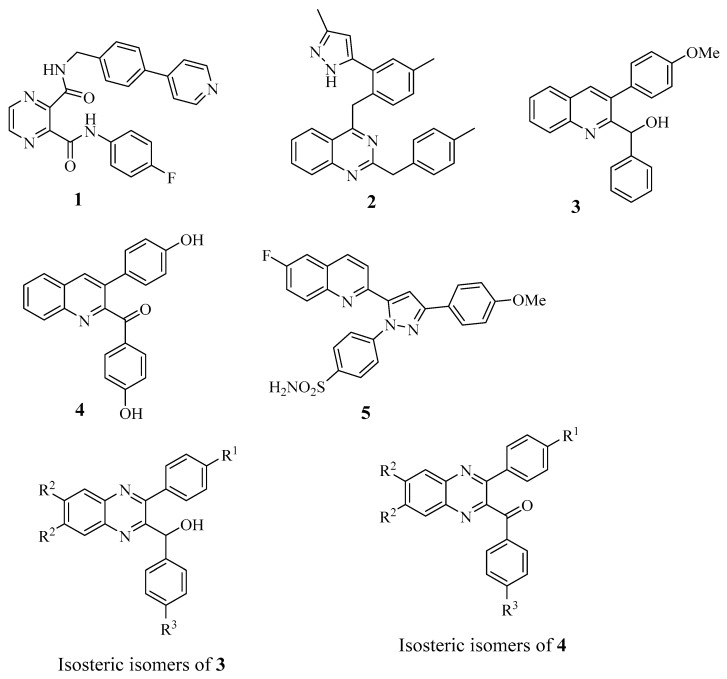
Structures of *N*^2^-(4-fluorophenyl)-*N*^3^-[4-(pyridin-4-yl)benzyl]pyrazine-2,3-dicarboxamide (**1**), 4-[4-methyl-2-(3-methyl-1*H*-pyrazol-5-yl)benzyl)-2-(4-methylbenzyl)quinazoline (**2**), 2-(hydroxyphenyl methyl)-3-(4-methoxyphenyl)quinoline (**3**), 2-(4-hydroxy benzoyl)-3-(4-hydroxyphenyl)quinoline (**4**), 4-[5-(6-fluoroquinolin-2-yl)-3-(4-methoxyphenyl)-1*H*-pyrazol-1-yl]benzenesulfonamide (**5**), and target compounds (**15a**–**26b**; isosteric isomers of compounds **3** and **4**).

Certain heterocyclic compounds were discovered to possess anti-DENV activities. For example, Saudi et al. identified an imidazole 4,5-dicarboxamide derivative as a novel anti-DENV agent through high-throughput screening (HTS) and proceeded to synthesize a series of 29 compounds, from which pyrazine-based compound **1**, with a significant potency against DENV2 in VERO-B cells (EC_50_ = 0.93 μM), was identified [12]. Venkatesham et al. structurally modified the molecule and discovered quinazoline derivative **2** with an EC_50_ value of 2.6 μM against DENV [13]. We also prepared certain quinoline derivatives for anti-DENV evaluation. Among them, 2-(hydroxyphenylmethyl)-3-(4-methoxyphenyl)quinoline (**3**) and 2-(4-hydroxybenzoyl)-3-(4-hydroxyphenyl)quinoline (**4**) were found to inhibit the DENV2 RNA expression in Huh-7-DV-Fluc cells with a higher potency than that of ribavirin [14]. More recently, we further identified 4-[5-(6-fluoroquinolin-2-yl)-3-(4-methoxy phenyl)-1*H*-pyrazol-1-yl]benzenesulfonamide (**5**), which exhibited approximately 10-fold more active anti-DENV-2 activity (EC_50_ = 0.81 μM) than that of ribavirin (EC_50_ = 12.61 μM) [15]. Quinoxaline is a versatile heterocyclic compound and belongs to the isosteric isomer of quinoline. Several quinoxaline-containing compounds possess a wide variety of biological activities including antitubercular, antibacterial, anti-trypanosomal, anticancer, anti-inflammatory, antimalarial, anti-human immunodeficiency virus (HIV), antidepressant, and anti-hyperglycemic effects [16,17,18,19,20,21,22]. The present report describes the synthesis of 3-arylquinoxaline derivatives (target compounds, Figure 1), whose structures are considered as the 4-aza isosteric isomers of compounds **3** and **4**. Quinoxaline is more hydrophilic than quinoline due to an additional nitrogen atom which bears a pair of non-pair electrons to form hydrogen bonding with water. A reasonable high water solubility is important for in vivo and pre-clinical evaluations. Our aim was to discover potential anti-DENV drug candidates with higher potency and less cytotoxicity than our initial lead compounds **3** and **4**.

## 2. Results and Discussion

### 2.1. Chemistry

**Scheme 1:**
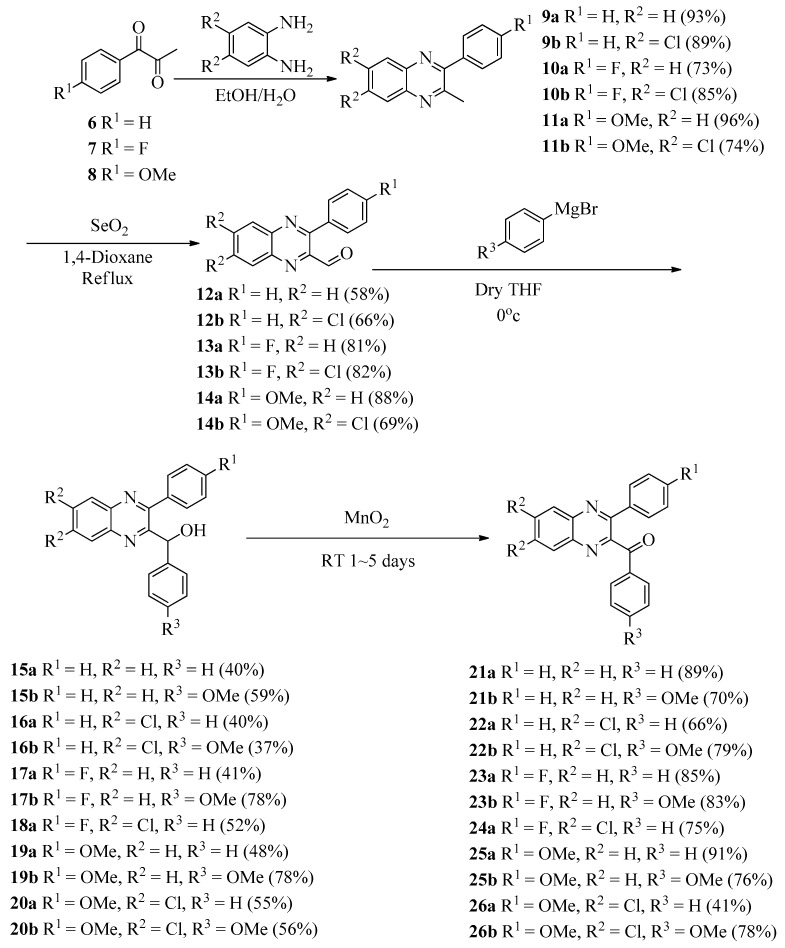
Synthesis of 3-arylquinoxaline derivatives **21a**–**26b.**

**Scheme 2:**
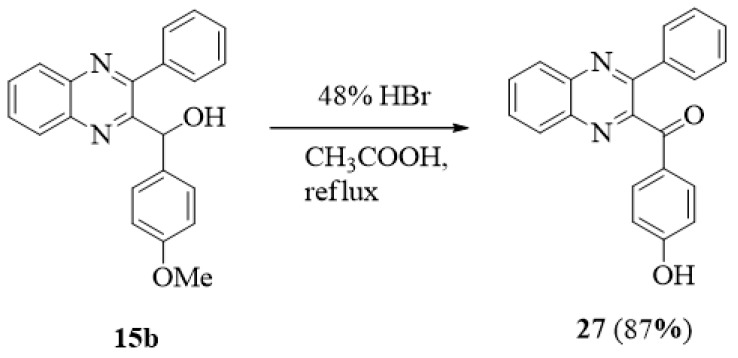
Synthesis of (4-hydroxyphenyl)(3-phenylquinoxalin-2-yl)methanone (**27**).

**Scheme 3:**
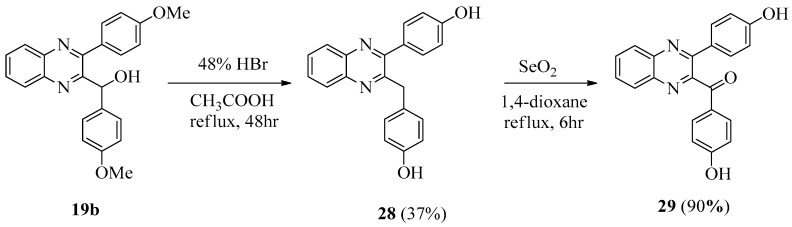
Synthesis of (4-hydroxyphenyl)(3-(4-hydroxyphenyl)quinoxalin-2-yl)methanone (**29**).

The known 2-methyl-3-phenylquinoxaline (**9a**) was prepared via the condensation of 1-phenylpropane-1,2-dione (**6**) and benzene-1,2-diamine according to a previous study [23]. Accordingly, compounds **9b**–**11b** were obtained from the starting materials **6**–**8**, respectively, under the same reaction conditions in a yield of 73–96%. Oxidation of **9a**–**11b** with selenium oxide afforded 3-phenylquinoxaline-2-carbaldehyde (**12a**) or its analogs **12b**–**14b**, respectively, in a yield of 58–88% [24]. Treatment of **12a** with phenyl magnesium bromide (Grignard reagent) afforded phenyl(3-phenylquinoxalin-2-yl)methanol (**15a**), which was then oxidized with MnO_2_ to give phenyl(3-phenylquinoxalin-2-yl)methanone (**21a**) as described in Scheme 1. Accordingly, Grignard reaction of compounds **12b**–**14b** afforded compounds **15b**–**20b**, respectively, which were then oxidized with MnO_2_ to give their respective carbonyl products **21b**–**26b** under the same reaction conditions. Demethylation of **15b** with 48% HBr gave (4-hydroxyphenyl)(3-phenylquinoxalin-2-yl)methanone (**27**) as described in Scheme 2. However, reaction of **19b** with 48% HBr did not give (4-hydroxyphenyl)(3-phenylquinoxalin-2-yl)methanone (**29**) nor the expected 4-[3-(4-hydroxy benzyl)quinoxalin-2-yl]phenol (**28**). Oxidation of intermediate (**28**) with SeO_2_ gave (4-hydroxyphenyl) [3-(4-hydroxyphenyl)quinoxalin-2-yl)methanone (**29**), as described in Scheme 3. The structure of **15a**–**29** was determined by NMR (^1^H and ^13^C) (spectra data can be found in Supplementary Materials) and further confirmed by elemental analysis.

### 2.2. Biological Evaluation

#### 2.2.1. Anti-DENV Activities and Cytotoxicities

The anti-DENV activities and cytotoxicities of 3-arylquinoxaline derivatives are summarized in Table 1. Huh-7-DV-Fluc cells were treated with compounds **3**, **4**, and **15**–**28** for three days. Anti-DENV activities were determined by firefly luciferase activity. Compounds which exhibited >32% inhibition of DENV-2 at a concentration of 10 μM were considered as active [15]. For the 2-hydroxymethyl derivatives, 2-(hydroxyphenyl methyl)-3-phenylquinoline (**15a**) was inactive, while its methoxy derivative (**15b**, R^3^ = OMe) was moderately active and exhibited 48% inhibition of DENV-2 at a concentration of 10 μM. Introduction of MeO group at R^3^ position (**15a** vs. **15b**) strongly enhanced antiviral activity, indicating the electron-donating group at R^3^ position was crucial. The same trend was observed in which **16b** was more active than **16a**, and **17b** was more active than **17a**. Although compound **15b** was less active than the initial lead of **3**, its cytotoxicity was also lower than compound **3**. Substitution of Cl group at R^2^ position decreased anti-DENV activity, in which **16b** was inactive (9% inhibition) at a concentration of 10 μM, while the unsubstituted **15b** exhibited 48% inhibition of DENV-2 at the same concentration. Substitution of F group at R^1^ position decreased anti-DENV activity in which **17b** (22% inhibition) was much less active than the unsubstituted **15b,** indicating the electron-withdrawing group at R^1^ position was unfavorable. Contrarily, introduction of the electron-donating MeO group at R^1^ position (**15a** vs. **19a**) strongly enhanced antiviral activity. [3-(4-Methoxyphenyl)quinoxalin-2-yl](phenyl)methanol (**19a**), whose structure belongs to the 4-aza analog of **3**, was the most active (82% inhibition) among these newly synthesized 3-arylquinoxaline derivatives. It is worth mentioning that compound **19a** was non-cytotoxic to Huh-7 cells, exhibiting 85% cell viability at a concentration of 200 μM. Further substitution of the MeO group at R^3^ position (**19b** vs. **19a**) or Cl group at R^2^ position (**20a** vs. **19a**) decreased antiviral activity. Compound **20b** was inactive, which bears MeO groups at both R^1^ and R^3^ positions and also a Cl group at R^2^ position. These results indicated that the optimal electron environment is important, in which the electron-donating MeO group may be substituted at either R^1^ or R^3^ but not both positions. For the 2-carbonyl derivatives, phenyl(3-phenylquinoxalin-2-yl)methanone (**21a**) was inactive. Introduction of the electron-donating MeO group at R^3^ position (**21b** vs. **21a**) strongly enhanced anti-DENV activity. Compound **21a** (R^3^ = H) was inactive, while **21b** (R^3^ = OMe) was highly active, exhibited 51% inhibition of DENV-2 at a concentration of 10 μM, and was non-cytotoxic against Huh-7 cells at 200 μM. Introduction of Cl group at R^2^ position (**21b** vs. **22b**) or F group at R^1^ position (**21b** vs. **23b**) decreased antiviral activity. Introduction of the MeO group at R^1^ position (**21a** vs. **25a**) strongly enhanced antiviral activity, indicating the electron-donating group at R^1^ position was favorable. Compound **26b** was inactive, which bears MeO groups at both R^1^ and R^3^ positions and also a Cl group at R^2^ position.

In general, the 2-hydroxymethyl derivatives were less active than their respective 2-carbonyl counterparts (**15b** < **21b**; **16a** < **22a**; **16b** < **22b**; **17a** < **23a**; **17b** < **23b**; **19b** < **25b**) with an exception of **19a**, which was more active than **25a**. Compounds **19a**, **20a**, and **21b** were three of the most active antiviral agents, exhibiting >50% inhibition of DENV-2 at a concentration of 10 μM, and were selected for further evaluation. The effective concentration that inhibited 50% DENV replication (EC_50_), the concentration that inhibited 50% cell growth (CC_50_), and the selective index (SI: CC_50_/EC_50_) of these compounds were determined with our initial leads **3** and **4**, using ribavirin as a positive control. Results indicated that compounds **19a, 20a**, and **21b** were superior to compounds **3** and **4** and more active than ribavirin (Table 2). Among them, compound **19a** was the most active (EC_50_ of 1.29 μM), exhibiting approximately 10-fold more anti-DENV activity than that of ribavirin (EC_50_ = 13.16 μM). In addition, compound **19a** was less cytotoxic than ribavirin. The selective index (SI) of **19a** was approximately 35-fold higher than that of ribavirin (155.04 vs. 4.47). Based on the above results, compound **19a** was selected as a lead compound for further pharmacological studies.

#### 2.2.2. Compound **19a** Reduced DENV Replication in Huh-7 cells

**Figure 2:**
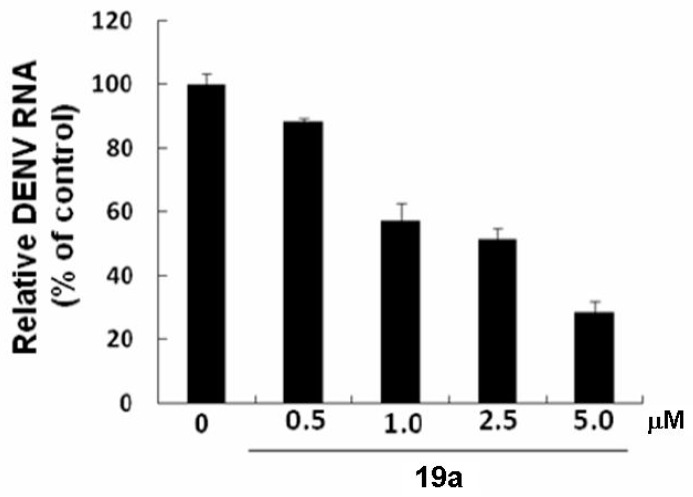
Inhibition of dengue virus (DENV) RNA expression in DENV-infected Huh-7 cells by **19a**. Huh-7 cells were treated with 0.5, 1, 5, and 10 μM **19a** for three days. Total RNA was extracted and quantified DENV RNA levels by RT-qPCR. DENV RNA expression was normalized by cellular glyceraldehyde-3-phosphate dehydrogenase (GAPDH) mRNA. Treatment with 0.1% dimethyl sulfoxide (DMSO) served as a mock control. The results are expressed as the means ± standard deviations (SD) of triplicate experiments.

**Figure 3:**
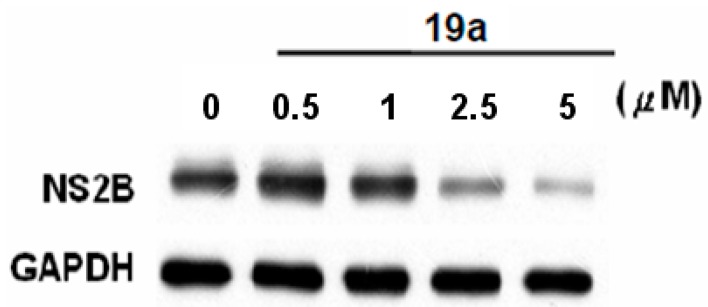
Inhibition of DENV protein synthesis in Huh-7 cells by **19a**. Huh-7 cells were treated with 0.5, 1, 2.5, and 5 μM **19a** for three days. Total cell lysate was collected for performing Western blotting to analyze DENV protein synthesis. Levels of GAPDH were used as equal loading control.

To further confirm the anti-DENV effect of compound **19a**, we treated compound **19a** at indicated concentrations in Huh-7 cells for three days. Both Western blotting and RT-qPCR were performed to determine the activity of compound **19a** against DENV replication, and the results showed that compound **19a** dose-dependently reduced DENV protein synthesis and RNA replication in Huh-7 cells (Figure 2 and Figure 3).

#### 2.2.3. Compound **19a** Reduced DENV Replication through Inhibiting COX-2 Expression

**Figure 4:**
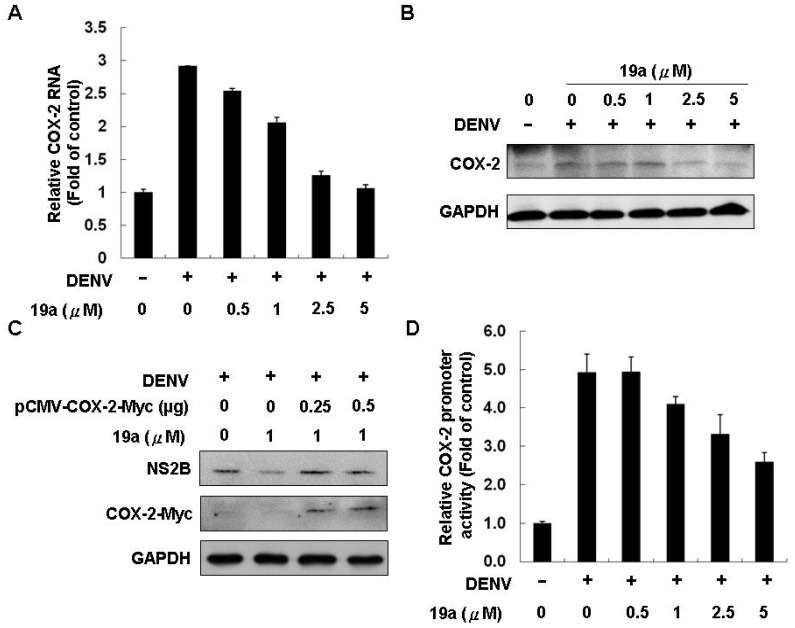
Inhibitory effect of **19a** on DENV-induced cyclooxygenase-2 (COX-2) expression in Huh-7 cells. Concentration-dependent reduction of DENV-induced COX-2 RNA replication (**A**) and protein expression (**B**) by **19a**. Huh-7 cells were treated with **19a** at the indicated concentrations for three days. Cell lysates were subjected to Western blotting and RT-qPCR. (**C**) Concentration-dependent restoration of the DENV protein level. Cell lysates were subjected to Western blotting with an antibody against NS2B, Myc, or GAPDH. (**D**) Concentration-dependent reduction of DENV-induced *COX-2* gene promoter activity by **19a** in Huh-7 cells. Huh-7 cells were transfected with pCOX-2-Luc reporter plasmid encoding firefly luciferase under the control of the COX-2 promoter. After treatment with **19a** at the indicated concentrations for three days, cell lysates were subjected to a luciferase activity assay. The results are expressed as the means ± standard deviations (SD) of triplicate experiments.

In a previous study, Lin et al. reported that induction of COX-2 could suppress DENV replication [11]. To determine whether compound **19a** had impact on COX-2 protein and mRNA expression in Huh-7 cells, we treated Huh-7 cells with 0.5 to 5 μM of **19a**. The results showed that compound **19a** could inhibit COX-2 protein and mRNA level in Huh-7 cells, compared with the DMSO-treated Huh-7 cells (Figure 4A,B). To characterize whether the **19a**-mediated downregulation of COX-2 expression and its catalytic activity was involved in the suppression of DENV replication, we overexpressed exogenous COX-2 to evaluate the inhibitory activity of **19a** on DENV replication. Huh-7 cells were transfected with vehicle or various concentrations of cytomegalovirus (pCMV)-COX-2-Myc vector encoding the *cox-2* gene, and the plasmid-transfected cells were incubated with 1 μM **19a** or 0.1% DMSO, as a negative control. As shown in Figure 4C, the **19a**-reduced viral RNA level was gradually rescued following the increasing expression of exogenous COX-2-Myc compared with that in the vehicle-transfected Huh-7 cells incubated with DMSO. Consistently, the exogenous expression of COX-2 attenuated the inhibitory effect of **19a** on the non-structural 2B (NS2B) protein levels, as analyzed by Western blotting.

These results clearly indicated that **19a** inhibited DENV replication by downregulating the virus-induced COX-2 expression. We also used a COX-2 promoter-based reporter assay to evaluate the inhibitory effect of **19a** on COX-2 at the transcriptional level. The pCOX-2-Luc, a plasmid encoding firefly luciferase under the control of the COX-2 promoter, was transfected into Huh-7 cells or DENV-infected Huh-7 cells and, then, these plasmid-transfected cells were incubated with **19a** at increasing concentrations for three days. As shown in Figure 4D, **19a** dose-dependently decreased the DENV-elevated COX-2 promoter activity in DENV-infected cells.

### 2.3. Molecular Docking

**Figure 5:**
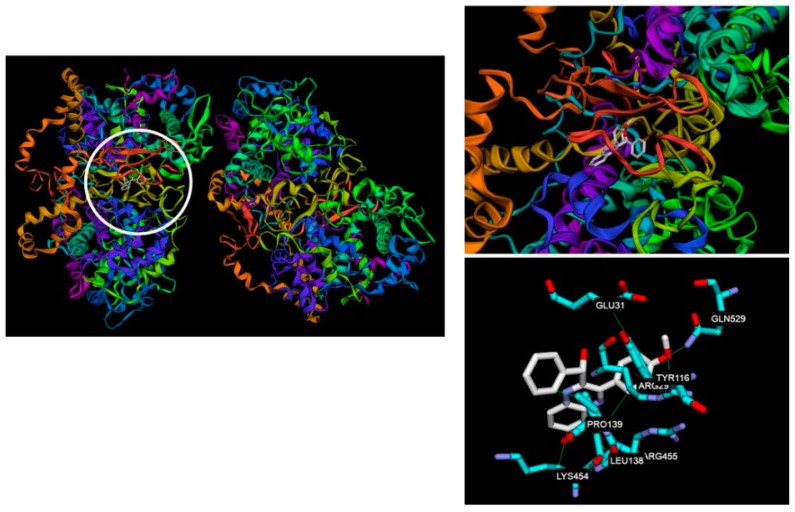
Docking poses of compound **19a** at COX-2 (PDB code 3LN1) enzyme. The interactions illustrated by green lines represent hydrophobic interactions, blue lines represent hydrogen bonds, and red lines represent π–cation interactions.

In order to explain the binding mode and interaction, molecule docking of **19a** was performed against COX-2 (Protein Data Bank (PDB) code 3LN1) enzyme. Figure 5 shows the three-dimensional (3D) molecular interaction of **19a** against COX-2. The binding between **19a** and COX-2 was stabilized through hydrophobic interactions, hydrogen binding, and π–cation interactions. The amino acids involved including **19a** caused hydrophobic interactions at the active sites with Arg29, Glu31, Tyr116, Leu138, Pro139, Lys454, Arg455, and Gln529. Arg29, located in the binding pocket, played a vital role in the conformation of **19a**, causing three kinds of interaction. The calculated lowest binding energy between **19a** and COX-2 was −9.10 kcal/mol.

## 3. Materials and Methods

### 3.1. Chemistry Section

Melting points were determined on an Electrothermal IA9100 melting point apparatus (Electrothermal, Staordshire, UK). Nuclear magnetic resonance (^1^H) spectra were recorded on a Varian-Unity-400 spectrometer (LabX, Canada). Chemical shifts were expressed in parts per million (δ) with tetramethylsilane (TMS) as an internal standard. Thin-layer chromatography (TLC) was performed on silica gel 60 F-254 plates purchased from E. Merck KG (Darmstadt, Germany). The elemental analyses were performed in the Instrument Center of National Science Council at National Cheng-Kung University and National Chung-Hsing University using Heraeus CHN-O Rapid EA (Labexchange, Burladingen, Germany), and all values were within ±0.4% of the theoretical compositions. 

#### 3.1.1. General Procedure for the Preparation of 2-Methyl-3-phenylquinoxalines **9a**–**11b**

Compound **6** (2.0 mmol) and appropriate benzene-1,2-diamine (2.0 mmol) were refluxed under (H_2_O:EtOH = 1:1) for 3 h (TLC monitoring). After cooling, the solvent was removed in vacuo to provide the crude product, which was purified by flash column chromatography on silica gel (CH_2_Cl_2_/MeOH 100/1) and recrystallized with EtOH to give 2-methyl-3-phenylquinoxalines **9a** and **9b**. Compounds **10a**–**11b** were prepared from **7** or **8** (2.0 mmol) and appropriate benzene-1,2-diamine (2.0 mmol) under the same reaction procedures [23].

#### 3.1.2. General Procedure for the Preparation of 3-Phenylquinoxaline-2-carbaldehydes **12a**–**14b**

A mixture of **9a** (3.0 mmol) and selenium dioxide (0.66 g, 6.0 mmol) in 1,4-dioxane (50 mL) was refluxed for 3 h (TLC monitoring) and then cooled to room temperature. The reaction mixture was filtered through celite to remove the black residue. Evaporation of the solvent afforded a residue which was dissolved in ethyl acetate (200 mL), washed with brine (100 mL), H_2_O (100 mL), and saturated sodium bicarbonate solution (100 mL), and dried (MgSO_4_). The crude product was recrystallized with EtOH to give 3-phenylquinoxaline-2-carbaldehydes **12a** and **12b**. Compounds **13a**–**14b** were prepared from **10a**–**11b** (3.0 mmol) under the same reaction procedures [24].

#### 3.1.3. General Procedure for the Preparation of Phenyl(3-phenylquinoxalin-2-yl)methanols **15a**–**20b**

A mixture of 3-phenylquinoxaline-2-carbaldehyde **12a** (1.0 mmol), appropriate phenyl magnesium bromide (3 mmol, 3 mL of a 1 M solution in tetrahydrofuran (THF)), and dry THF (30 mL) was stirred at 0 °C for 12 h (TLC monitoring). The reaction was quenched by addition of water (3 mL) and partitioned between H_2_O (50 mL) and CH_2_Cl_2_ (50 mL). The organic layer was washed with brine and dried over MgSO_4_, and removal of the volatiles in vacuo provided a residue, which was purified by flash chromatography on silica gel (*n*-hexane: ethyl acetate = 3/2) and recrystallized from EtOH to give compounds **15a** or **15b**. Accordingly, compounds **16a**–**20b** were obtained in the same manner.

##### Phenyl(3-phenylquinoxalin-2-yl)methanol (**15a**)

Yield 40%, Melting point (Mp): 146.0–147.0 °C. ^1^H NMR (400 MHz, CDCl_3_) δ 5.58 (d, 1H, *J* = 7.2 Hz, OH), 6.13 (d, 1H, *J* = 7.2 Hz, CHOH), 6.80 (dd, 2H, *J*_1_ = 8.0 Hz, *J*_2_ = 1.6 Hz), 7.10 (m, 3H), 7.27 (m, 2H), 7.42 (m, 3H), 7.82 (m, 2H), 8.16 (m, 1H), 8.22 (m, 1H). ^13^C NMR (100 MHz, CDCl_3_) δ 72.87, 127.56 (2C), 127.73, 128.23 (2C), 128.53, 128.55 (2C), 128.76 (2C), 129.13, 129.33, 130.21, 130.32, 137.57, 139.52, 141.19, 141.61, 154.16, 154.32. Analysis calculated for C_21_H_16_N_2_O: C 80.75, H 5.16, N 8.97; found C 80.55, H 5.10, N 8.90.

##### (4-Methoxyphenyl)(3-phenylquinoxalin-2-yl)methanol (**15b**)

Yield 59%, Mp: 166.0–168.6 °C. ^1^H NMR (400 MHz, CDCl_3_) δ 3.71 (s, 3H, OCH_3_), 5.51 (br s, 1H, OH), 6.07 (s, 1H, CHOH), 6.62 (d, 2H, *J* = 8.8 Hz), 6.72 (d, 2H, *J* = 8.8 Hz), 7.26 (d, 2H, *J* = 8.4 Hz), 7.44 (m, 3H), 7.82 (m, 2H), 8.16 (m, 1H), 8.22 (m, 1H). ^13^C NMR (100 MHz, CDCl_3_) δ 55.18, 72.37, 113.69 (2C), 128.52 (3C), 128.74 (2C), 128.84 (2C), 129.09, 129.32, 130.13, 130.28, 133.51, 137.63, 139.52, 141.56, 154.19, 154.59, 159.12. Analysis calculated for C_22_H_18_N_2_O_2_**∙**0.1H_2_O: C 76.76, H 5.34, N 8.14; found C 76.39, H 5.46, N 8.27.

##### (6,7-Dichloro-3-phenylquinoxalin-2-yl)(phenyl)methanol (**16a**)

Yield 40%, Mp: 136.4–136.9 °C. ^1^H NMR (400 MHz, CDCl_3_) δ 5.24 (d, 1H, *J* = 7.2 Hz, OH), 6.13 (d, 1H, *J* = 6.8 Hz, CHOH), 6.78 (m, 2H), 7.11 (m, 3H), 7.27 (m, 2H), 7.42 (m, 3H), 8.27 (s, 1H), 8.35 (s, 1H). ^13^C NMR (100 MHz, CDCl_3_) δ 72.95, 127.46 (2C), 127.98, 128.37 (2C), 128.65 (2C), 128.67 (2C), 129.18, 129.54, 129.96, 134.92, 135.03, 136.90, 138.33, 140.35, 140.64, 155.21, 155.79. Analysis calculated for C_21_H_14_Cl_2_N_2_O**∙**0.1H_2_O: C 65.82, H 3.74, N 7.31; found C 65.67, H 3.80, N 7.52.

##### (6,7-Dichloro-3-phenylquinoxalin-2-yl)(4-methoxyphenyl)methanol (**16b**)

Yield 37%, Mp: 157.2–157.6 °C. ^1^H NMR (400 MHz, CDCl_3_) δ 3.72 (s, 3H, OCH_3_), 5.16 (d, 1H, *J* = 7.2 Hz, OH), 6.07 (d, 1H, *J* = 7.2 Hz, CHOH), 6.63 (d, 2H, *J* = 8.8 Hz), 6.70 (d, 2H, *J* = 8.8 Hz), 7.26 (d, 2H, *J* = 8.4 Hz), 7.44 (m, 3H), 8.26 (s, 1H), 8.35 (s, 1H). ^13^C NMR (100 MHz, CDCl_3_) δ 55.19, 72.45, 113.80 (2C), 128.62 (2C), 128.65 (2C), 128.77 (2C), 129.16, 129.50, 129.95, 132.89, 134.8, 134.97, 136.96, 138.34, 140.30, 155.25, 156.05, 159.26. Analysis calculated for C_22_H_16_Cl_2_N_2_O_2_: C 64.22, H 3.92, N 6.81; found C 63.84, H 3.88, N 6.76.

##### [3-(4-Fluorophenyl)quinoxalin-2-yl](phenyl)methanol (**17a**)

Yield 41%, Mp: 157.4–158.0 °C. ^1^H NMR (400 MHz, CDCl_3_) δ 5.79 (d, 1H, *J* = 6.8 Hz, OH), 6.07 (d, 1H, *J* = 6.4 Hz, CHOH), 6.82 (m, 2H), 7.11 (m, 5H), 7.25 (m, 2H), 7.84 (m, 2H), 8.15 (m, 1H), 8.23 (m, 1H). ^13^C NMR (100 MHz, CDCl_3_) δ 72.98, 115.63 (d, *J*_CF_ = 22 Hz, 2C), 127.58 (2C), 127.91, 128.37 (2C), 128.55, 129.27, 130.37, 130.50, 130.74 (d, *J*_CF_ = 8.3 Hz, 2C), 133.68 (d, *J*_CF_ = 3.8 Hz), 139.52, 141.07, 141.56, 153.16, 154.18, 163.30 (d, *J*_CF_ = 267.1 Hz). Analysis calculated for C_21_H_15_FN_2_O**∙**0.1H_2_O: C 75.93, H 4.62, N 8.44; found C 75.64, H 4.54, N 8.45.

##### [3-(4-Fluorophenyl)quinoxalin-2-yl](4-methoxyphenyl)methanol (**17b**)

Yield 78%, Mp: 173.0–174.7 °C. ^1^H NMR (400 MHz, CDCl_3_) δ 3.72 (s, 3H, OCH_3_), 6.02 (s, 1H, CHOH), 6.65 (d, 2H, *J* = 8.8 Hz), 6.75 (d, 2H, *J* = 8.8 Hz), 7.10 (m, 2H), 7.25 (m, 2H), 7.85 (m, 2H), 8.14 (m, 1H), 8.23 (m, 1H). ^13^C NMR (100 MHz, CDCl_3_) δ 55.20, 72.44, 113.77 (2C), 115.60 (d, *J*_CF_ = 21.7 Hz, 2C), 128.52, 128.87 (2C), 129.24, 130.29, 130.47, 130.70 (d, *J*_CF_ = 8.4 Hz), 133.30, 133.71 (d, *J*_CF_ = 3.4 Hz, 2C), 139.49, 141.48, 153.17, 154.41, 159.20, 163.24 (d, *J*_CF_ = 247.8 Hz). Analysis calculated for C_22_H_17_FN_2_O_2_**∙**0.2H_2_O: C 72.59, H 4.83, N 7.70; found C 72.43, H 4.73, N 7.73.

##### [6,7-Dichloro-3-(4-fluorophenyl)quinoxalin-2-yl](phenyl)methanol (**18a**)

Yield 52%, Mp: 150.0–150.2 °C. ^1^H NMR (400 MHz, CDCl_3_) δ 5.22 (br s, 1H, OH), 6.07 (s, 1H, CHOH), 6.81 (m, 2H), 7.13 (m, 5H), 7.25 (m, 2H), 8.12 (s, 1H), 8.36 (s, 1H). ^13^C NMR (100 MHz, CDCl_3_): 73.06, 115.79 (d, *J*_CF_ = 21.8 Hz, 2C), 127.49 (2C), 128.15, 128.51 (2C), 129.18, 129.90, 130.73 (d, *J*_CF_ = 8.4, 2C), 133.02 (d, *J*_CF_ = 3.4), 135.11, 135.25, 138.33, 140.28, 140.54, 154.20, 155.65, 163.49 (d, *J*_CF_ = 248.5 Hz). Analysis calculated for C_21_H_13_Cl_2_FN_2_O: C 63.18, H 3.28, N 7.02; found C 63.29, H 3.06, N 7.07.

##### [3-(4-Methoxyphenyl)quinoxalin-2-yl](phenyl)methanol (**19a**)

Yield 48%, Mp: 160.4–161.9 °C. ^1^H NMR (400 MHz, CDCl_3_) δ 3.87 (s, 3H, OCH_3_), 5.55 (d, 1H, *J* = 7.2 Hz, OH), 6.16 (d, 1H, *J* = 7.2 Hz, CHOH), 6.86 (m, 2H), 6.93 (m, 2H), 7.12 (m, 3H), 7.25 (m, 2H), 7.80 (m, 2H), 8.14 (m, 1H), 8.21 (m, 1H). ^13^C NMR (100 MHz, CDCl_3_) δ 55.46, 72.91, 114.14 (2C), 127.42 (2C), 127.99, 128.40 (2C), 129.12, 129.30, 129.86, 130.28 (2C), 134.69, 134.79, 138.17, 140.43, 140.88, 154.90, 155.95, 160.75. Analysis calculated for C_22_H_18_N_2_O_2_: C 77.17, H 5.30, N 8.18; found C 77.02, H 5.23, N 8.10.

##### (4-Methoxyphenyl)[3-(4-methoxyphenyl)quinoxalin-2-yl]methanol (**19b**)

Yield 78%, Mp: 132.4–136.0 °C. ^1^H NMR (400 MHz, CDCl_3_) δ 3.72 (s, 3H, OCH_3_), 3.87 (s, 3H, OCH_3_), 5.45 (d, 1H, *J* = 7.6 Hz, OH), 6.10 (d, 1H, *J* = 7.2 Hz, CHOH), 6.65 (d, 2H, *J* = 8.8 Hz), 6.89 (d, 2H, *J* = 8.8 Hz), 6.93 (d, 2H, *J* = 8.8 Hz), 7.25 (d, 2H, *J* = 8.8 Hz), 7.80 (m, 2H), 8.14 (m, 1H), 8.19 (m, 1H). ^13^C NMR (100 MHz, CDCl_3_) δ 55.19, 55.42, 72.34, 113.71 (2C), 114.01 (2C), 128.46, 128.80 (2C), 129.23, 130.03 (2C), 130.09, 130.26 (2C), 133.73, 139.39, 141.63, 153.89, 154.74, 159.12, 160.41. Analysis calculated for C_23_H_20_N_2_O_3_: C 74.18, H 5.41, N 7.52; found C 73.92, H 5.33, N 7.47.

##### [6,7-Dichloro-3-(4-methoxyphenyl)quinoxalin-2-yl](phenyl)methanol (**20a**) 

Yield 55%, Mp: 156.8–157.1 °C. ^1^H NMR (400 MHz, CDCl_3_) δ 3.88 (s, 3H, OCH_3_), 5.22 (d, 1H, *J* = 7.2 Hz, OH), 6.16 (d, 1H, *J* = 6.0 Hz, CHOH), 6.85 (dd, 2H, *J*_1_ = 8.8 Hz, *J*_2_ = 2.0 Hz), 6.94 (d, 2H, *J* = 8.8 Hz), 7.15 (m, 3H), 7.28 (d, 2H, *J* = 8.8 Hz), 8.25 (s, 1H), 8.33 (s, 1H). ^13^C NMR (100 MHz, CDCl_3_) δ 55.46, 72.91, 114.14 (2C), 127.42 (2C), 127.99, 128.40 (2C), 129.12, 129.30, 129.86, 130.28 (2C), 134.69, 134.79, 138.17, 140.43, 140.88, 154.90, 155.95, 160.75. Analysis calculated for C_22_H_16_Cl_2_N_2_O_2_**∙**0.1H_2_O: C 63.94, H 3.96, N 6.78; found C 63.75, H 3.88, N 6.77.

##### [6,7-Dichloro-3-(4-methoxyphenyl)quinoxalin-2-yl](4-methoxyphenyl)methanol (**20b**)

Yield 56%, Mp: 144.1–144.5 °C. ^1^H NMR (400 MHz, CDCl_3_) δ 3.72 (s, 3H, OCH_3_), 3.88 (s, 3H, OCH_3_), 5.13 (d, 1H, *J* = 7.6 Hz, OH), 6.11 (d, 1H, *J* = 6.8 Hz, CHOH), 6.66 (d, 2H, *J* = 8.8 Hz), 6.78 (d, 2H, *J* = 8.8 Hz), 6.94 (d, 2H, *J* = 8.8 Hz), 7.26 (d, 2H, *J* = 8.8 Hz), 8.24 (s, 1H), 8.32 (s, 1H). ^13^C NMR (100 MHz, CDCl_3_) δ 55.20, 55.45, 72.42, 113.83 (2C), 114.10 (2C), 128.74 (2C), 129.11, 129.36, 129.85, 130.28 (2C), 133.14, 134.62, 134.67, 138.18, 140.38, 154.91, 156.19, 159.26, 160.71. Analysis calculated for C_23_H_18_Cl_2_N_2_O_3_: C 62.60, H 4.11, N 6.35; found C 62.40, H 4.00, N 6.42.

#### 3.1.4. General procedure for the Preparation of Phenyl(3-phenylquinoxalin-2-yl)methanones (**21a**–**26b**)

A mixture of **15a** (1.0 mmol) and MnO_2_ (10.0 mmol) in CH_2_Cl_2_ (20 mL) was stirred at room temperature for 12 h (TLC monitoring). The reaction mixture was partitioned between H_2_O (50 mL) and CH_2_Cl_2_ (50 mL). The organic layer was washed with brine and dried over MgSO_4_, and removal of the volatiles in vacuo provided a residue, which was crystallized from MeOH to give compound **21a**. Accordingly, compounds **21b**–**26b** were obtained in this manner.

##### Phenyl(3-phenylquinoxalin-2-yl)methanone (**21a**)

Yield 89%, Mp: 155.2–156.7 °C. ^1^H NMR (400 MHz, CDCl_3_) δ 7.38 (m, 3H), 7.47 (m, 2H), 7.461 (tt, 1H, *J*_1_ *=* 7.6 Hz, *J*_2_ *=* 1.6 Hz), 7.70 (m, 2H), 7.82 (m, 1H), 7.88(m, 1H), 7.94 (m, 2H), 8.16 (m, 1H), 8.24 (m, 1H). ^13^C NMR (100 MHz, CDCl_3_) δ 128.70 (4C), 129.11 (2C), 129.46, 129.49, 129.59, 130.44, 130.52 (2C), 131.40, 134.05, 135.62, 137.27, 139.65, 142.14, 151.31, 152.81, 193.98. Analysis calculated for C_21_H_14_N_2_O 0.3H_2_O: C 79.87, H 4.67, N 8.87; found C 79.75, H 4.46, N 8.92.

##### (4-Methoxyphenyl)(3-phenylquinoxalin-2-yl)methanone (**21b**)

Yield 70%, Mp: 130.0–132.4 °C. ^1^H NMR (400 MHz, CDCl_3_) δ 3.88 (s, 3H, OCH_3_), 6.94 (m, 2H), 7.39 (m, 3H), 7.72 (dd, 2H, *J*_1_ *=* 6.0 Hz, *J*_2_ *=* 2.4 Hz), 7.81 (m, 1H), 7.87 (m, 1H), 7.92 (m, 2H), 8.16 (m, 1H), 8.23 (m, 1H). ^13^C NMR (100 MHz, CDCl_3_) δ 55.56, 114.05 (2C), 128.67 (2C), 128.72, 129.06 (2C), 129.40, 129.46, 129.56, 130.35, 131.22, 132.95 (2C), 137.36, 139.66, 142.08, 151.64, 152.75, 164.36, 192.58 Analysis calculated for C_22_H_16_N_2_O_2_**∙**0.2H_2_O: C 76.81, H 4.82, N 8.15; found C 76.76, H 4.72, N 8.08.

##### (6,7-Dichloro-3-phenylquinoxalin-2-yl)(phenyl)methanone (**22a**)

Yield 66%, Mp: 154.3–156.8 °C. ^1^H NMR (400 MHz, CDCl_3_) δ 7.40 (m, 3H), 7.49 (m, 2H), 7.64 (tt, 1H, *J*_1_ = 7.6 Hz, *J*_2_ = 1.2 Hz), 7.68 (m, 2H), 7.93 (m, 2H), 8.28 (s, 1H), 8.36 (s, 1H). ^13^C NMR (100 MHz, CDCl_3_) δ 128.81 (4C), 129.09 (2C), 129.96, 130.02, 130.09, 130.48 (2C), 134.33, 135.17, 135.25, 136.23, 136.52, 138.27, 140.84, 152.18, 153.84, 193.31. Analysis calculated for C_21_H_12_Cl_2_N_2_O: C 66.51, H 3.19, N 7.39; found C 66.61, H 3.00, N 7.38.

##### (6,7-Dichloro-3-phenylquinoxalin-2-yl)(4-methoxyphenyl)methanone (**22b**)

Yield 79%, Mp: 171.9–172.3 °C. ^1^H NMR (400 MHz, CDCl_3_) δ 3.89 (s, 3H, OCH_3_), 6.96 (m, 2H), 7.41 (m, 3H), 7.70 (dd, 2H, *J*_1_ = 8.0 Hz, *J*_2_ = 1.6 Hz), 7.91 (m, 2H), 8.28 (s, 1H), 8.35 (s, 1H). ^13^C NMR (100 MHz, CDCl_3_) δ 55.62, 114.17 (2C), 128.31, 128.77 (2C), 129.04 (2C), 129.91, 129.99, 130.06, 132.94 (2C), 135.03, 136.01, 136.61, 138.30, 140.77, 152.52, 153.80, 164.57, 191.90 Analysis calculated for C_22_H_14_Cl_2_N_2_O_2_: C 64.56, H 3.45, N 6.84; found C 64.19, H 3.46, N 6.85.

##### [3-(4-Fluorophenyl)quinoxalin-2-yl](phenyl)methanone (**23a**)

Yield 85%, Mp: 94.1–96.6 °C. ^1^H NMR (400 MHz, CDCl_3_) δ 7.08 (tt, 2H, *J* = 8.4, 2.0 Hz), 7.49 (tt, 2H, *J* = 8, 1.6 Hz), 7.64 (tt, 1H, *J* = 7.6, 1.6 Hz), 7.66 (m, 2H), 7.90 (br s, 1H), 7.93 (m, 2H), 7.96 (br s, 1H). ^13^C NMR (100 MHz, CDCl_3_) δ 115.87 (d, *J*_CF_ = 22 Hz, 2C), 128.79 (2C), 129.23 (d, *J*_CF_ = 6.8 Hz, 2C), 130.53 (2C), 130.57, 131.07, 131.16, 131.56, 133.37 (d, *J*_CF_ = 3.0 Hz), 134.23, 135.46, 139.62, 142.06, 151.02, 151.66, 163.67 (d, *J*_CF_ = 248.7 Hz), 193.95. Analysis calculated for C_21_H_13_FN_2_O: C 76.82, H 3.99, N 8.53; found C 76.44, H 3.86, N 8.55.

##### [3-(4-Fluorophenyl)quinoxalin-2-yl](4-methoxyphenyl)methanone (**23b**)

Yield 83%, Mp: 147.6–147.9 °C. ^1^H NMR (400 MHz, CDCl_3_) δ 3.89 (s, 3H, OCH_3_), 6.95 (d, 2H, *J* = 9.2 Hz), 7.08 (m, 2H), 7.72 (m, 2H), 7.83 (m, 1H), 7.88 (m, 1H), 7.92 (m, 2H), 8.16 (m, 1H), 8.21 (m, 1H). ^13^C NMR (100 MHz, CDCl_3_) δ 55.59, 114.13 (2C), 115.84 (d, *J*_CF_ = 21.8 Hz, 2C), 128.51, 129.37, 129.40, 130.49, 131.06 (d, *J*_CF_ = 8.7 Hz, 2C), 131.38, 132.98 (2C), 133.46 (d, *J*_CF_ = 3.5 Hz), 139.63, 141.98, 151.37, 151.60, 163.65 (d, *J*_CF_ = 248.2 Hz), 164.48, 192.55. Analysis calculated for C_22_H_15_FN_2_O_2_**∙**0.1H_2_O: C 73.35, H 4.26, N 7.78; found C 73.06, H 4.27, N 7.77.

##### (6,7-Dichloro-3-(4-fluorophenyl)quinoxalin-2-yl)(phenyl)methanone (**24a**)

Yield 75%, Mp: 184.3–186.4 °C. ^1^H NMR (400 MHz, CDCl_3_) δ 7.08 (t, 2H, *J* = 8.8 Hz), 7.50 (t, 2H, *J* = 8 Hz), 7.64-7.70 (m, 3H), 7.92 (dd, 2H, *J*_1_ = 8.0 Hz, *J*_2_ = 1.6 Hz), 8.27 (s, 1H), 8.33 (s, 1H). ^13^C NMR (100 MHz, CDCl_3_) δ 116.03 (d, *J*_CF_ = 22 Hz, 2C), 128.90 (2C), 129.93, 129.95, 130.48 (2C), 131.17 (d, *J*_CF_ = 8.8 Hz, 2C), 132.63 (d, *J*_CF_ = 3.0 Hz), 134.51, 135.09, 135.29, 136.40, 138.23, 140.74, 151.87, 152.65, 163.93 (d, *J*_CF_ = 249.7 Hz), 193.28. Analysis calculated for C_21_H_11_Cl_2_FN_2_O: C 63.50, H 2.79, N 7.05; found C 63.37, H 2.68, N 6.96.

##### [3-(4-Methoxyphenyl)quinoxalin-2-yl](phenyl)methanone (**25a**)

Yield 91%, Mp: 93.9–98.2 °C. ^1^H NMR (400 MHz, CDCl_3_) δ 3.80 (s, 3H, OCH_3_), 6.90 (m, 2H), 7.47 (m, 2H), 7.61 (tt, 1H, *J*_1_ = 7.6 Hz, *J*_2_ = 1.6 Hz), 7.68 (m, 2H), 7.79 (m, 1H), 7.86 (m, 1H), 7.94 (m, 2H), 8.14 (m, 1H), 8.21 (m, 1H). ^13^C NMR (100 MHz, CDCl_3_) δ 55.30, 114.29 (2C), 128.72 (2C), 129.33, 129.39, 129.64, 130.05, 130.52 (2C), 130.65 (2C), 131.28, 134.06, 135.61, 139.41, 142.20, 151.20, 152.19, 160.88, 194.26. Analysis calculated for C_22_H_16_N_2_O_2_**∙**0.5H_2_O: C 75.62, H 4.91, N 8.02; found C 75.23, H 4.87, N 7.97.

##### (4-Methoxyphenyl)[3-(4-methoxyphenyl)quinoxalin-2-yl]methanone (**25b**)

Yield 76%, Mp: 144.5–146.2 °C. ^1^H NMR (400 MHz, CDCl_3_) δ 3.80 (s, 3H, OCH_3_), 3.87 (s, 3H, OCH_3_), 6.90 (d, *J* = 9.2 Hz), 6.94 (d, *J* = 9.2 Hz), 7.70 (d, *J* = 8.8 Hz), 7.78 (td, *J*_1_ = 6.8 Hz, *J*_2_ = 1.2 Hz, 1H), 7.84 (td, *J*_1_ = 6.8 Hz, *J*_2_ = 1.2 Hz, 1H), 7.92 (d, *J* = 8.8 Hz), 8.13 (dd, *J*_1_ = 8.0 Hz, *J*_2_ = 1.2 Hz, 1H), 8.20 (dd, *J*_1_ = 8.0 Hz, *J*_2_ = 1.2 Hz, 1H). ^13^C NMR (100 MHz, CDCl_3_) δ 55.27, 55.55, 114.06 (2C), 114.21 (2C), 128.64, 129.26, 129.30, 129.68, 129.97, 130.57 (2C), 131.11, 132.93 (2C), 139.38, 142.09, 151.50, 152.10, 160.80, 164.34, 192.88. Analysis calculated for C_23_H_18_N_2_O_3_: C 74.58, H 4.90, N 7.56; found C 74.54, H 4.86, N 7.60.

##### [6,7-Dichloro-3-(4-methoxyphenyl)quinoxalin-2-yl](phenyl)methanone (**26a**)

Yield 41%, Mp: 173.9–174.3 °C. ^1^H NMR (400 MHz, CDCl_3_) δ 3.81 (s, 3H, OCH_3_), 6.91 (d, 2H, *J* = 8.8 Hz), 7.50 (t, 2H, *J* = 7.6 Hz), 7.66 (m, 3H), 7.93 (dd, 2H, *J*_1_ = 8.4 Hz, *J*_2_ = 1.2 Hz), 8.25 (s, 1H), 8.32 (s, 1H). ^13^C NMR (100 MHz, CDCl_3_) δ 55.36, 114.39 (2C), 128.85 (2C), 129.85, 129.89, 130.49 (2C), 130.73 (2C), 134.35, 134.63, 135.22, 136.04, 138.01, 140.90, 152.05, 153.16, 161.28, 193.62. Analysis calculated for C_22_H_14_Cl_2_N_2_O_2_**∙**0.3H_2_O: C 63.70, H 3.56, N 6.76; found C 63.58, H 3.45, N 6.75.

##### [6,7-Dichloro-3-(4-methoxyphenyl)quinoxalin-2-yl](4-methoxyphenyl)methanone (**26b**)

Yield 78%, Mp: 178.8–179.0 °C. ^1^H NMR (400 MHz, CDCl_3_) δ 3.81 (s, 3H, OCH_3_), 3.89 (s, 3H, OCH_3_), 6.90 (m, 2H), 6.95 (m, 2H), 7.68 (m, 2H), 7.90 (m, 2H), 8.24 (s, 1H), 8.31 (s, 1H). ^13^C NMR (100 MHz, CDCl_3_) δ 55.32, 55.61, 114.20 (2C), 114.33 (2C), 128.29, 128.91, 129.82, 129.84, 130.68 (2C), 132.93 (2C), 134.49, 135.80, 138.04, 140.84, 152.40, 153.11, 161.23, 164.57, 192.22. Analysis calculated for C_23_H_16_Cl_2_N_2_O_3_**∙**0.2H_2_O: C 62.64, H 3.30, N 6.35; found C 62.45, H 3.67, N 6.26.

#### 3.1.5. (4-Hydroxyphenyl)(3-phenylquinoxalin-2-yl)methanone (**27**)

A solution of **15b** (0.33 g, 1.0 mmol) in 48% HBr (5 mL) was heated at reflux for 48 h. The mixture was cooled and evaporated in vacuo to give a residue which was treated with H_2_O (50 mL). The crude product was collected and crystallized from MeOH to give **27** (0.29 g, 87%) as a white solid, Mp: 196.5–199.3 °C. ^1^H NMR (400 MHz, DMSO-*d_6_*) δ 6.88 (d, *J* = 8.8 Hz, 2H, 3”–H, 5”–H), 7.42–7.46 (m, 3H), 7.65–7.68 (m, 2H), 7.65 (d, *J* = 8.8 Hz, 2H, 2”–H, 6”–H), 7.92–8.01 (m, 2H), 8.15 (dd, *J*_1_ = 8.0 Hz, *J*_2_ = 1.6 Hz), 8.23 (dd, *J*_1_ = 8.0 Hz, *J*_2_ = 1.6 Hz), 10.78 (br s, 1H, Ph-OH). ^13^C NMR (100 MHz, DMSO-*d_6_*) δ 115.77 (2C), 126.87, 128.56 (2C), 128.84 (2C), 128.97, 129.12, 129.57, 130.94, 131.66, 133.16 (2C), 137.09, 138.99, 141.37, 151.77, 151.87, 163.35, 191.99. Analysis calculated for C_21_H_14_N_2_O_2_**∙**1.0H_2_O: C 73.05, H 4.31, N 8.12; found C 73.23, H 4.69, N 8.14.

#### 3.1.6. 4-[3-(4-Hydroxybenzyl)quinoxalin-2-yl]phenol (**28**)

Compound **28** was obtained from **19b** as described for **27** in 37% yield, Mp: 184.8–185.9 °C. ^1^H NMR (400 MHz, DMSO-*d_6_*) δ 4.30 (s, 2H, CH_2_), 6.58 (d, *J* = 10.8 Hz, 2H), 6.75 (d, *J* = 8.8 Hz, 2H), 7.87 (d, *J* = 8.4 Hz, 2H), 7.38 (dd, *J*_1_ = 1.6 Hz, *J*_2_ = 8.4 Hz, 2H), 7.75–7.79 (m, 2H), 8.01–8.06 (m, 2H), 9.12 (br s, 1H, Ph-OH), 9.74 (br s, 1H, Ph-OH). ^13^C NMR (100 MHz, DMSO-*d_6_*) δ 40.75, 114.91 (2C), 114.99 (2C), 128.23, 128.36, 128.53, 129.19, 129.26, 129.36 (2C), 130.36 (2C), 140.34, 154.77 (2C), 155.56 (2C), 158.07 (2C). Analysis calculated for C_21_H_16_N_2_O_2_: C 76.81, H 4.91, N 8.53; found C 76.48, H 4.69, N 8.23.

#### 3.1.7. (4-Hydroxyphenyl)(3-(4-hydroxyphenyl)quinoxalin-2-yl)methanone (**29**)

A mixture of **28** (0.33 g, 1.0 mmol) and selenium dioxide (0.22 g, 3.0 mmol) in 1,4-dioxane (20 mL) was refluxed for 6 h (TLC monitoring) and then cooled to room temperature. The reaction mixture was filtered through celite to remove the black residue. Evaporation of the solvent afforded a residue that was dissolved in ethyl acetate (200 mL), washed with brine (100 mL), H_2_O (100 mL), and saturated sodium bicarbonate solution (100 mL), and dried (MgSO_4_). The crude product was recrystallized with EtOH to give **29** (0.31 g, 90%) as a white solid, Mp: 254.0–254.6 °C. ^1^H NMR (400 MHz, DMSO-*d_6_*) δ 6.80 (d, *J* = 8.8 Hz, 2H, 3”–H, 5”–H), 6.88 (d, *J* = 8.8 Hz 2H), 7.54 (d, *J* = 8.8 Hz 2H), 7.77 (d, *J* = 8.8 Hz, 2H, 2”–H, 6”–H), 7.86–7.96 (m, 2H), 8.11 (dd, *J*_1_ = 8.4 Hz, *J*_2_ = 1.2 Hz), 8.19 (dd, *J*_1_ = 8.4 Hz, *J*_2_ = 1.2 Hz), 9.85 (br s, 1H, Ph-OH), 10.78 (br s, 1H, Ph-OH). ^13^C NMR (100 MHz, DMSO-*d_6_*) δ 115.52 (2C), 115.81 (2C), 126.86, 127.65, 128.88, 128.91, 130.33, 130.51 (2C), 131.46, 133.05 (2C), 138.65, 141.44, 151.44, 151.66, 159.02, 163.32, 192.30. Analysis calculated for C_21_H_14_N_2_O_2_**∙**1.2H_2_O: C 69.29, H 4.55, N 7.69; found C 69.02, H 4.39, N 7.51.

### 3.2. Biological Activity

#### 3.2.1. Compounds

Compounds were dissolved in DMSO at 10 mM and then diluted in culture medium.

#### 3.2.2. Cell

Human hepatoma Huh-7 cells were purchased from Bioresources Collection and Research Center, Taiwan. Huh-7-DV-Fluc cells, C6/36 cells, and DENV-2 strain 16,681 were kindly provided by Dr. Huey Nan Wu, Institute of Molecular Biology, Academia Sinica, Taipei, Taiwan, ROC.

#### 3.2.3. Cytotoxicity Assays

For cytotoxicity tests, run in parallel with antiviral assays, plates at an initial density of 5 × 10^3^ cells/well were treated with or without serial dilutions of test compounds. Cell viability was determined after 72 h at 37 °C in a humidified CO_2_ (5%) atmosphere by the (2,3-bis[2-methyloxy-4-nitro-5-sulfophenyl]-2*H*-tetrazolium-5-carboxanilide) (XTT) method [25].

#### 3.2.4. Transfection and Luciferase Activity Assay

Huh-7-DV-Fluc cells were seeded in 24-well plates at a density of 2 × 10^4^ cells per well, and treated with the 3-arylquinoxaline compounds at two concentrations (1 and 10 μM) or 0.1% DMSO as control. After three days of incubation, the luciferase activity assay was performed using the Bright-Glo Luciferase assay system (Promega, Madison, WI, USA) according to the manufacturer’s instructions. To determine the regulation of *COX-2* promoter activation during DENV-2 infection, Huh-7 cells were transfected with 1 μg of the reporter plasmid pCOX-2-Luc (BD Biosciences Clontech, Palo Alto, CA, USA) by using T-pro reagent (Ji-Feng Biotechnology CO., Ltd., Taipei, Taiwan) following the instruction of the manufacturer. After 6 h of transfection, the transfection reagents were changed with fresh medium containing **19a** at different concentrations. After three days of incubation, cell extracts were subjected to luciferase activity assay by using the Bright-Glo Luciferase Assay System (Promega) in accordance with manufacturer’s instruction. To determine exogenous gene expression, Huh-7 cells were transfected with either a vehicle vector or COX-2 expression vector pCMV-COX-2-Myc with several concentrations (0.25 and 0.5 μg) for 6 h. The transfection reagents were replaced with fresh medium containing **19a** at 1 μM. In the knockdown of *COX-2* gene expression, Huh-7 cells were transfected with either a control vector β-galactosidase (LacZ) short hairpin RNA (shRNA) or COX-2 shRNA expression vector (National RNAi Core Facility, Academia Sinica, Taipei, Taiwan) for 6 h. After three days, protein and RNA levels were analyzed by Western blotting with a specific antibody as previously described respectively [11].

#### 3.2.5. Immunoblot Analysis

Huh-7 cells were seeded in 24-well plates at a density of 5 × 10^4^ cells per well overnight and treated with indicated reagent at proper concentrations for three days. Cells were washed with cold phosphate-buffered saline (PBS) and lysed by radioimmunoprecipitation assay (RIPA) lysis buffer (50 mM Tris-HCl, pH 7.5, 150 mM NaCl, 1% Nonidet P-40, 2 mM ethylenediaminetetraacetic acid (EDTA), 1 mM ethylene glycol bis(2-aminoethyl)tetraacetic acid (EGTA), 1 mM NaVO_3_, 10 mM NaF, 1 mM dithiothreitol (DTT), 1 mM phenylmethylsulfonyl fluoride (PMSF), 25 μg/mL aprotinin, and 25 μg/mL leupeptin) and stored at −20 °C. The protein concentration was determined by the Bradford method. Then, 10-μg protein samples were separated by 10% SDS-PAGE and transferred onto a polyvinylidene fluoride (PVDF) membrane. The membrane was blocked with 5% non-fat dried milk and incubated with specific antibodies against GAPDH (1:10000, Genetex, Irvine, CA, USA) and COX-2 (1:1000; Cayman Chemical, Ann Arbor, ML, USA). Antibodies were diluted in 5% milk containing Tris-buffered saline (TBS) and 0.5% Tween. The blotting signal was developed using an enhanced chemiluminescence (ECL) detection kit (PerkinElmer, Waltham, MA, USA) and was counted by the software Quantity One (Bio-Rad, Hercules, CA, USA).

#### 3.2.6. Quantification of DENV RNA

Total cellular RNA samples were extracted after three days of compound treatment through a Total RNA Miniprep Purification Kit (GeneMark Biolab, Taiwan) following the manufacturer’s instructions, and then transcribed to complementary DNA (cDNA) with Moloney murine leukemia virus (M-MLV) reverse transcriptase (Promega, USA). The levels of DENV-2 replication and COX-2 were analyzed by RT-PCR as described previously with the following specific primers: a forward primer, 5′–AAG GTG AGA AGC AAT GC AGC–3′, and a reverse primer, 5′–CCA CTC AGG GAG TTC TCT CT–3′, targeting the DENV-2 NS5 gene 44. DENV-2 and COX-2 RNA levels were normalized to the cellular glyceraldehyde-3-phosphate dehydrogenase (GAPDH) RNA level of each sample.

#### 3.2.7. Molecular Docking Study

The crystal structure of COX-2 (PDB ID: 3LN1) was acquired from the RCSB Protein Date Bank (https://www.rcsb.org/). The 3D conformation of target compound **19a** was produced by ChemBio 3D Ultra 14.0 (PerkinElmer, Waltham, MA, USA). The molecular docking was performed by Achilles Blind Docking Server (http://bio-hpc.ucam.edu/achilles/). The “blind docking” approach was used for the docking of the small molecule to the targets, which was done without a priori knowledge of the location of the binding site by the system [26]. Visual representation of molecules was created with 3Dmol by Nicholas Rego and David Koes [27].

#### 3.2.8. Statistical Analysis

The results were expressed as means ± SD. Differences in mean values between groups were analyzed by a one-way analysis of variance (ANOVA) and Student’s *t*-test.

## 4. Conclusions

In this study, we synthesized certain 3-arylquinoxaline derivatives in order to evaluate their inhibitory activities of anti-DENV replication. Among them, compound **19a** exhibited the most potential activity against DENV replication, with an EC_50_ value of 1.29 ± 0.74 μM. By the determination of an antiviral mechanism, the results indicated that compound **19a** reduced DENV replication through COX-2 inhibition. Molecular docking results also showed that the lowest binding energy between **19a** and COX-2 enzyme was −9.10 kcal/mol. Further studies are still needed in order to improve our understanding of how these compounds elicit their anti-DENV activity.

## Figures and Tables

**Table 1:** Antiviral activities and cytotoxicities of 3-arylquinoxaline derivatives.

Compounds	DENV2		Huh-7 Cell	
	^a^ % Inhibition at 1 μM	% Inhibition at 10 μM	% viability at 20 μM	% viability at 200 μM
**3**	12.37 ± 2.67	50.14 ± 3.47	90.28 ± 2.62	21.87 ± 3.55
**4**	11.21 ± 2.31	41.24 ± 2.88	96.62 ± 8.78	41.38 ± 5.71
**15a**	2.13 ± 1.12	5.31 ± 1.73	104.71 ± 5.66	59.94 ± 8.33
**15b**	24.34 ± 2.51	48.67 ± 4.71	103.29 ± 1.79	61.42 ± 2.67
**16a**	1.97 ± 0.72	6.42 ± 2.97	108.06 ± 8.91	20.62 ± 5.37
**16b**	2.41 ± 1.48	9.46 ± 2.88	104.05 ± 4.18	72.96 ± 9.05
**17a**	2.58 ± 0.85	4.78 ± 1.93	96.35 ± 3.52	73.26 ± 6.57
**17b**	19.17 ± 3.5	22.89 ± 4.54	102.33 ± 1.98	100.73 ± 1.27
**18a**	3.41 ± 1.11	6.74 ± 1.57	102.04 ± 3.96	21.50 ± 1.60
**19a**	36.74 ± 3.71	82.71 ± 2.88	99.66 ± 1.65	85.26 ± 5.63
**19b**	35.47 ± 4.82	47.69 ± 5.41	96.43 ± 1.24	21.34 ± 1.55
**20a**	16.31 ± 3.42	58.71 ± 2.86	99.42 ± 3.31	27.12 ± 1.24
**20b**	2.75 ± 1.69	4.39 ± 1.52	95.47 ± 3.96	34.21 ± 4.97
**21a**	2.76 ± 1.55	7.98 ± 2.14	107.34 ± 2.30	79.45 ± 7.07
**21b**	42.78 ± 5.13	51.85 ± 3.52	106.46 ± 3.12	95.13 ± 4.32
**22a**	12.74 ± 3.91	19.74 ± 4.17	108.76 ± 1.36	103.05 ± 2.67
**22b**	5.19 ± 2.78	24.68 ± 1.89	103.09 ± 8.30	92.02 ± 3.08
**23a**	34.17 ± 5.12	40.78 ± 2.58	98.61 ± 5.68	87.59 ± 5.75
**23b**	16.44 ± 3.11	33.58 ± 4.21	104.26 ± 1.85	103.32 ± 1.03
**24a**	2.54 ± 1.21	23.47 ± 3.71	106.06 ± 2.12	91.94 ± 2.83
**25a**	40.44 ± 2.22	47.53 ± 3.51	101.13 ± 2.39	100.30 ± 4.59
**25b**	41.51 ± 3.25	49.74 ± 4.38	106.59 ± 3.03	99.42 ± 2.14
**26a**	1.95 ± 0.82	14.47 ± 2.55	99.39 ± 6.50	35.29 ± 4.95
**26b**	1.17 ± 0.41	19.28 ± 3.85	104.35 ± 4.22	99.11 ± 2.40
**27**	44.85 ± 3.68	48.69 ± 3.48	101.58 ± 2.84	18.43 ± 2.33
**29**	2.36 ± 1.11	4.25 ± 1.39	103.62 ± 2.91	51.91 ± 1.62
Ribavirin ^b^	10.14 ± 1.98	32.53 ± 2.30	71.37 ± 1.31	14.36 ± 2.14

DENV—dengue virus; ^a^ Values are means ± standard deviations (SD) of at three separate experiments. ^b^ Ribavirin: reference [15].

**Table 2:** Antiviral activities ((EC_50_) (μM)) ^a^ of the compounds tested.

Compounds	EC_50_^a^	CC_50_^b^	SI ^c^
**3**	12.57 ± 2.16	139.11 ± 2.32	11.07
**4**	13.16 ± 1.23	163.67 ± 2.32	12.44
**19a**	1.29 ± 0.74	>200	>155.04
**20a**	5.68 ± 0.81	>200	>35.21
**21b**	3.25 ± 0.89	>200	>61.54
Ribavirin	12.61 ± 1.17	56.31 ± 2.32	4.47

^a^ EC_50_ is the concentration of the compound resulting in a 50% inhibition in virus production. ^b^ CC_50_ is the concentration of the compound causing a 50% growth inhibition of uninfected Huh-7 cells. ^c^ SI: selectivity index. SI = CC_50_/EC_50_. Values are means ± standard deviations (SD) of at three separate experiments.

## Supplementary Materials

Supplementary materials can be found at https://www.mdpi.com/1422-0067/20/19/4786/s1.
