# Supplementary material for: Discovery of 3-Arylquinoxaline Derivatives as Potential Anti-Dengue Virus Agents
Source: Int J Mol Sci. 2019 Sep 26;20(19):4786. doi: 10.3390/ijms20194786 (PMC6801405; doi:10.3390/ijms20194786)

HCR-5761

Pulse Sequence: s2pul
Mercury-400BB "MerPlus400"
Date: Aug 9 2017
Solvent: cdcl3
Ambient temperature
Total 32 repetitions

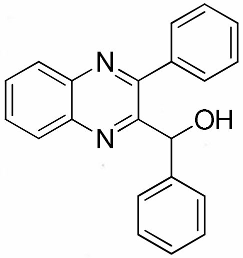

15a

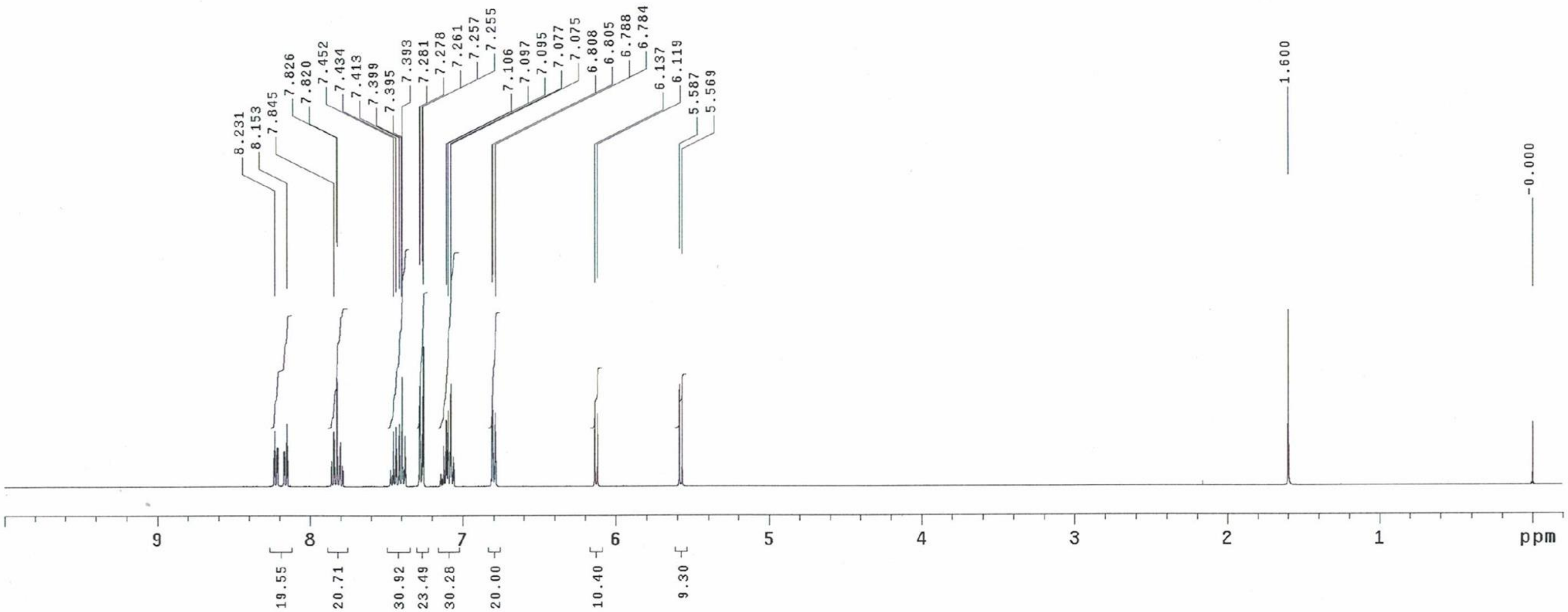

HCR-5761

Pulse Sequence: s2pul
Mercury-400BB "MerPlus400"
Date: Aug 9 2017
Solvent: cdcl3
Ambient temperature
Total 1264 repetitions

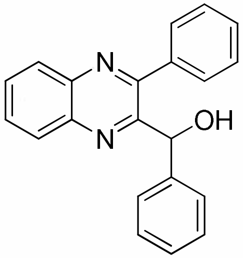

15a

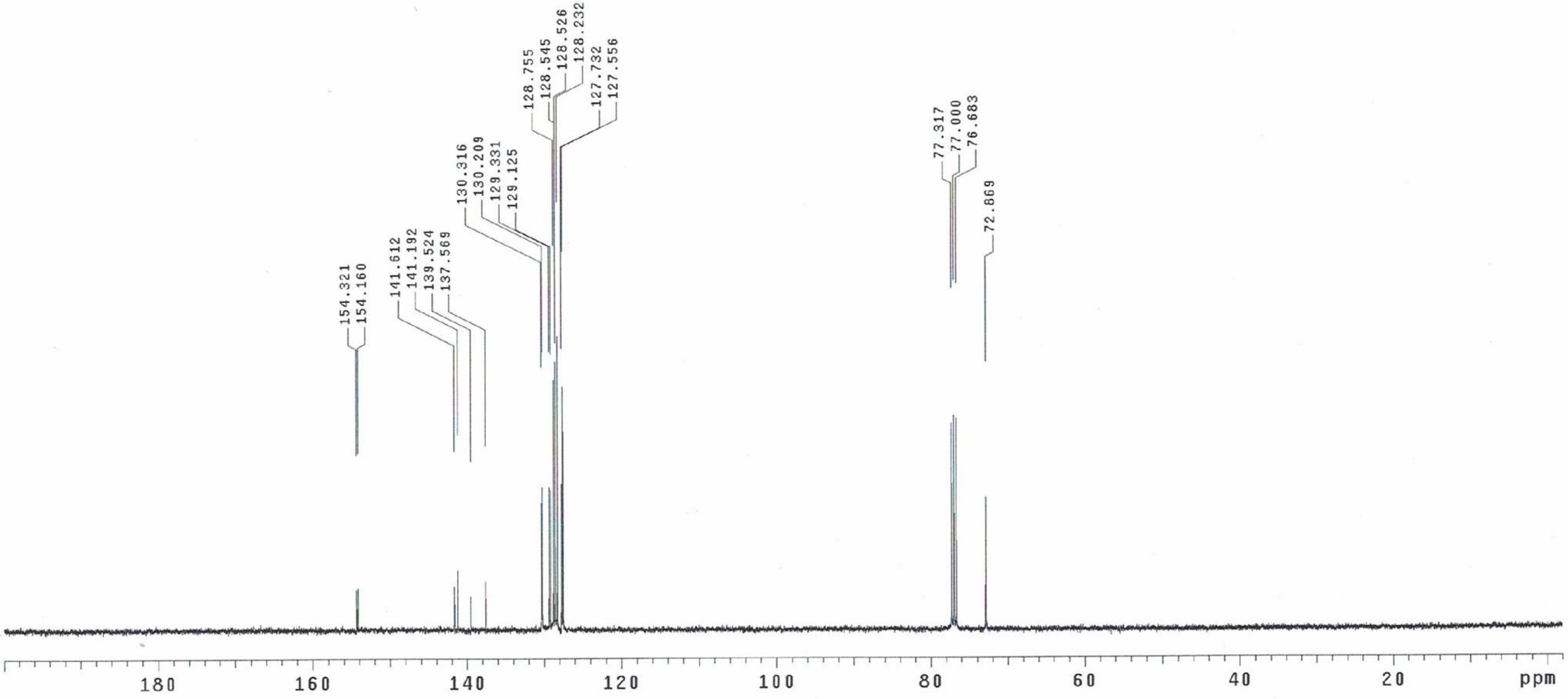

HCR-5759

Pulse Sequence: s2pul

UNITYplus-400 "unity400"
Date: Feb 22 2017
Solvent: CDCl3
Ambient temperature
Total 32 repetitions

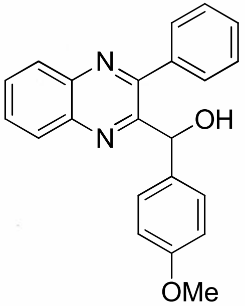

15b

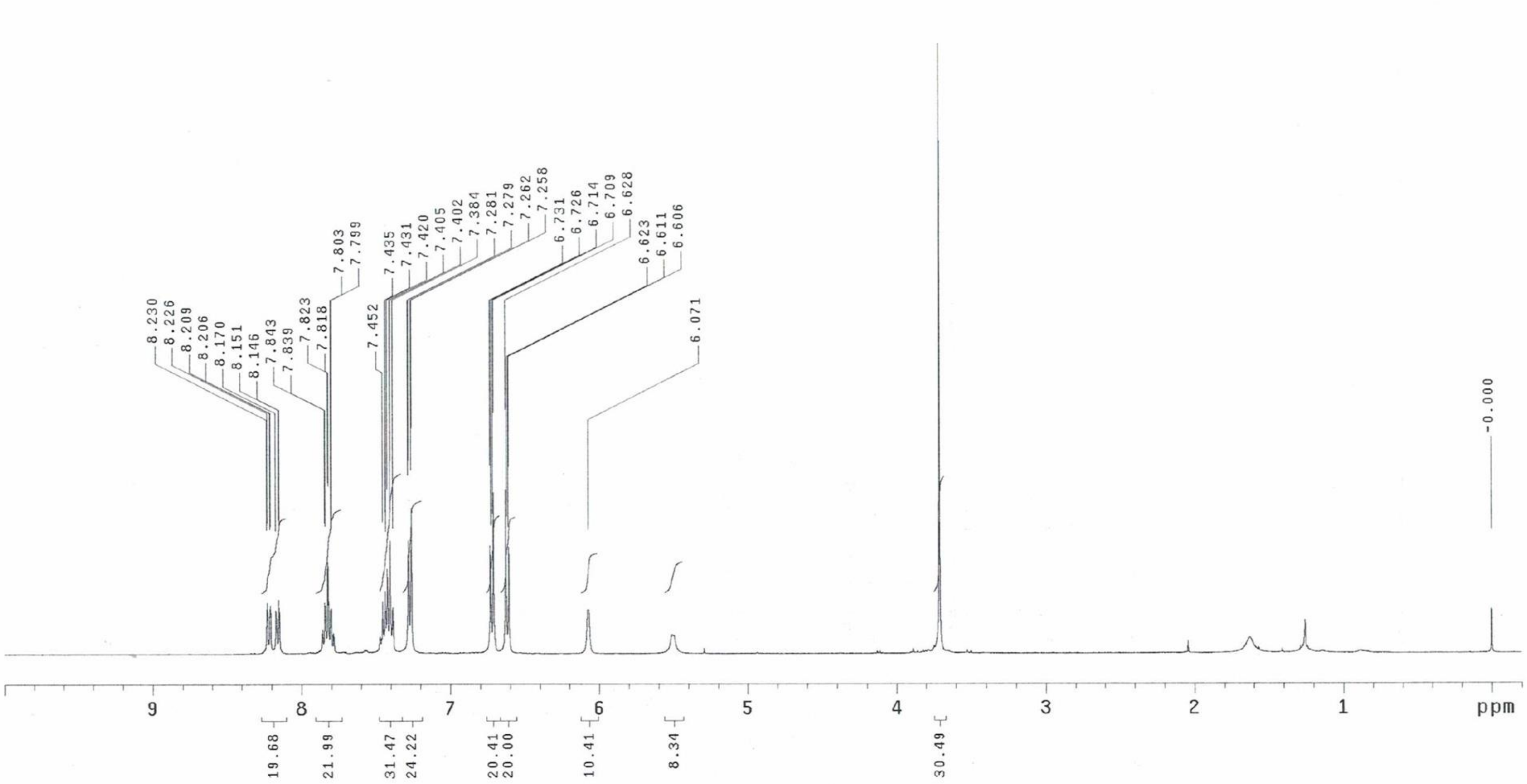

HCR-5759

Pulse Sequence: s2pul

UNITYplus-400 "unity400"
Date: Feb 22 2017
Solvent: CDCl3
Ambient temperature
Total 3792 repetitions

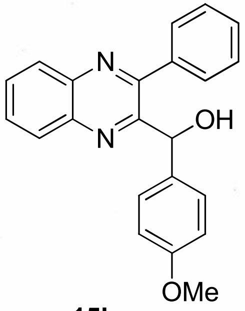

15b

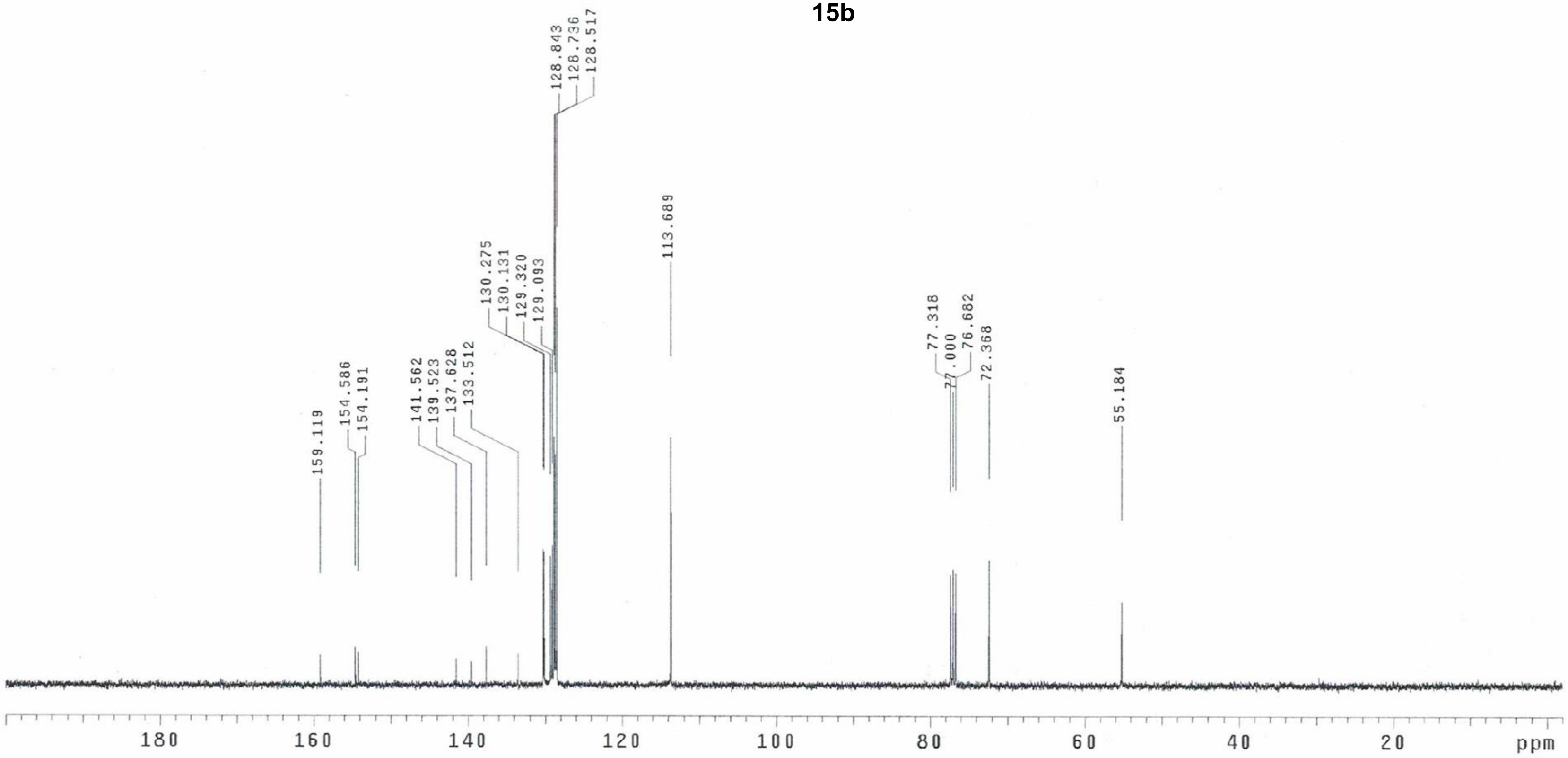

HCR-5773

Pulse Sequence: s2pul
Mercury-400BB "MerPlus400"
Date: Oct 3 2017
Solvent: cdcl3
Ambient temperature
Total 32 repetitions

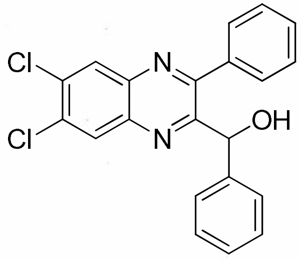

16a

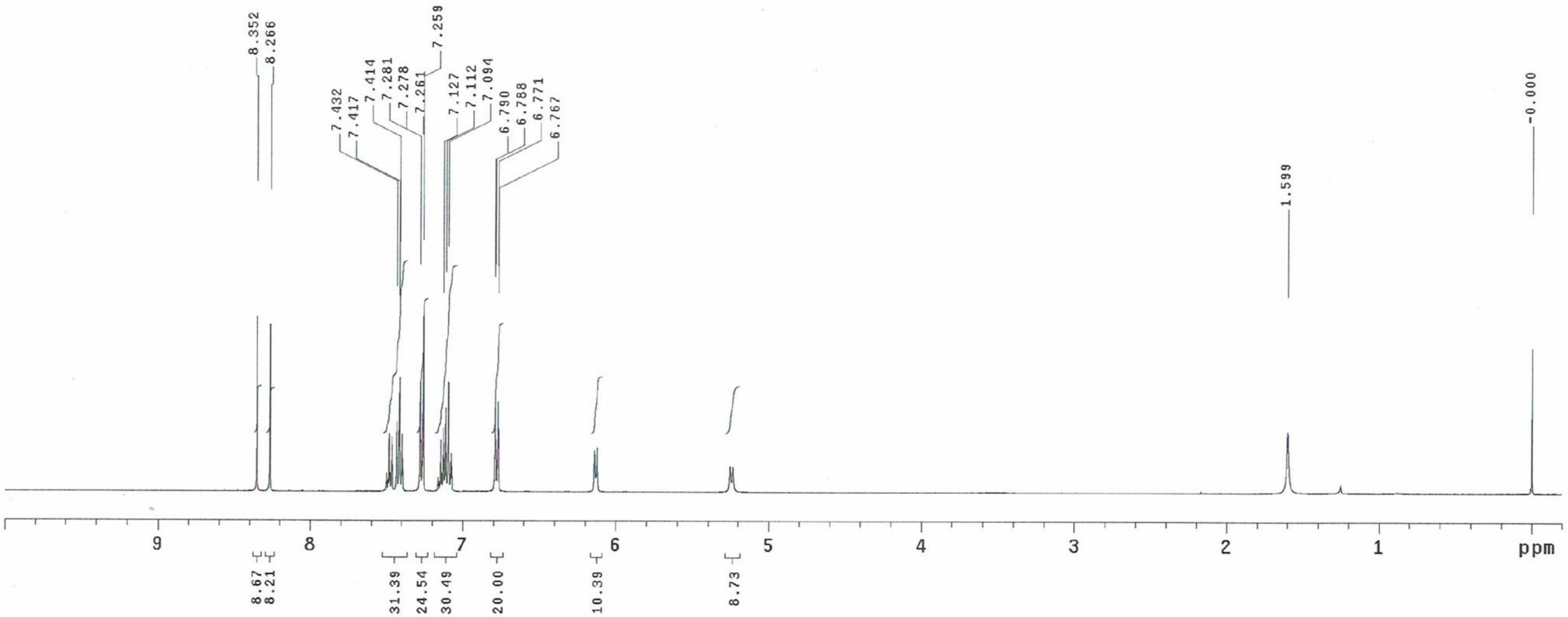

HCR-5773

Pulse Sequence: s2pul
Mercury-400BB "MerPlus400"
Date: Oct 3 2017
Solvent: cdcl3
Ambient temperature
Total 2048 repetitions

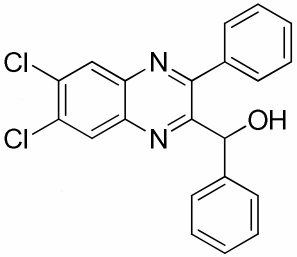

16a

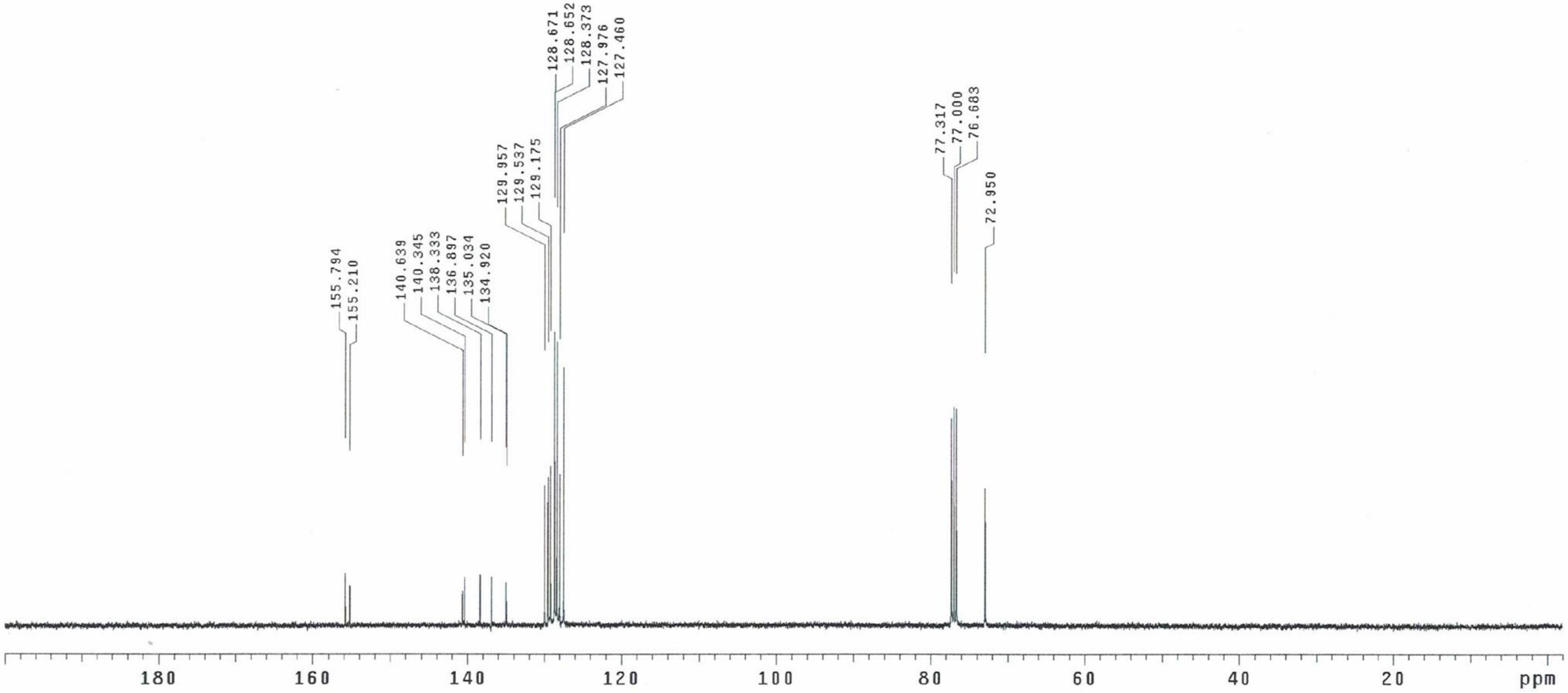

HCR-5781

Pulse Sequence: s2pul
UNITYplus-400 "unity400"
Date: Feb 5 2018
Solvent: CDCl3
Ambient temperature
Total 32 repetitions

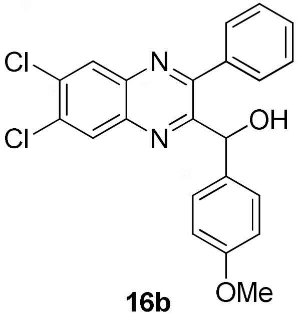

16b

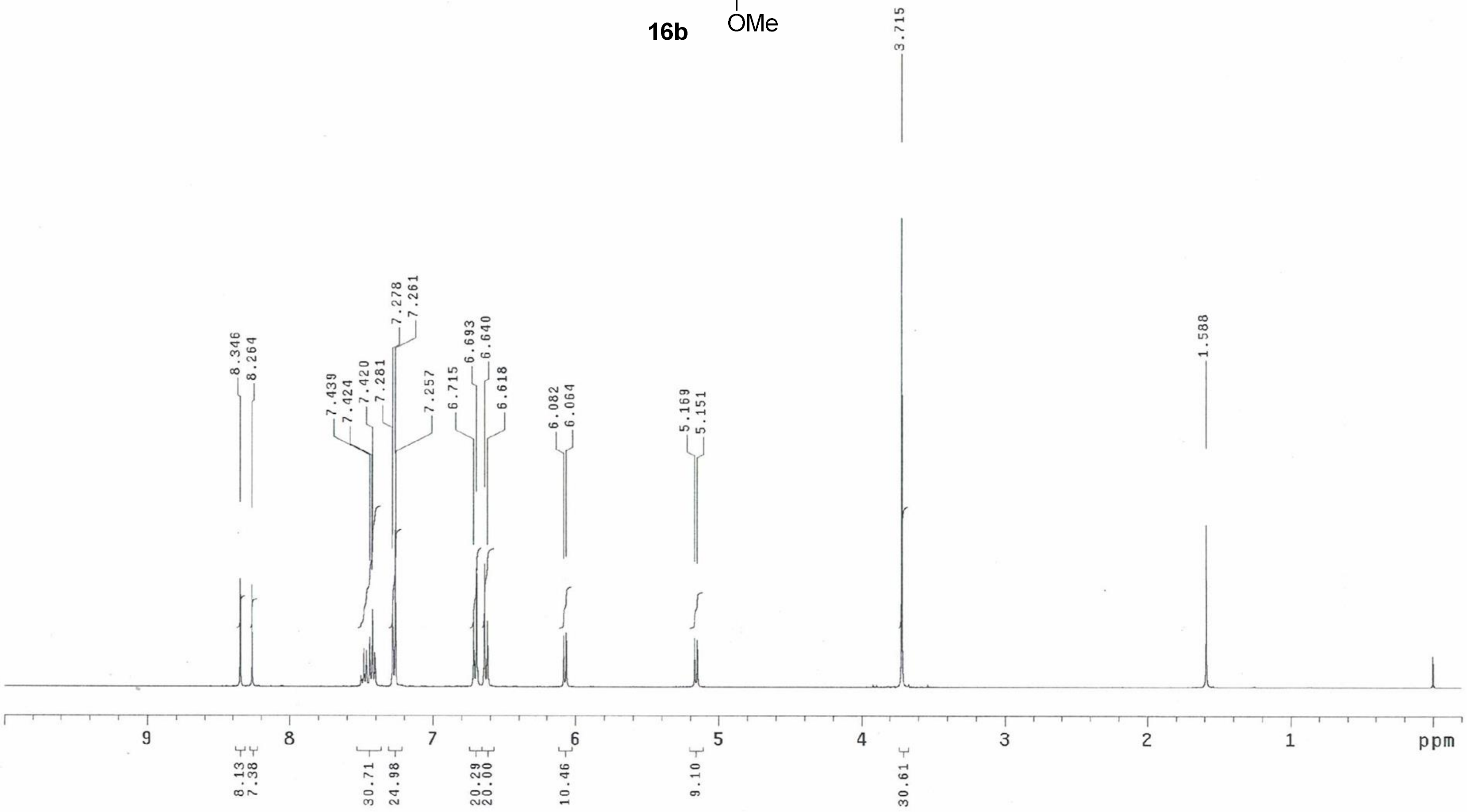

HCR-5781

Pulse Sequence: s2pul
UNITYplus-400 "unity400"
Date: Feb 5 2018
Solvent: CDCl3
Ambient temperature
Total 3872 repetitions

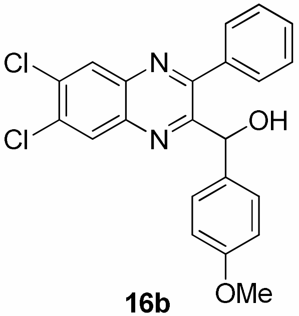

16b

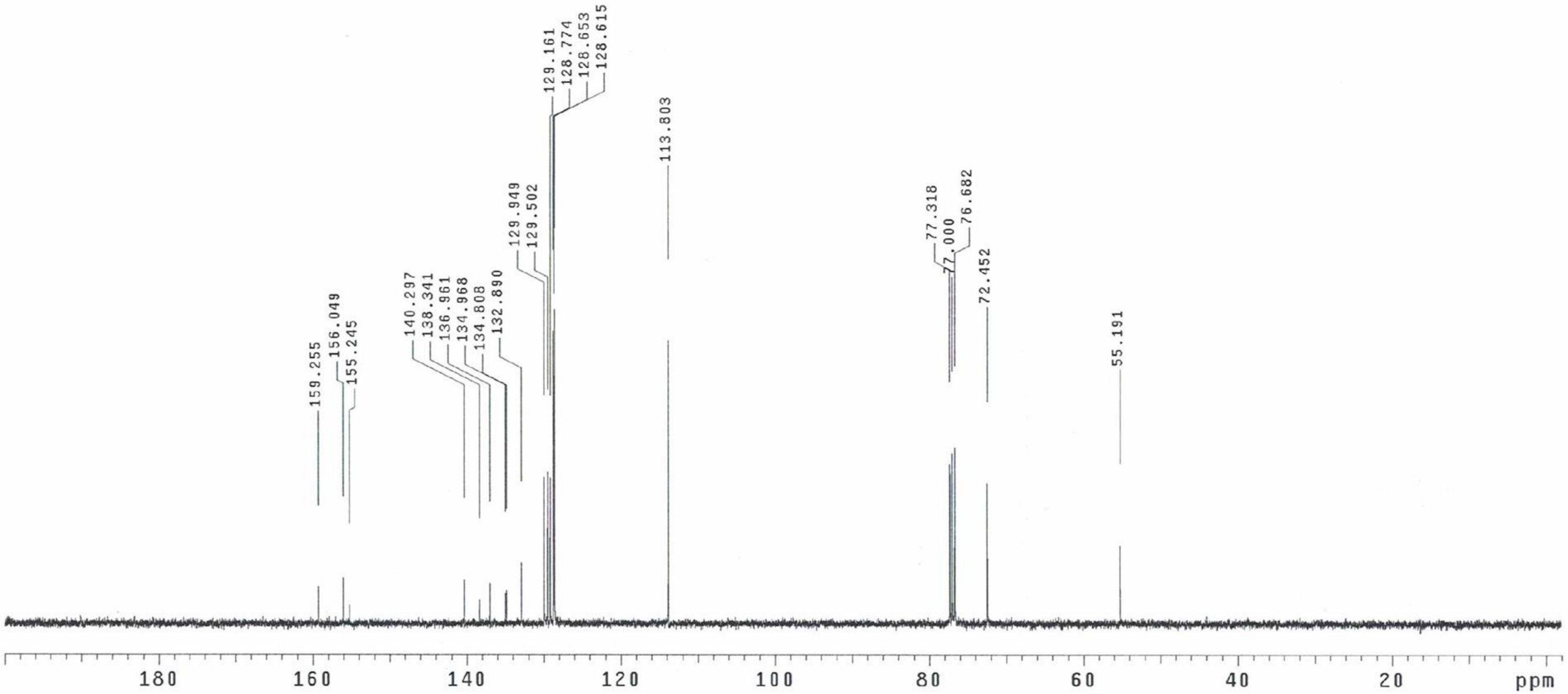

HCR-5769

Pulse Sequence: s2pul
UNITYplus-400 "unity400"
Date: Sep 20 2017
Solvent: CDCl3
Ambient temperature
Total 32 repetitions

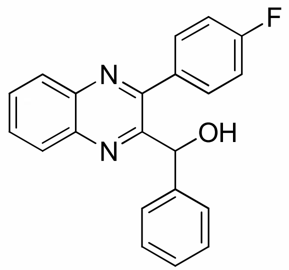

17a

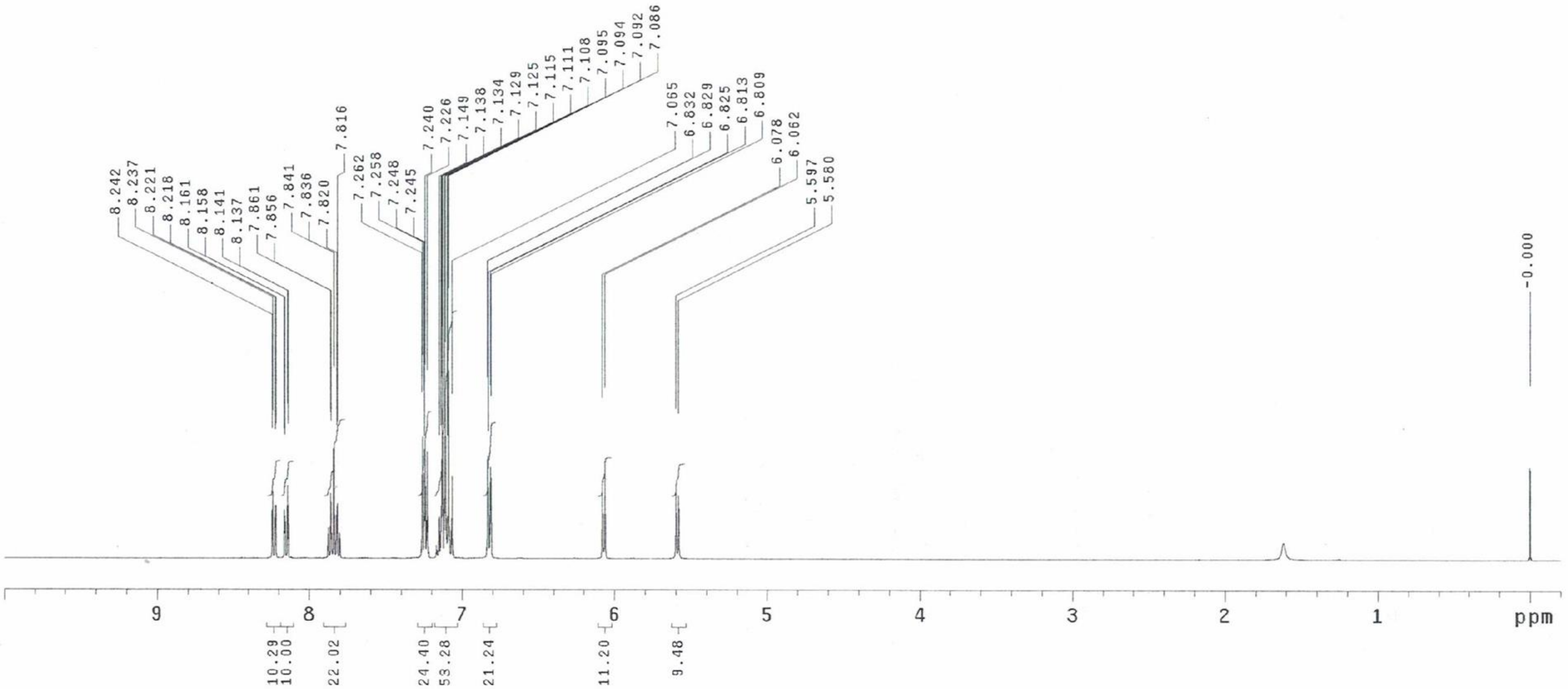

HCR-5769

Pulse Sequence: s2pul

UNITYplus-400 "unity400"
Date: Sep 20 2017
Solvent: DMSO
Ambient temperature
Total 4752 repetitions

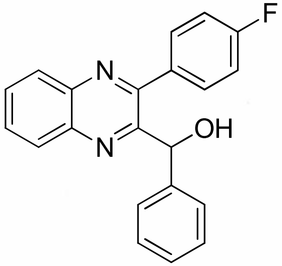

17a

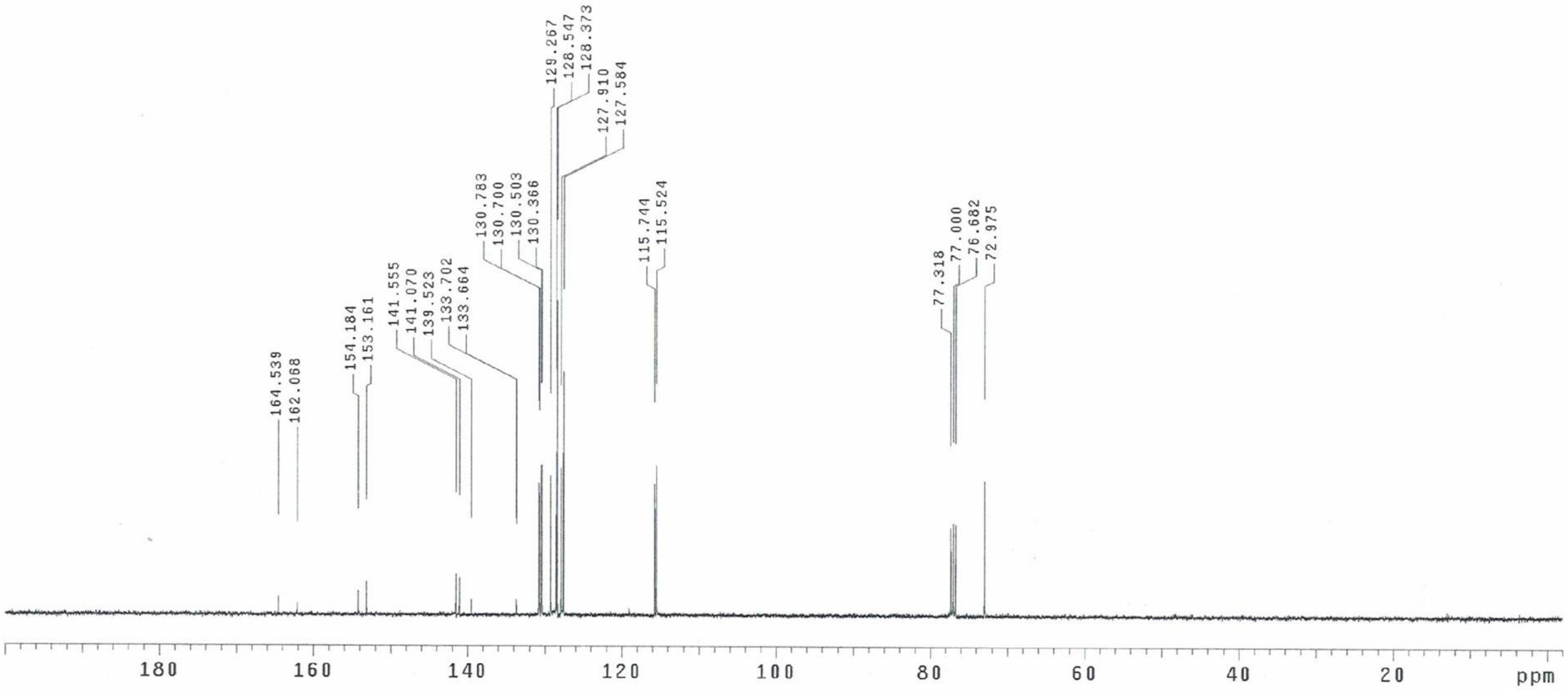

HCR-5779

Pulse Sequence: s2pul
Mercury-400BB "MerPlus400"
Date: Dec 14 2017
Solvent: cdcl3
Ambient temperature
Total 32 repetitions

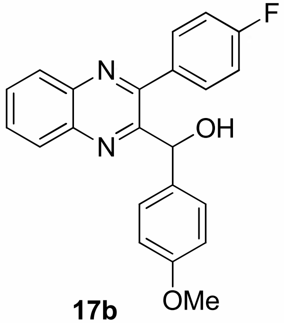

17b

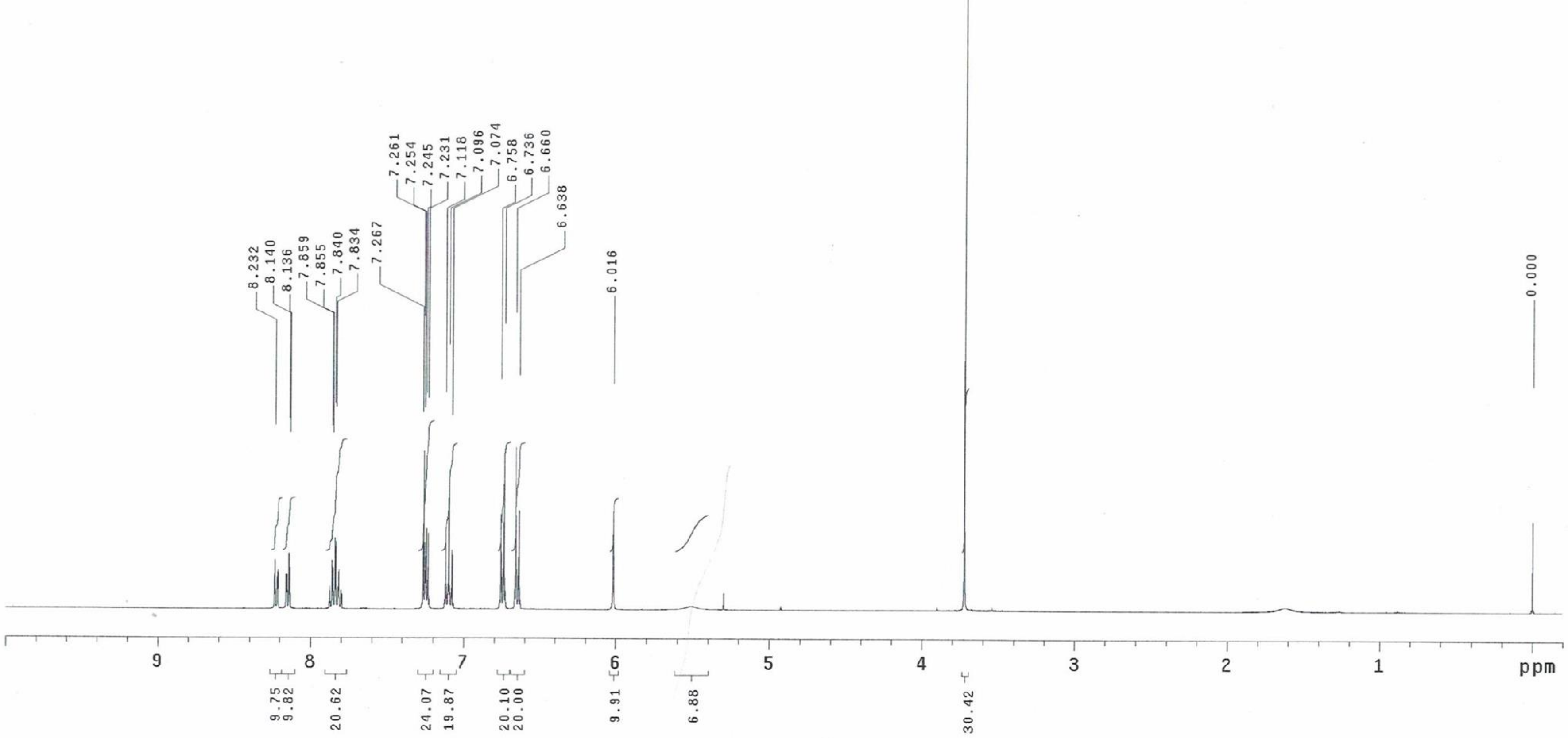

HCR-5779

Pulse Sequence: s2pul
Mercury-400BB "MerPlus400"
Date: Dec 14 2017
Solvent: cdcl3
Ambient temperature
Total 10944 repetitions

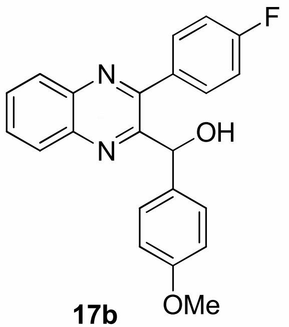

17b

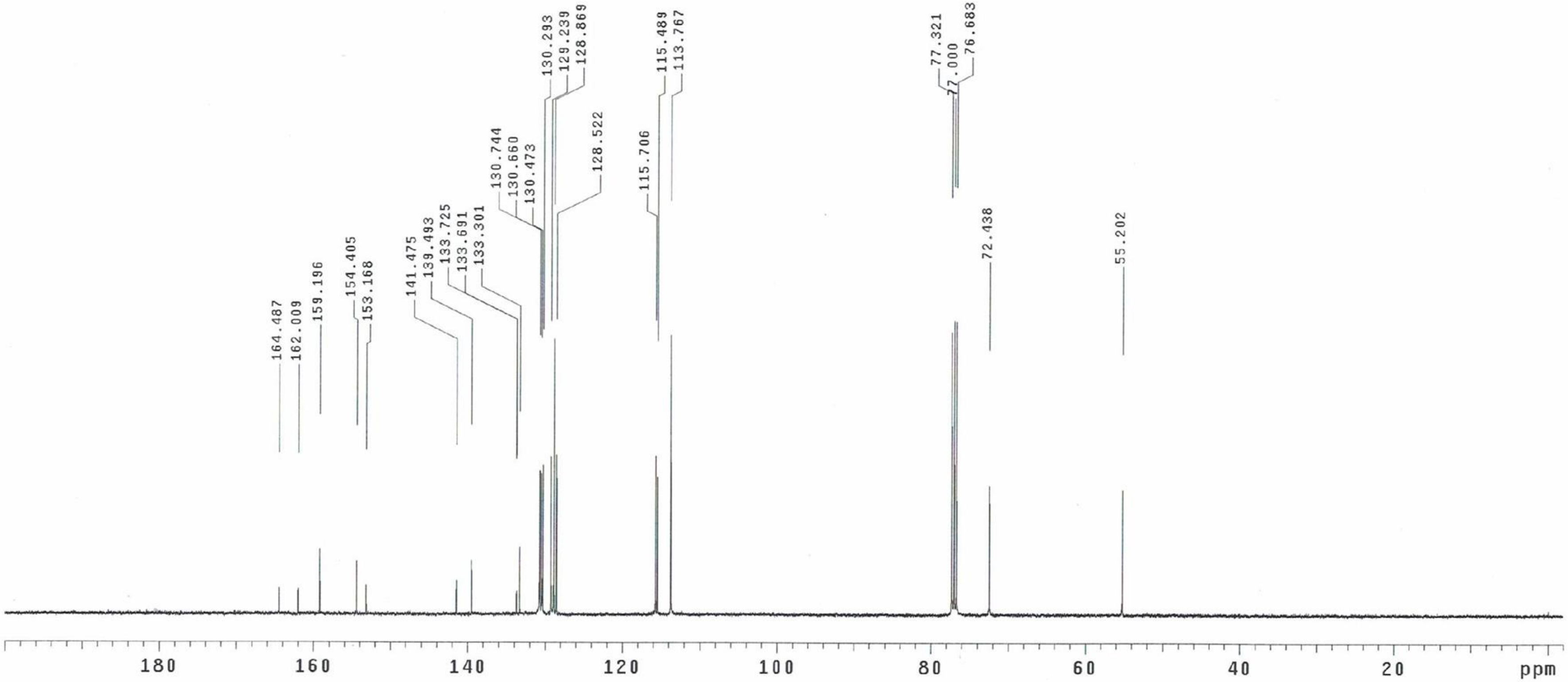

HCR-6051

Pulse Sequence: s2pul
Mercury-400BB "MerPlus400"
Date: Jun 6 2018
Solvent: cdcl3
Ambient temperature
Total 32 repetitions

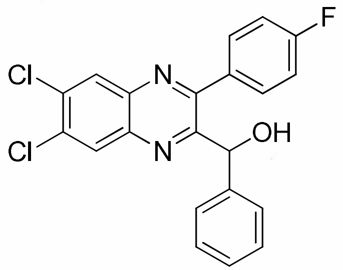

18a

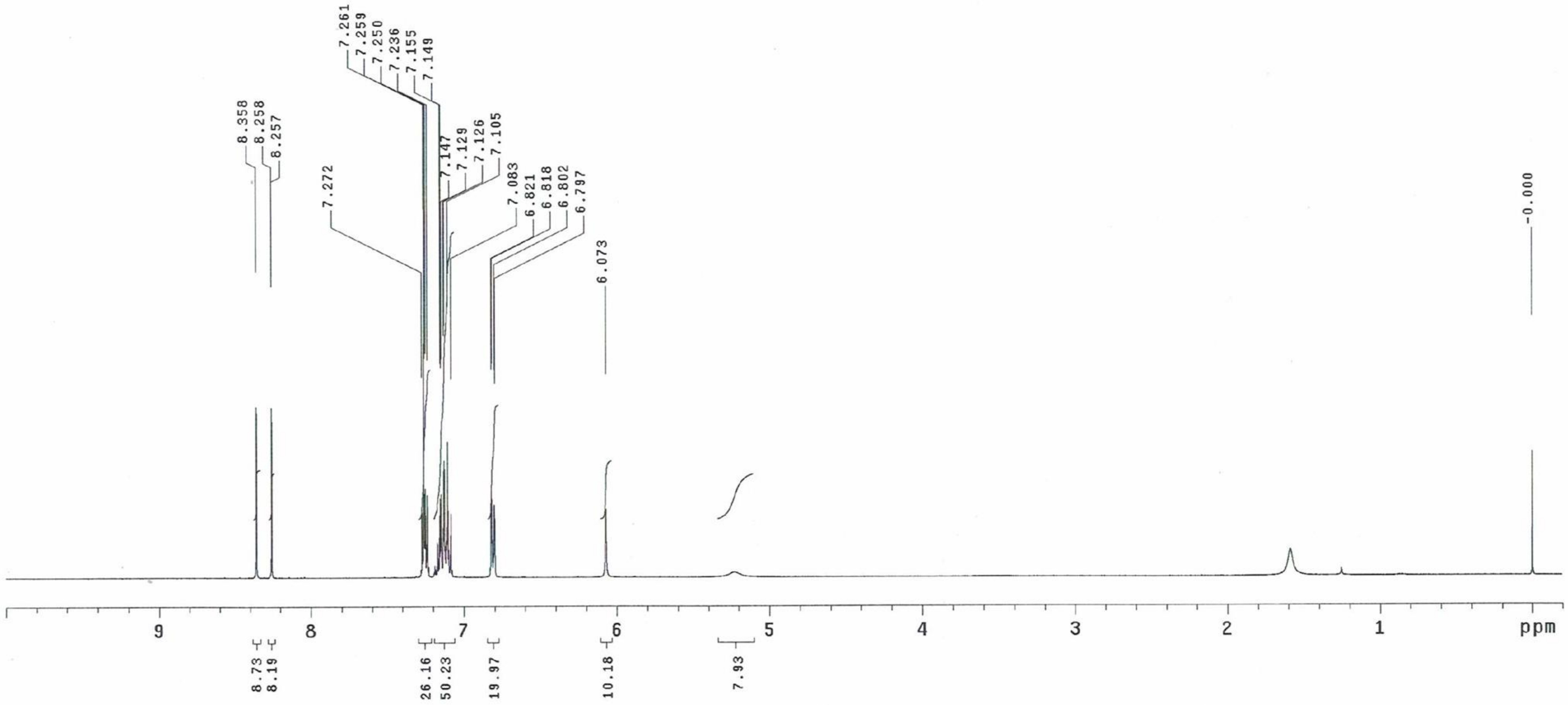

HCR-6051

Pulse Sequence: s2pul
Mercury-400BB "MerPlus400"
Date: Jun 6 2018
Solvent: cdcl3
Ambient temperature
Total 1776 repetitions

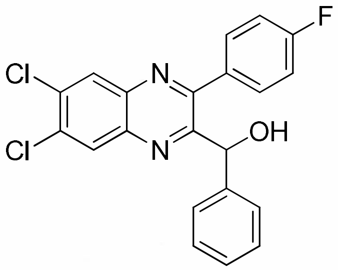

18a

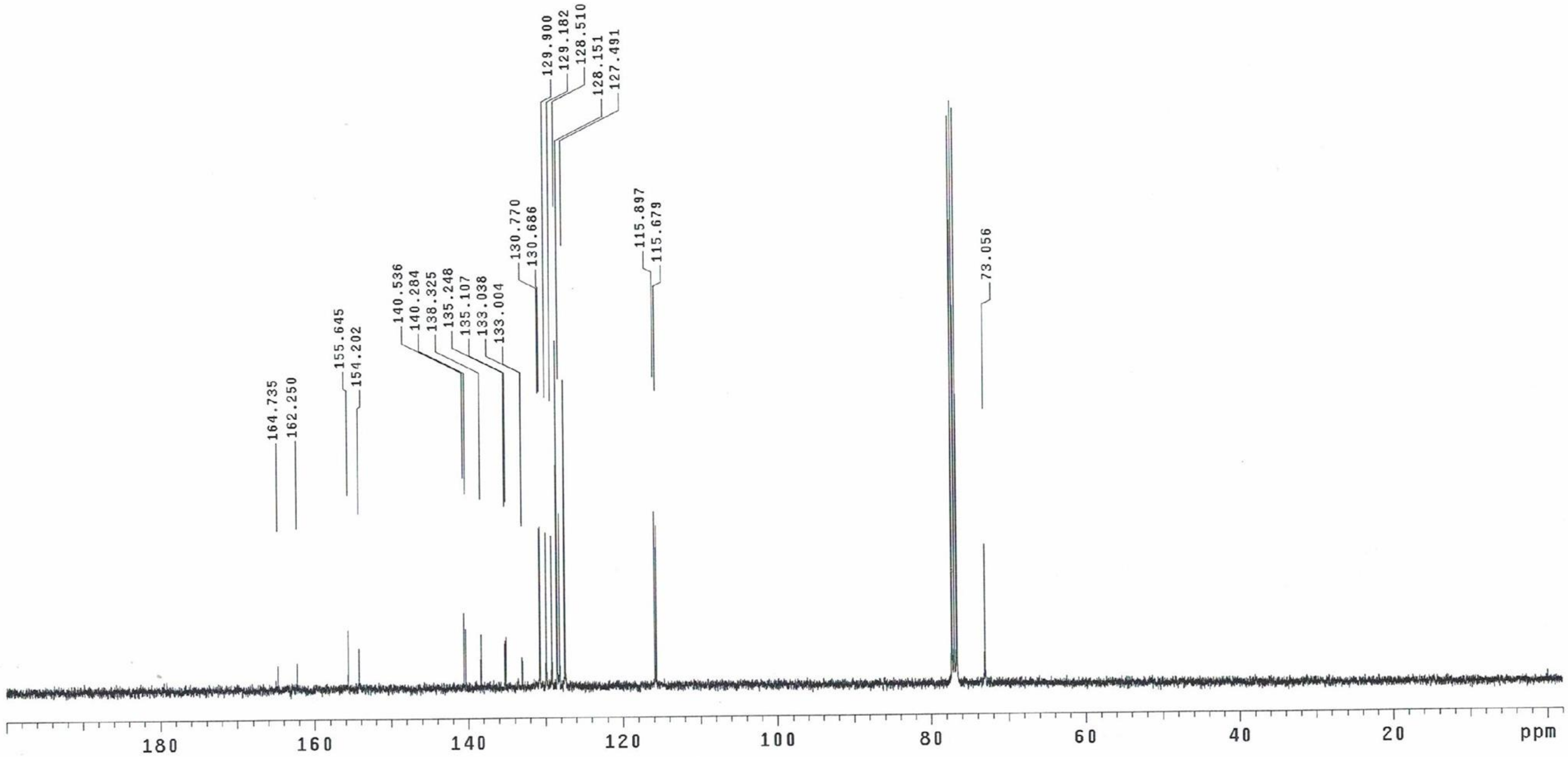

HCR-5765

Pulse Sequence: s2pul
Mercury-400BB "MerPlus400"
Date: Aug 9 2017
Solvent: cdcl3
Ambient temperature
Total 32 repetitions

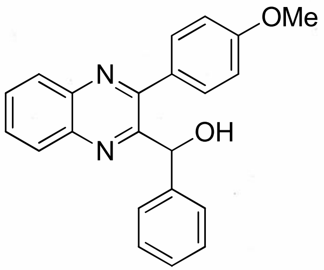

19a

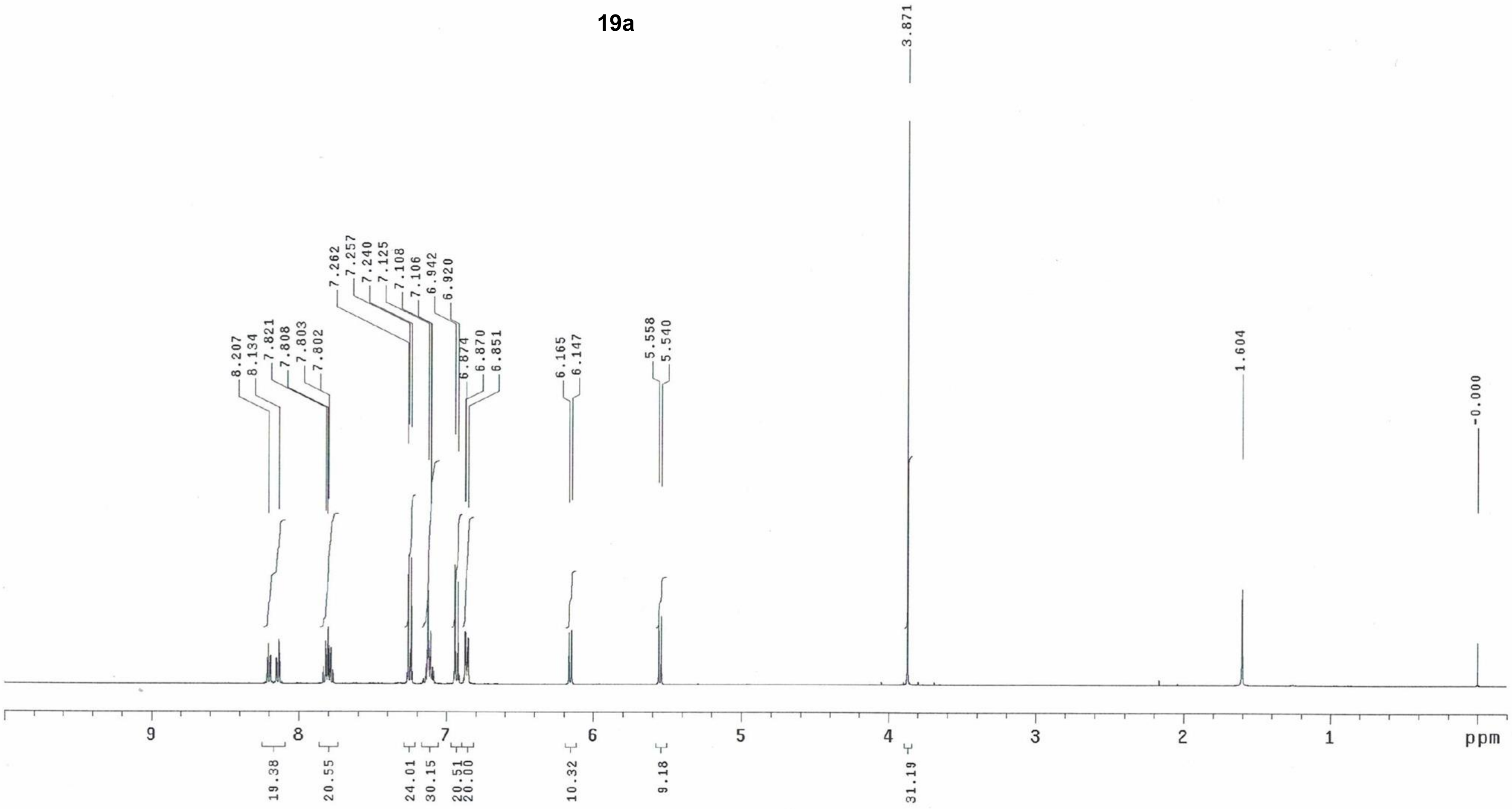

HCR-5765

Pulse Sequence: s2pul
Mercury-400BB "MerPlus400"
Date: Aug 9 2017
Solvent: cdcl3
Ambient temperature
Total 4224 repetitions

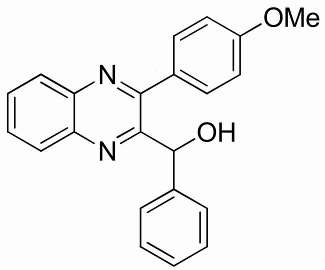

19a

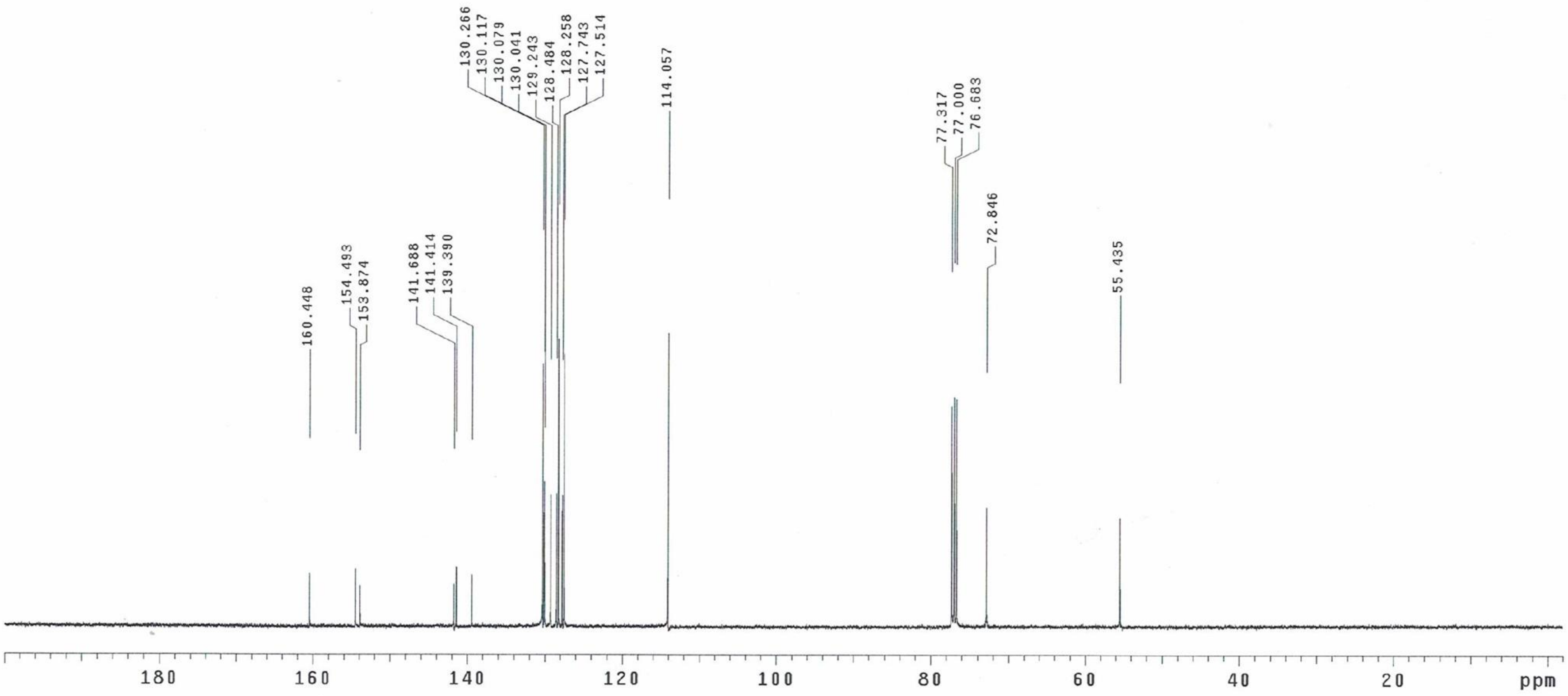

HCR-5767

Pulse Sequence: s2pul
Mercury-400BB "MerPlus400"
Date: Aug 9 2017
Solvent: cdcl3
Ambient temperature
Total 32 repetitions

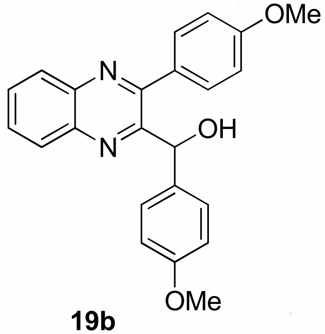

19b

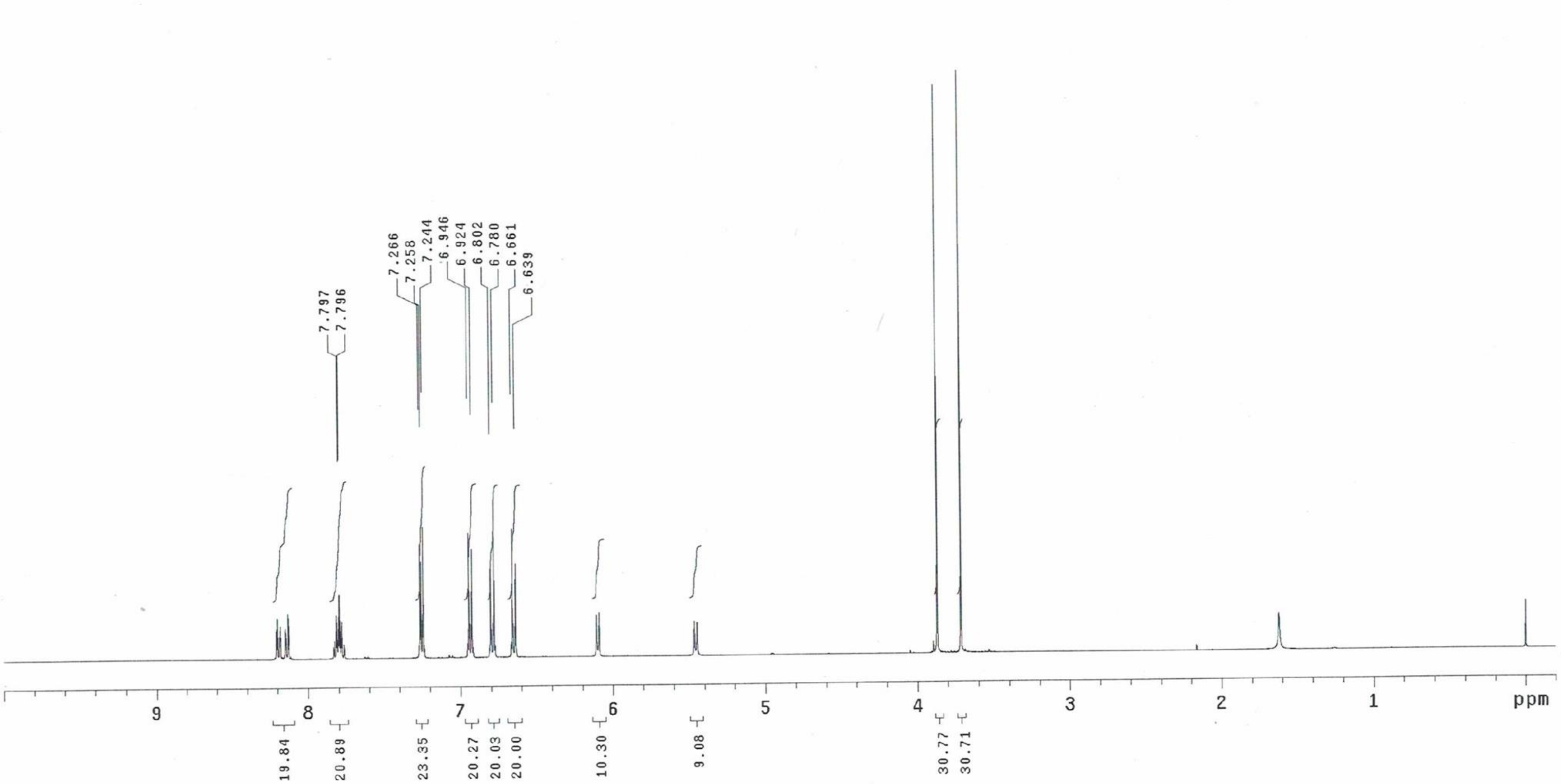

HCR-5767

Pulse Sequence: s2pul
Mercury-400BB "MerPlus400"
Date: Aug 9 2017
Solvent: cdcl3
Ambient temperature
Total 1760 repetitions

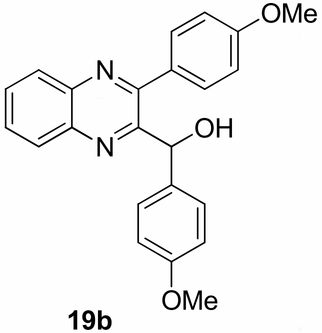

19b

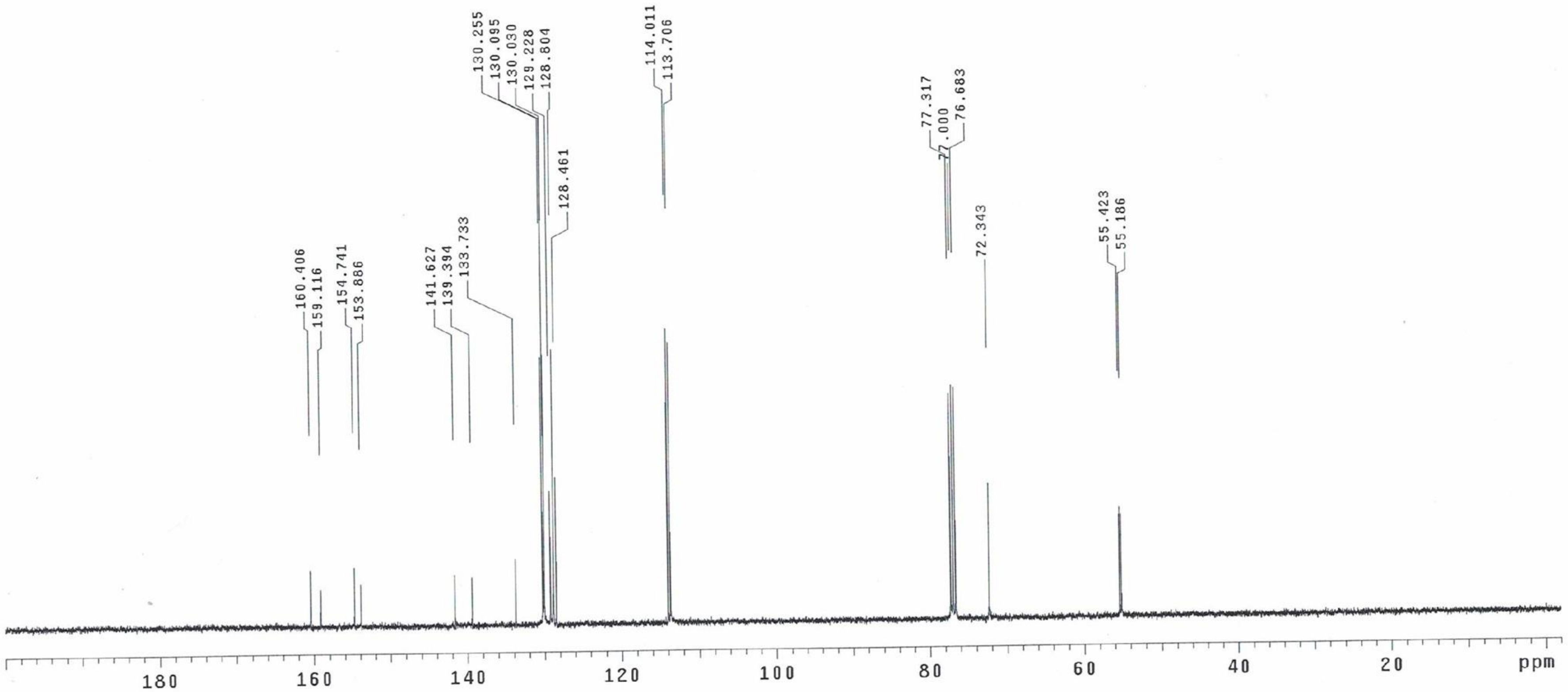

HCR-5783

Pulse Sequence: s2pul
UNITYplus-400 "unity400"
Date: Feb 5 2018
Solvent: CDCl3
Ambient temperature
Total 32 repetitions

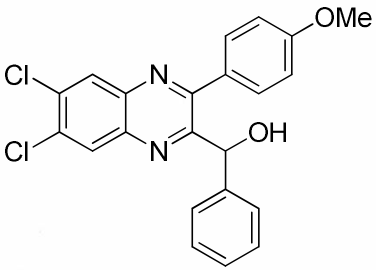

20a

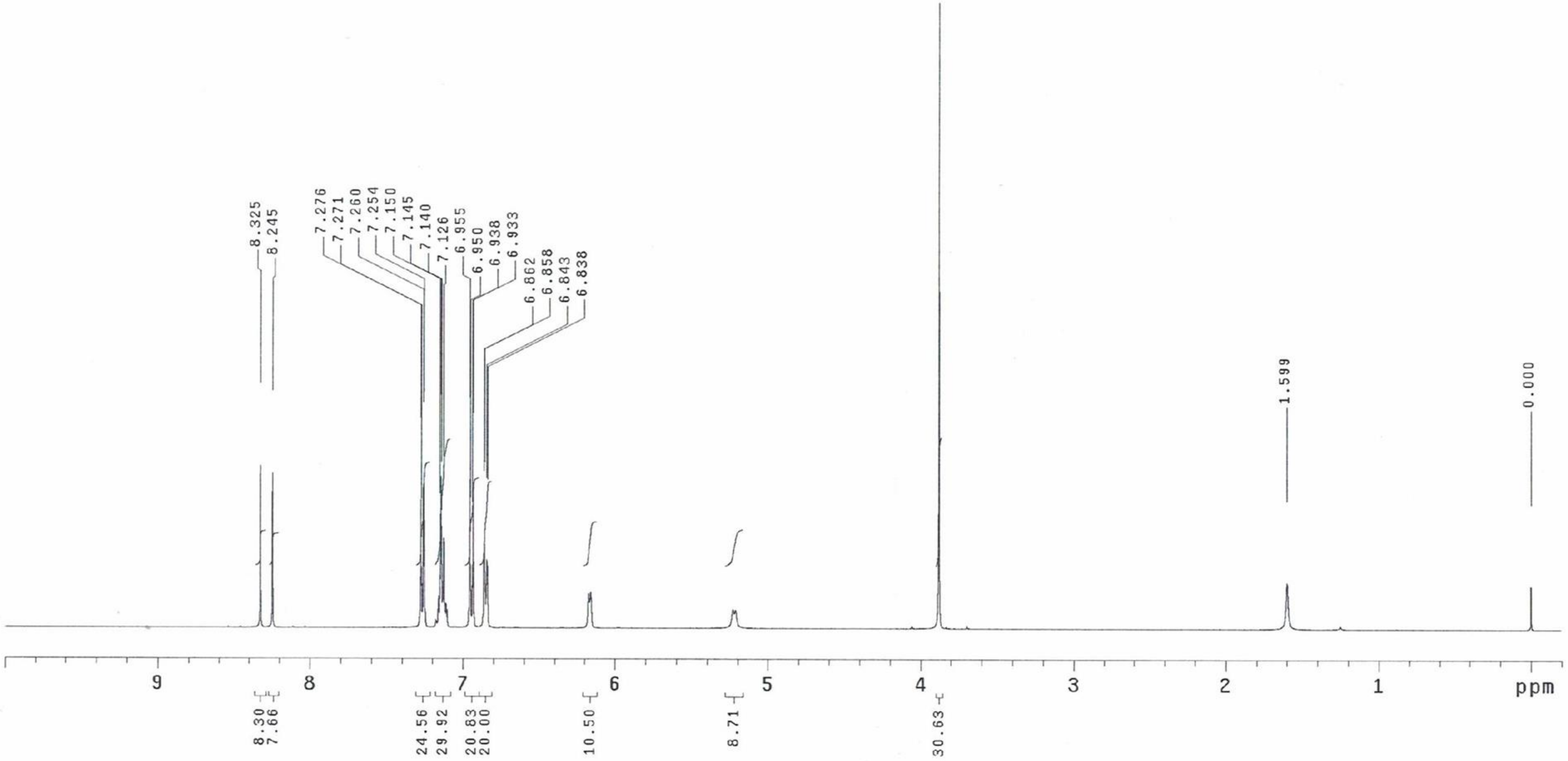

HCR-5783

Pulse Sequence: s2pul
UNITYplus-400 "unity400"
Date: Feb 5 2018
Solvent: CDCl3
Ambient temperature
Total 2320 repetitions

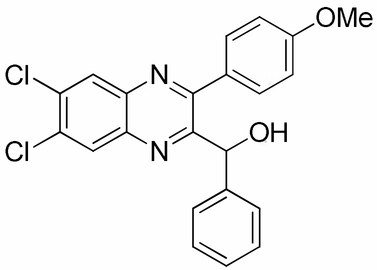

20a

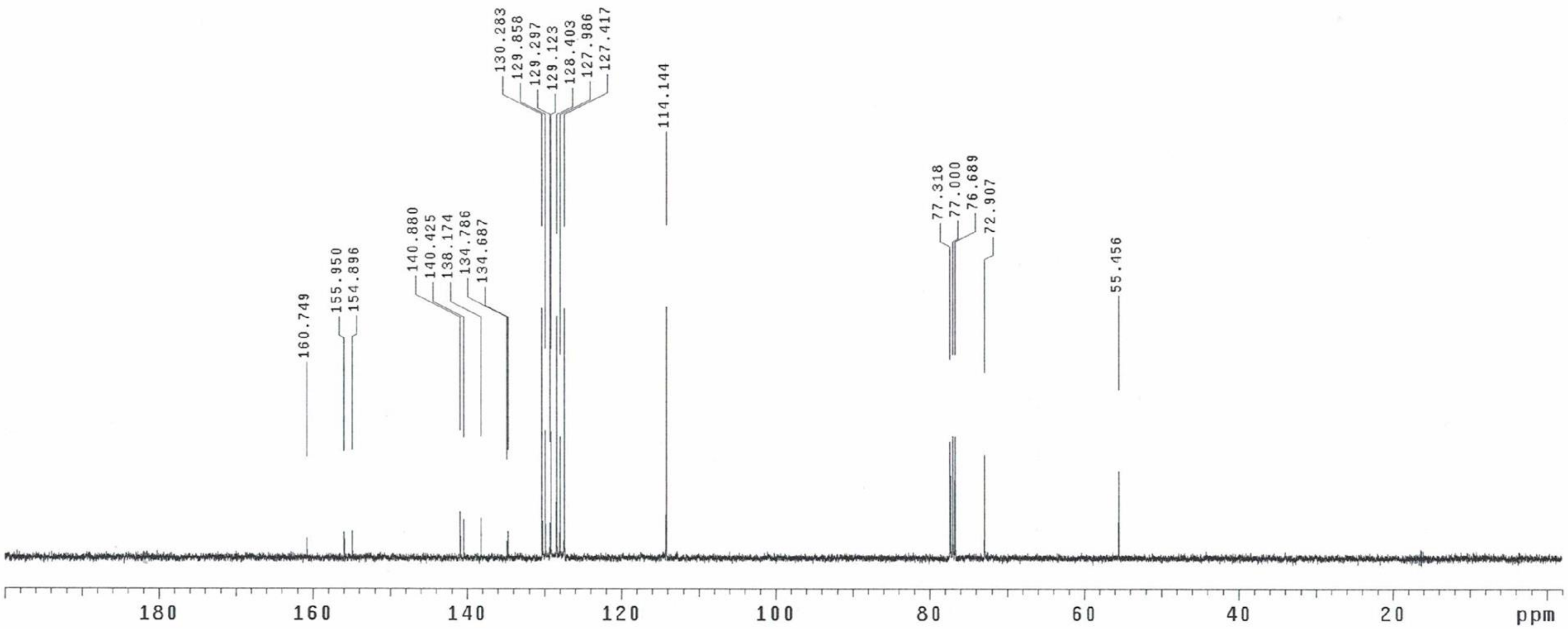

HCR-5785

Pulse Sequence: s2pul
UNITYplus-400 "unity400"
Date: Feb 23 2018
Solvent: CDCl3
Ambient temperature
Total 32 repetitions

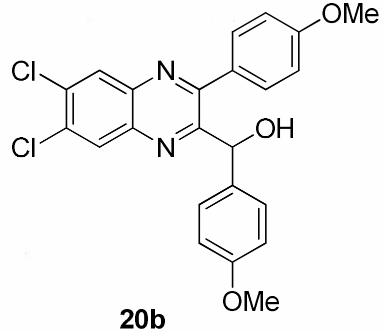

20b

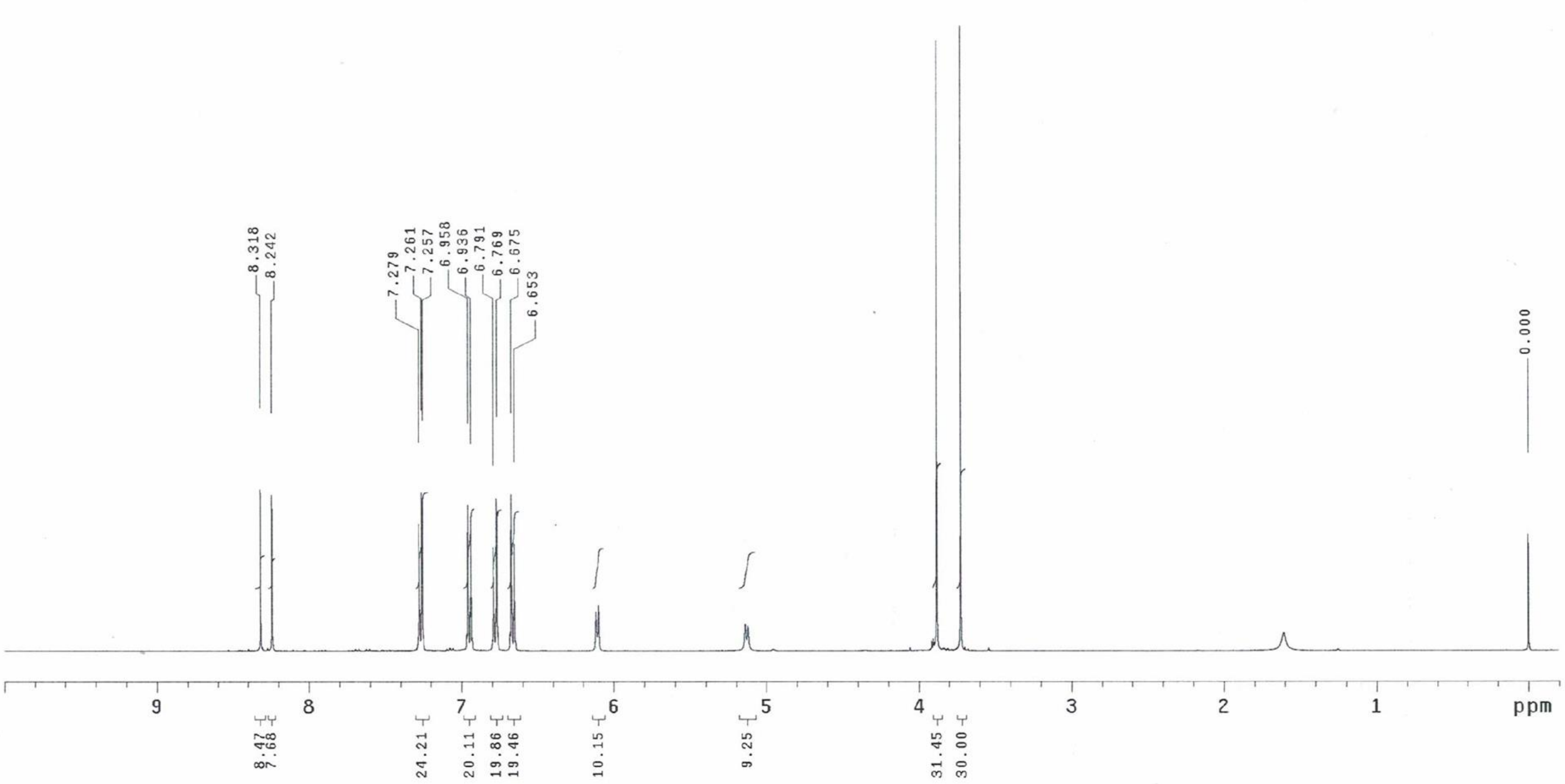

HCR-5785

Pulse Sequence: s2pul
UNITYplus-400 "unity400"
Date: Feb 23 2018
Solvent: CDCl3
Ambient temperature
Total 3504 repetitions

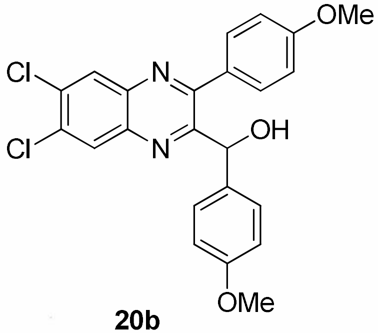

20b

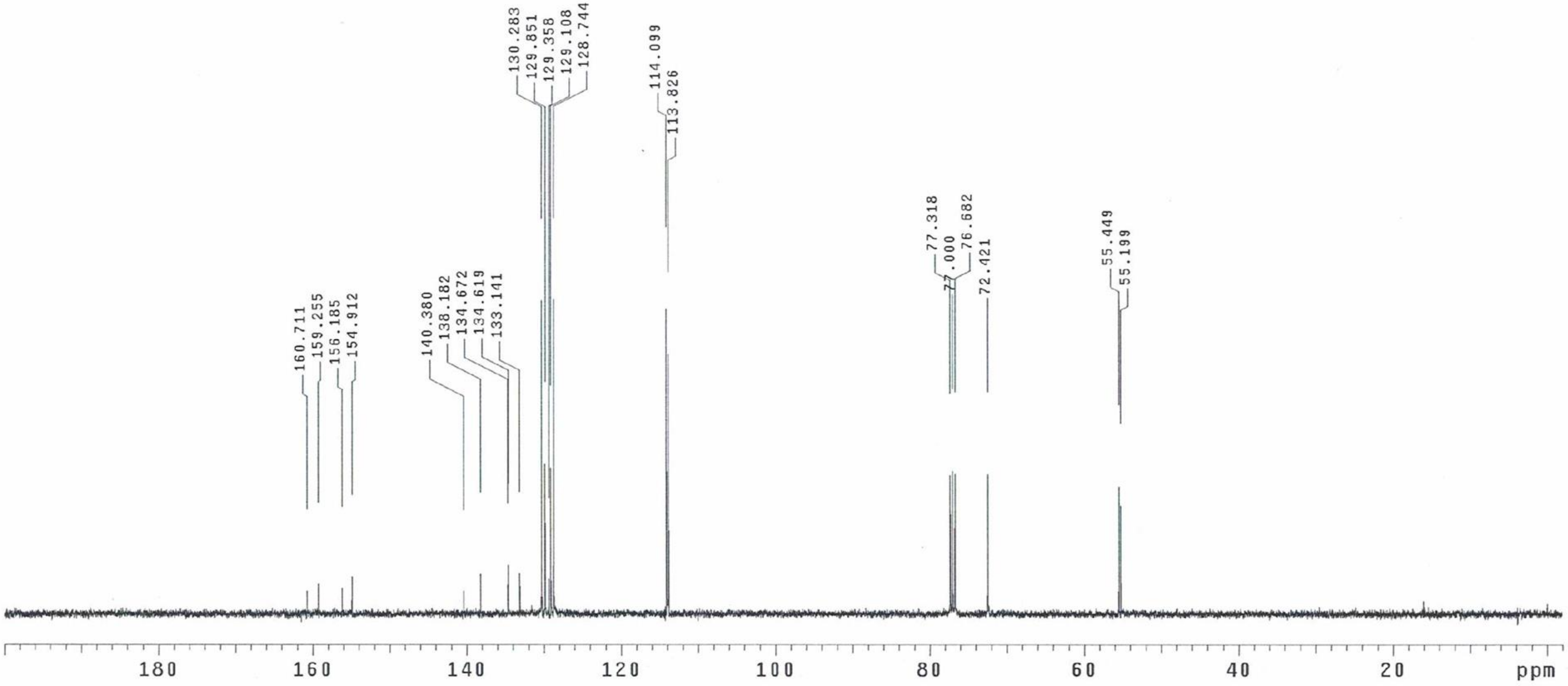

HCR-5762

Pulse Sequence: s2pul
Mercury-400BB "MerPlus400"
Date: Aug 9 2017
Solvent: cdcl3
Ambient temperature
Total 32 repetitions

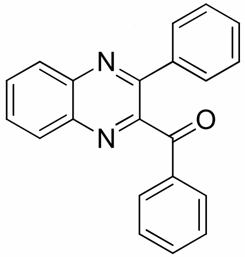

21a

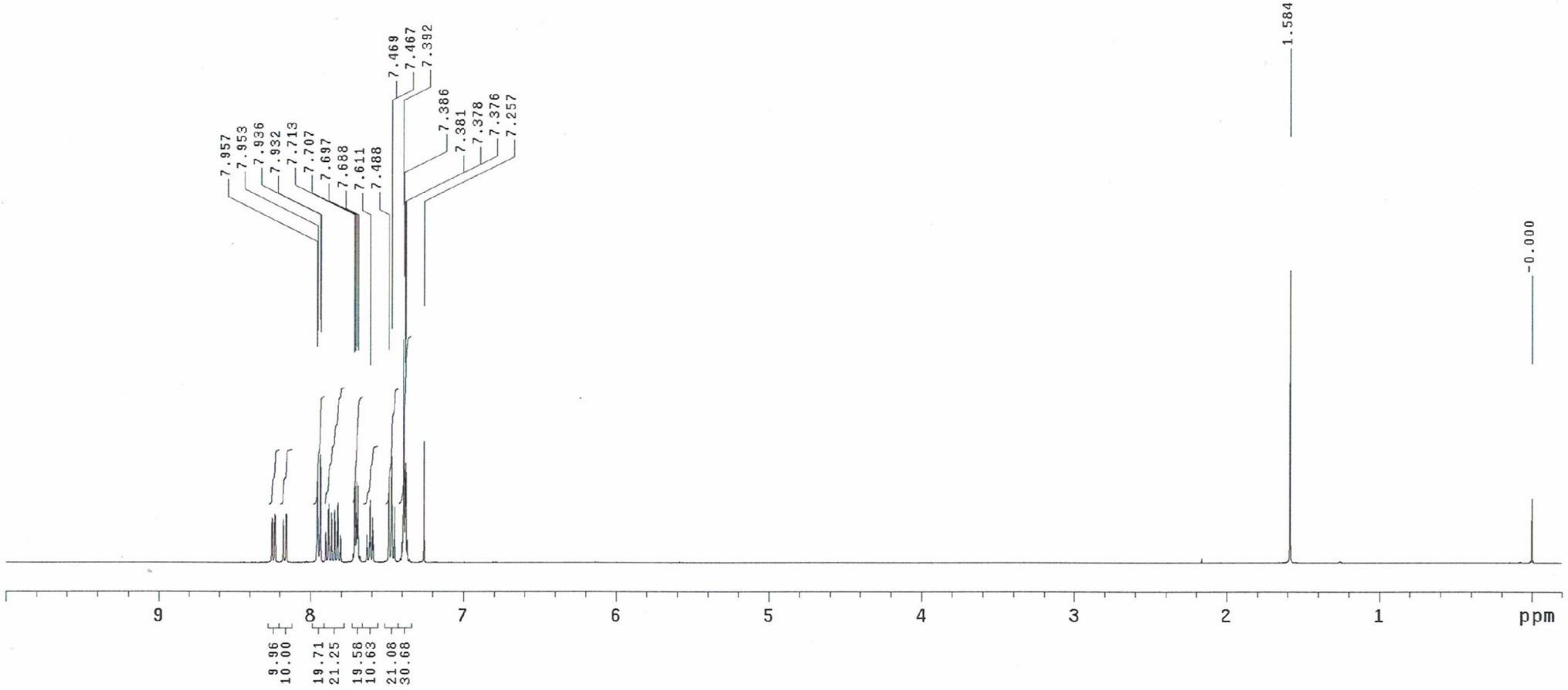

HCR-5762

Pulse Sequence: s2pul
Mercury-400BB "MerPlus400"
Date: Aug 9 2017
Solvent: cdcl3
Ambient temperature
Total 1968 repetitions

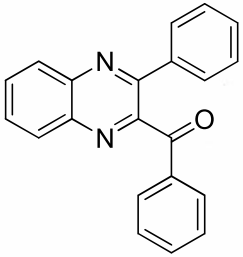

21a

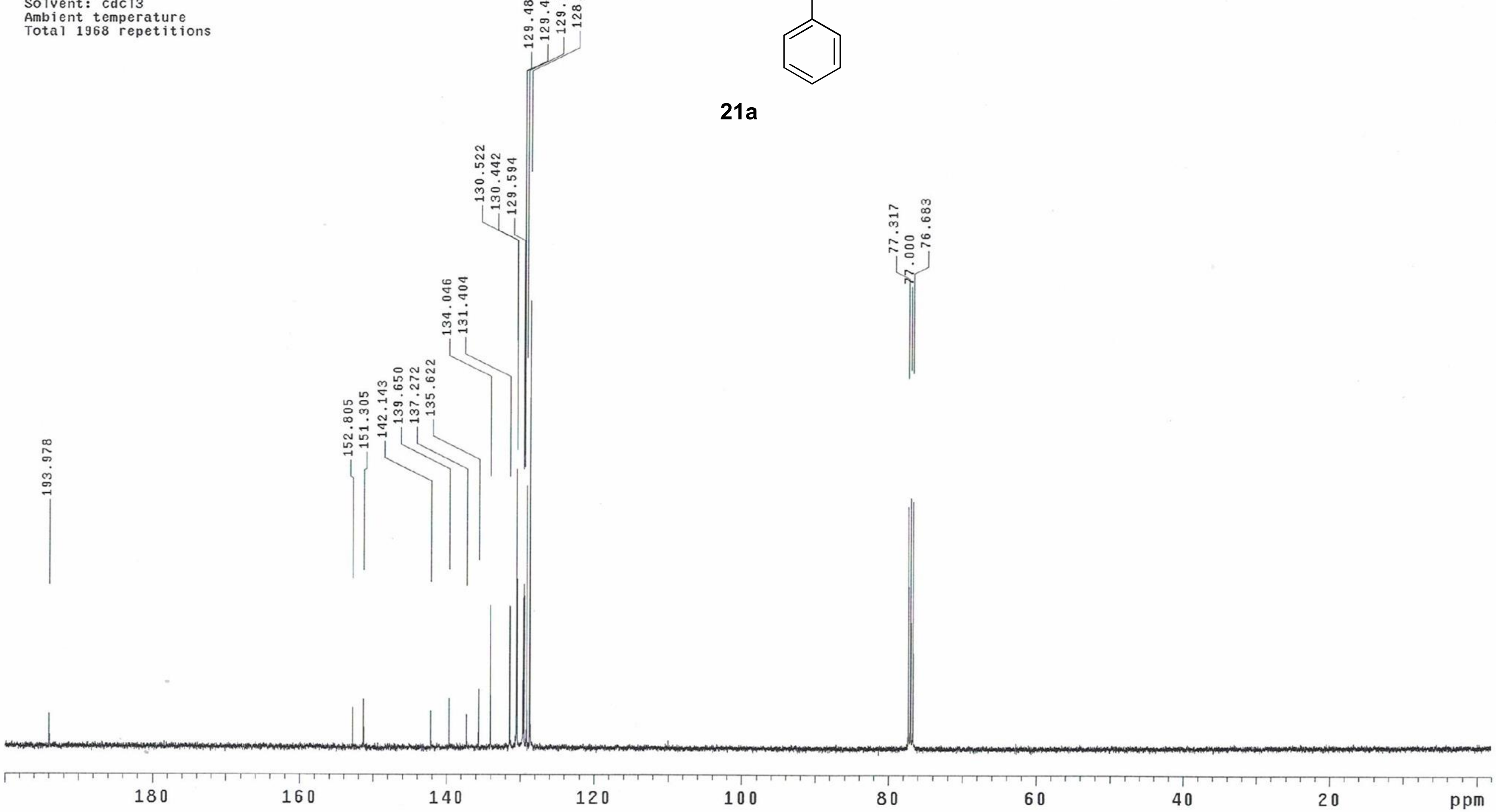

HCR-5760

Pulse Sequence: s2pul

UNITYplus-400 "unity400"
Date: Feb 20 2017
Solvent: CDCl3
Ambient temperature
Total 32 repetitions

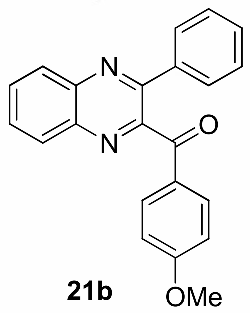

21b

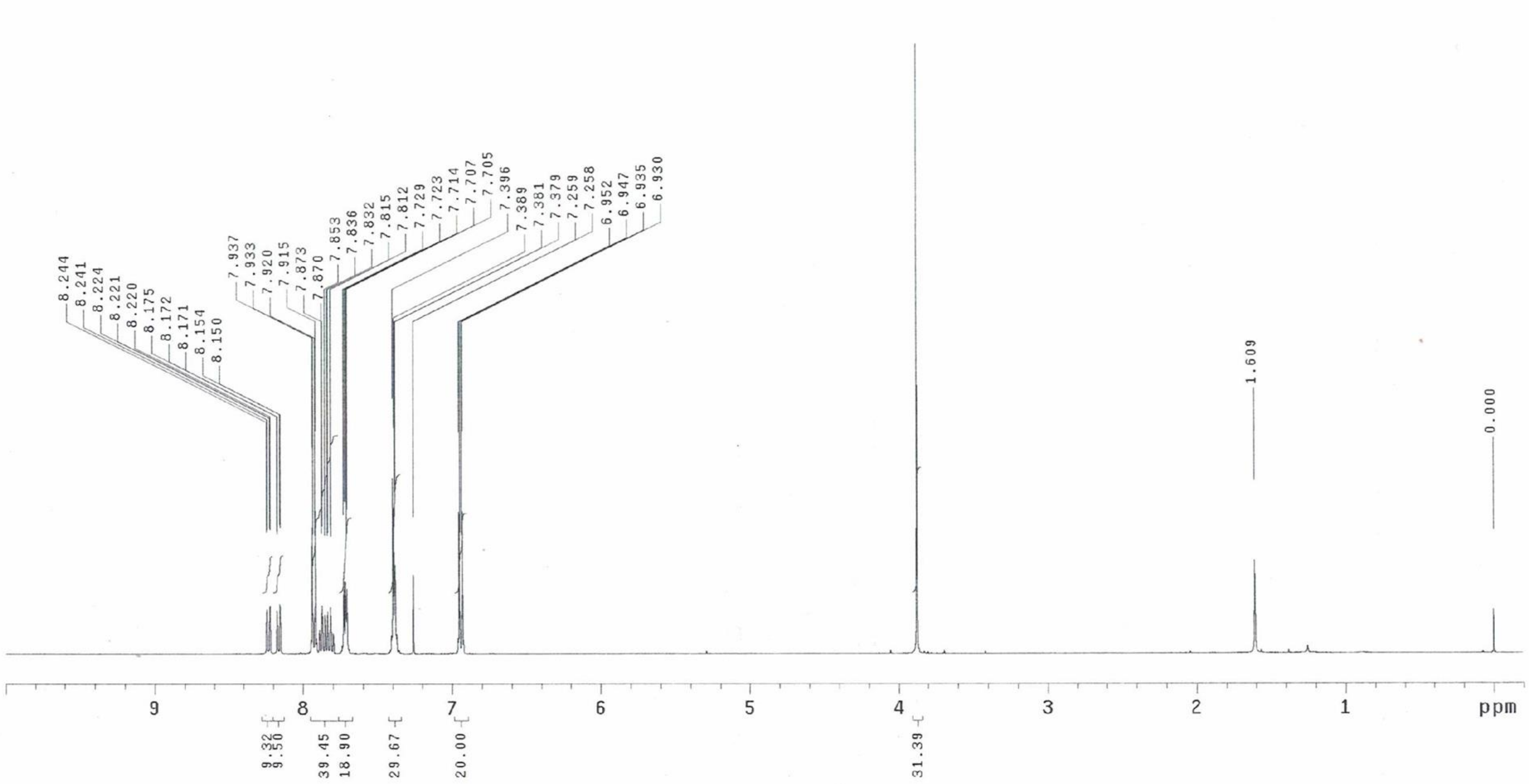

HCR-5760

Pulse Sequence: s2pul
UNITYplus-400 "unity400"
Date: Feb 20 2017
Solvent: CDCl3
Ambient temperature
Total 2416 repetitions

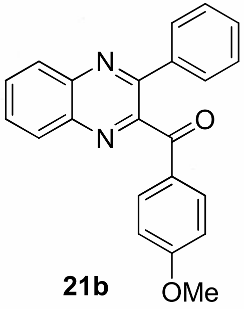

21b

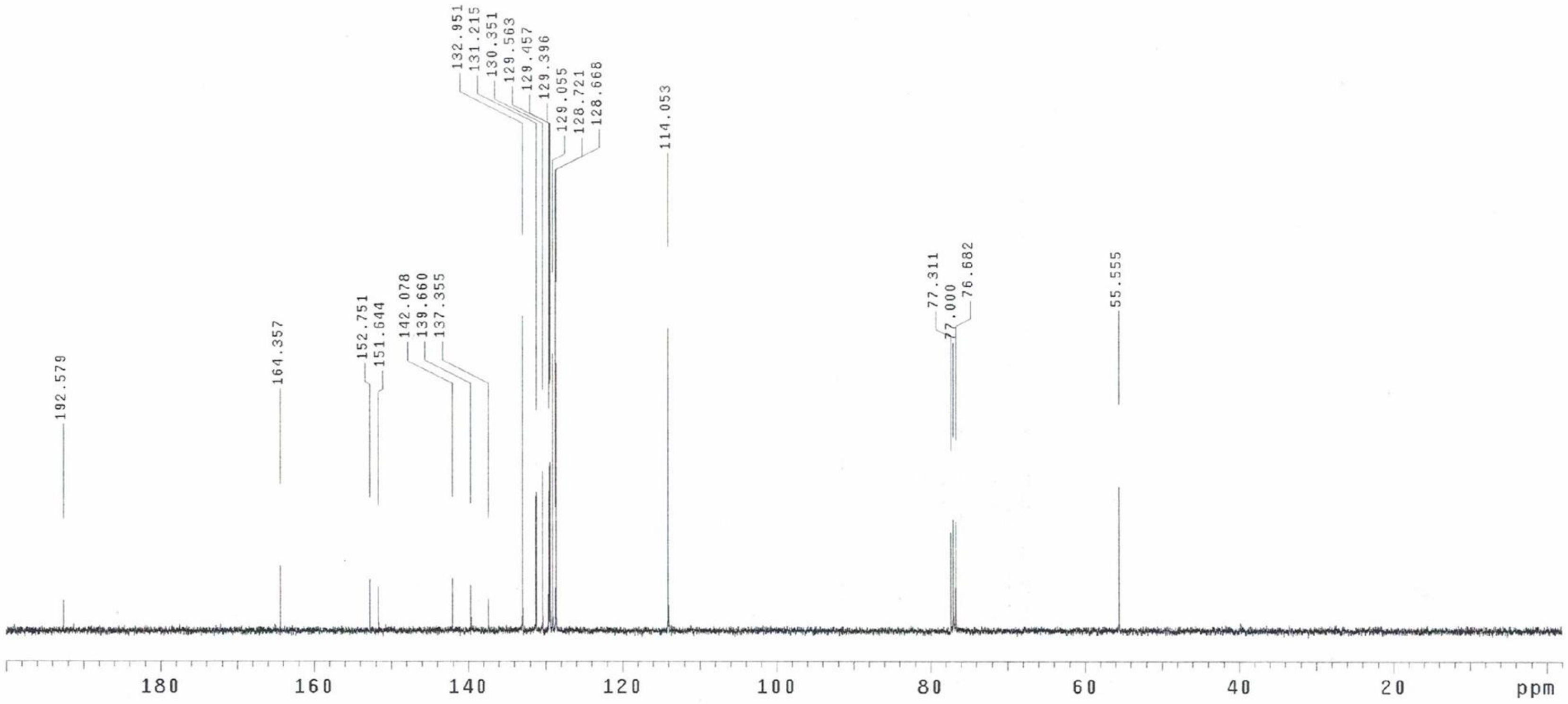

HCR-5774

Pulse Sequence: s2pul

UNITYplus-400 "unity400"
Date: Oct 20 2017
Solvent: CDCl3
Ambient temperature
Total 32 repetitions

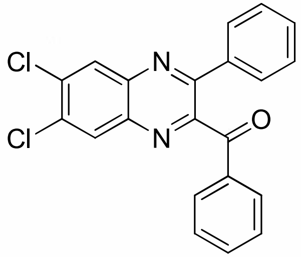

22a

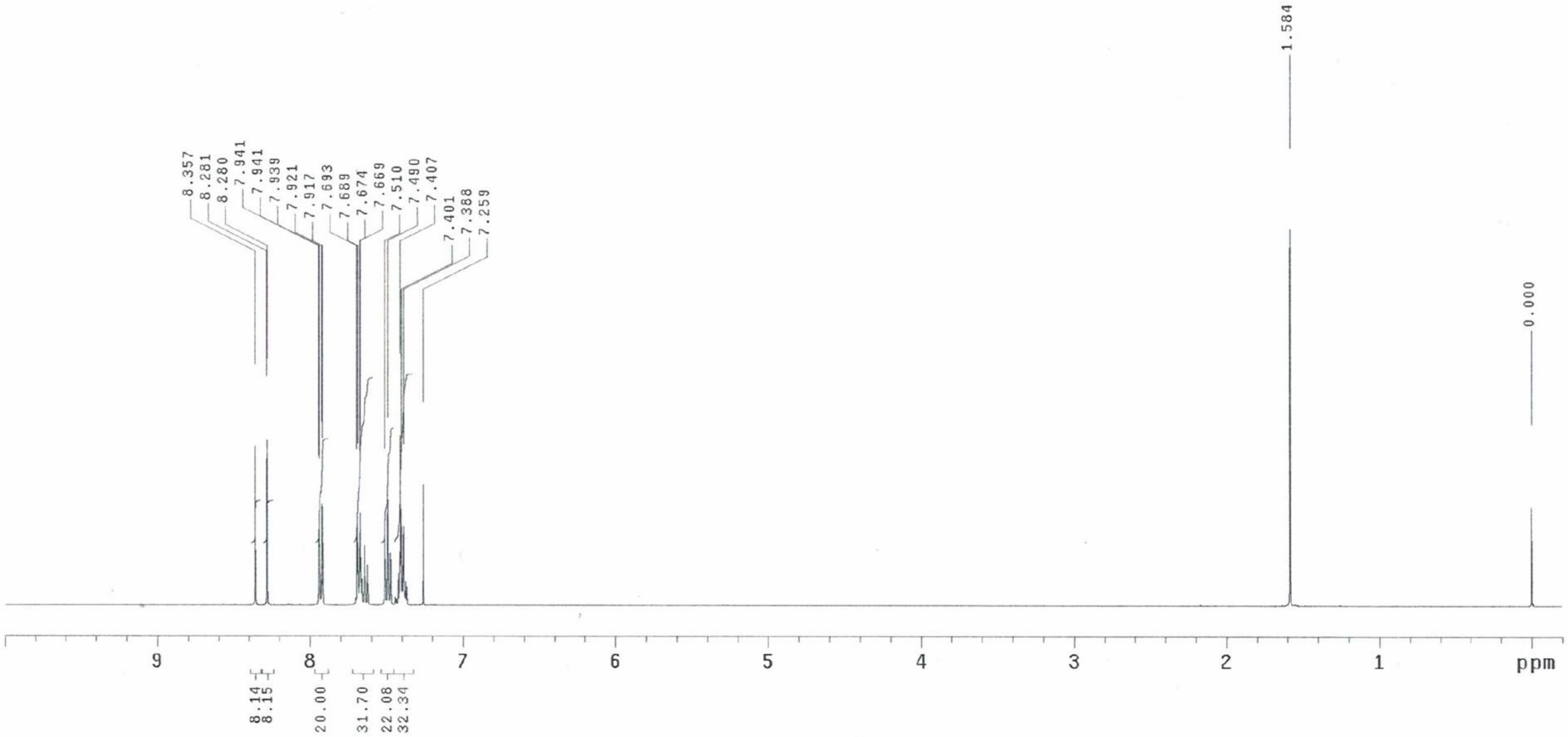

HCR-5774

Pulse Sequence: s2pul
UNITYplus-400 "unity400"
Date: Oct 20 2017
Solvent: CDCl3
Ambient temperature
Total 10208 repetitions

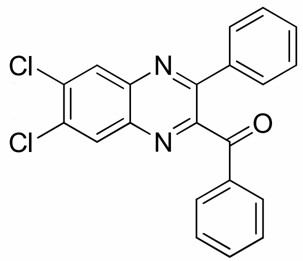

22a

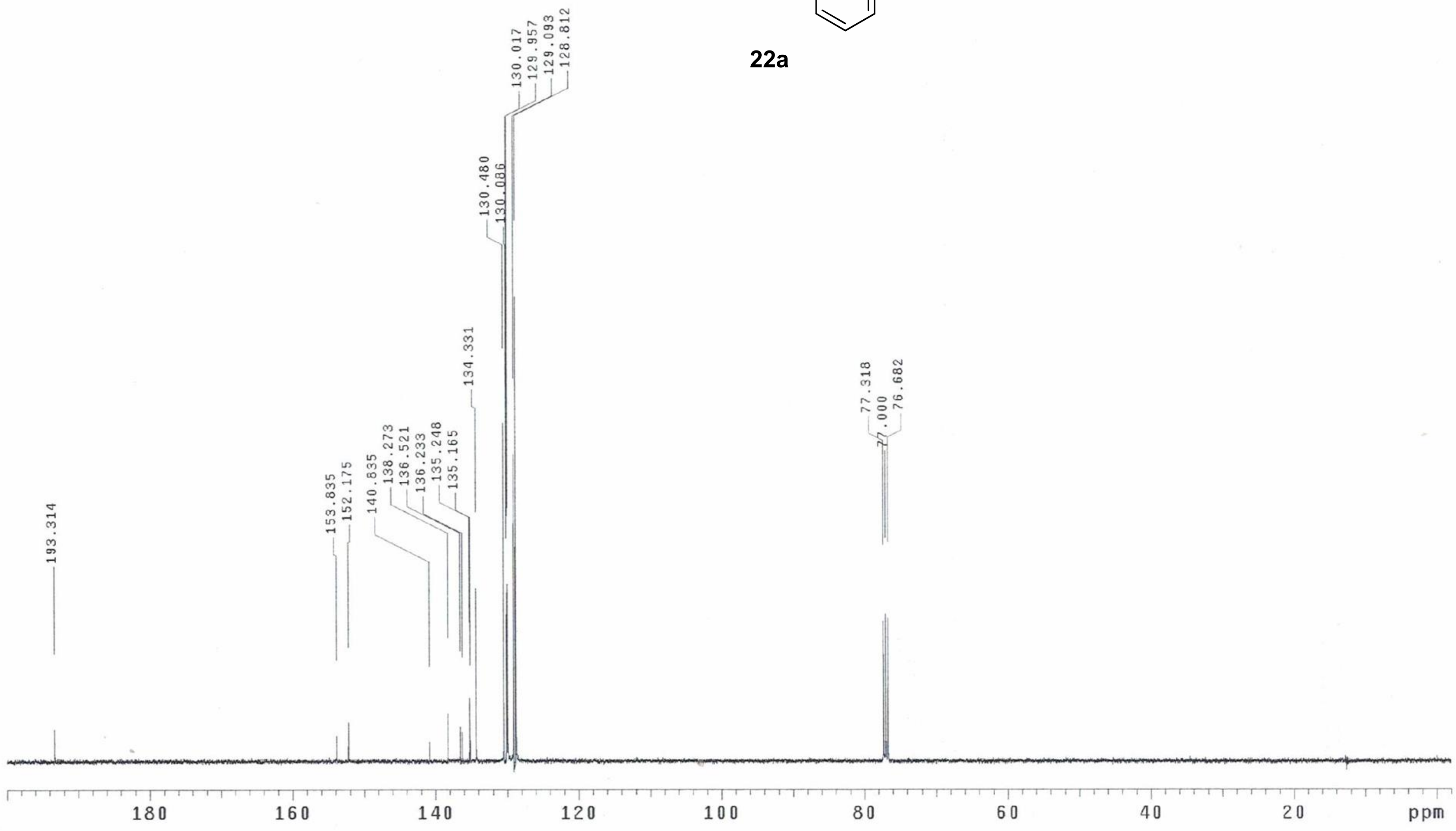

HCR-5782

Pulse Sequence: s2pul

UNITYplus-400 "unity400"
Date: Feb 23 2018
Solvent: CDCl3
Ambient temperature
Total 32 repetitions

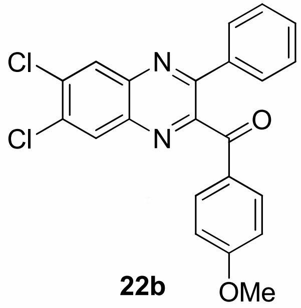

22b

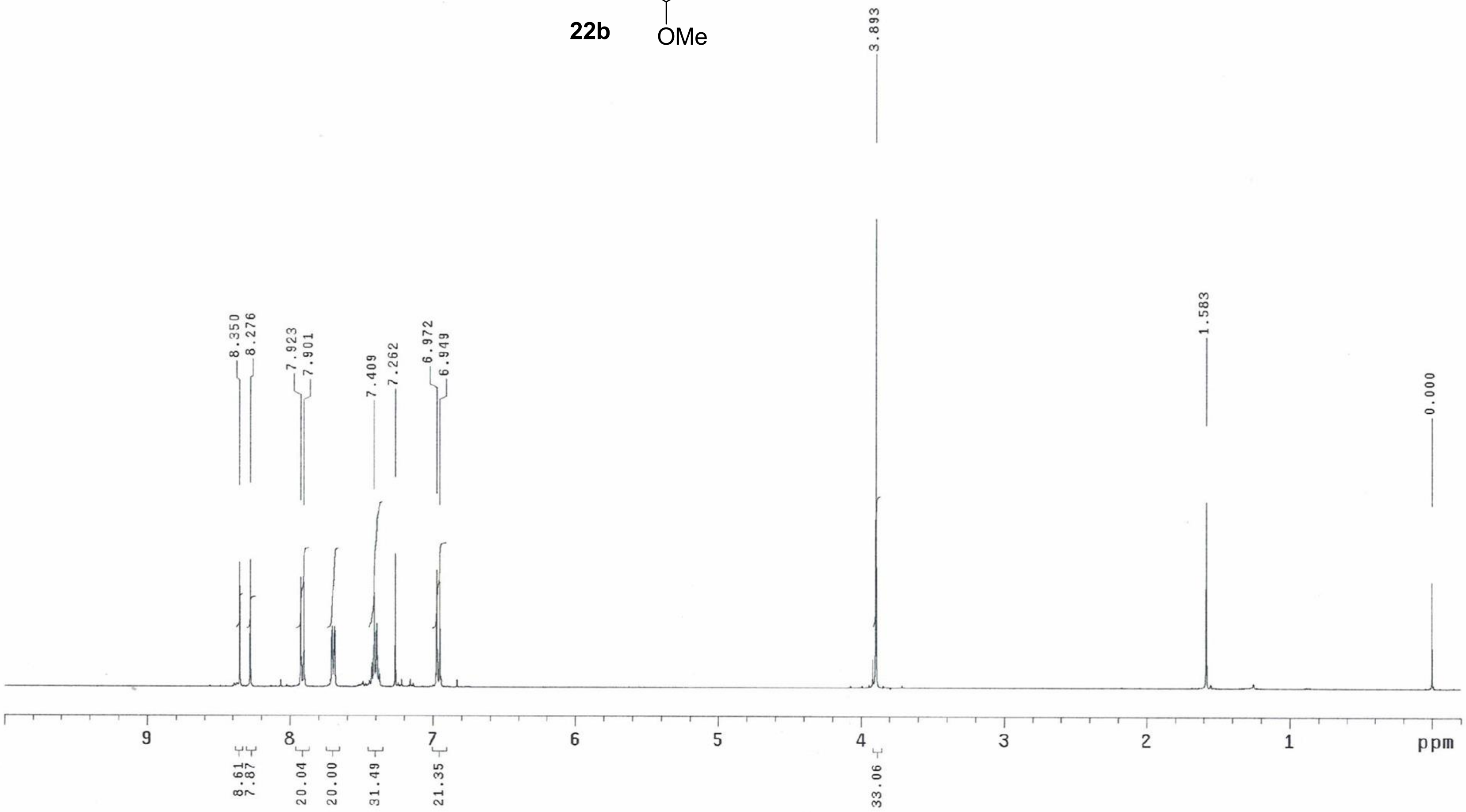

HCR-5782

Pulse Sequence: s2pul
UNITYplus-400 "unity400"
Date: Feb 23 2018
Solvent: CDCl3
Ambient temperature
Total 9056 repetitions

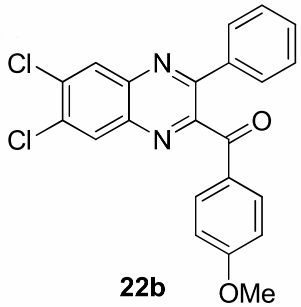

22b

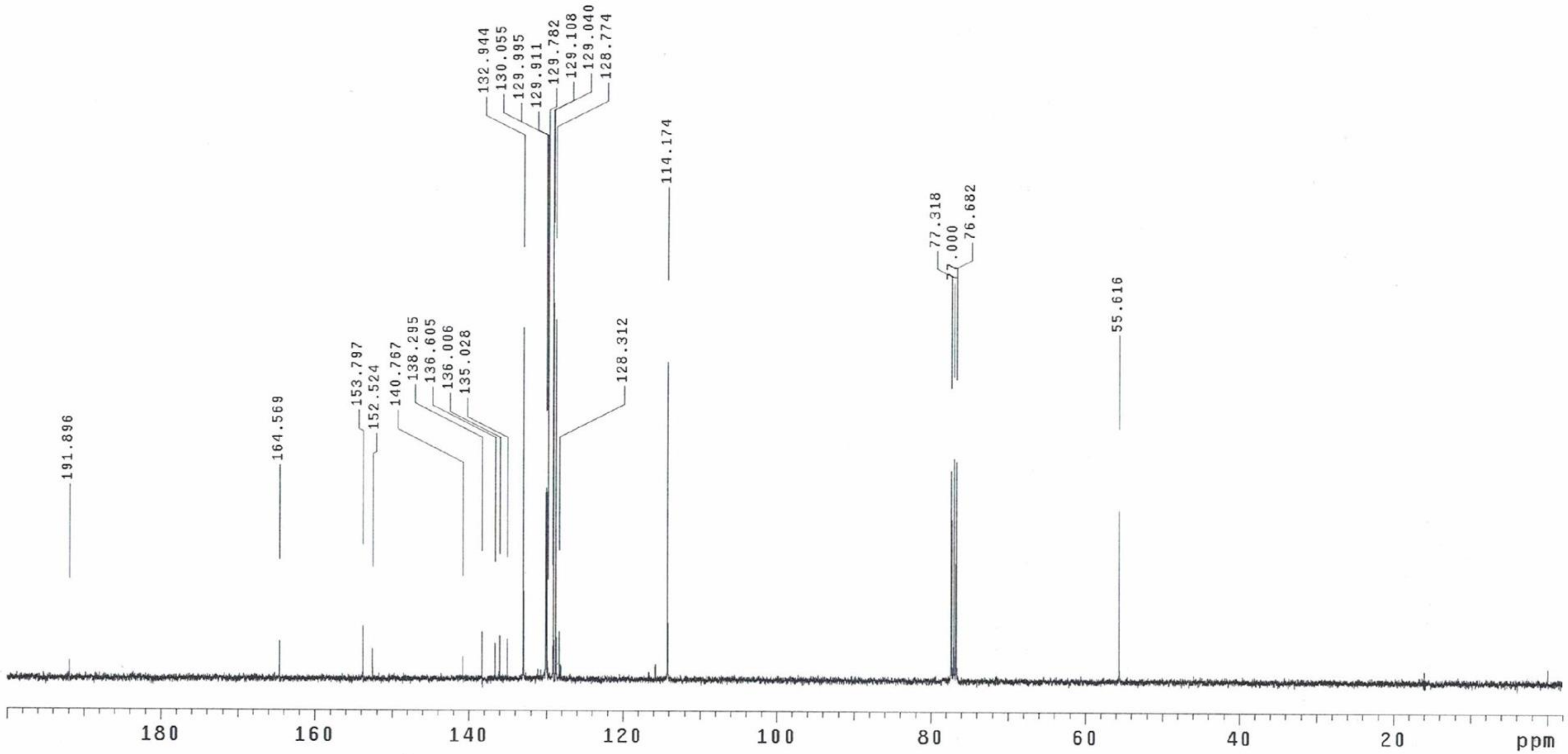

HCR-5770

Pulse Sequence: s2pul
UNITYplus-400 "unity400"
Date: Jun 8 2018
Solvent: CDCl3
Ambient temperature
Total 32 repetitions

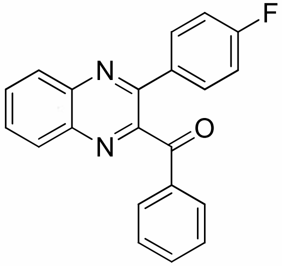

23a

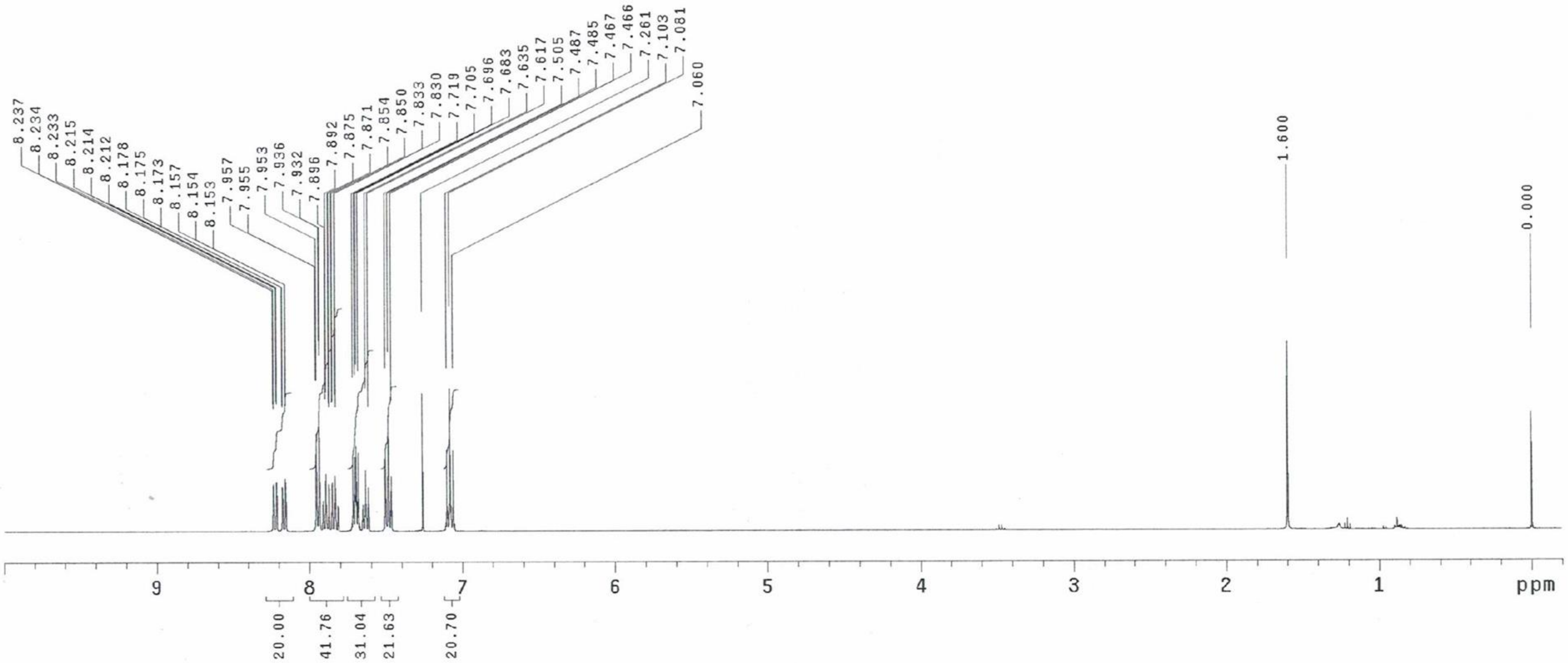

HCR-5770

Pulse Sequence: s2pul
UNITYplus-400 "unity400"
Date: Jun 8 2018
Solvent: CDCl3
Ambient temperature
Total 4192 repetitions

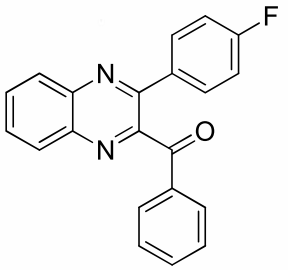

23a

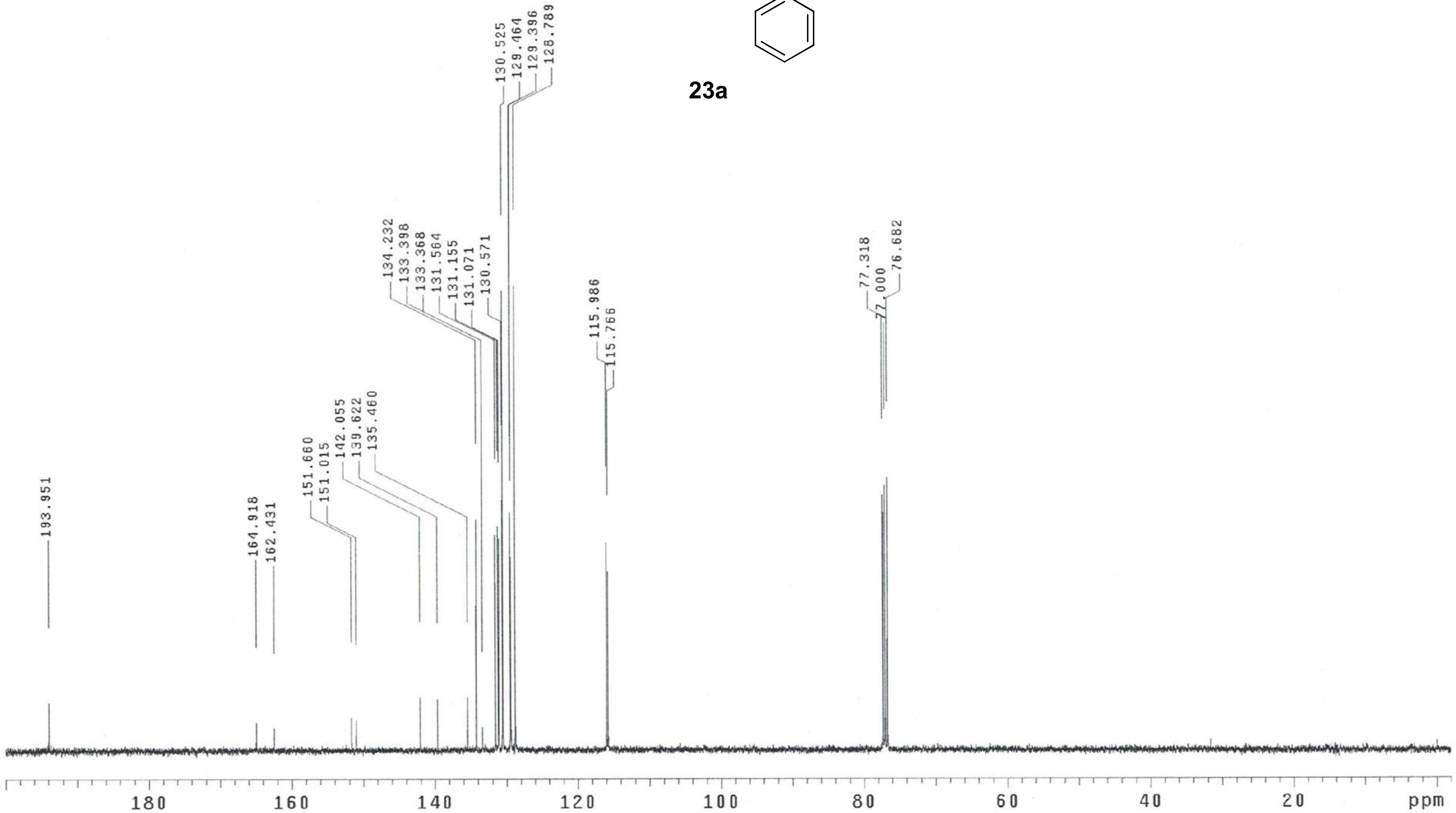

HCR-5780

Pulse Sequence: s2pul
Mercury-400BB "MerPlus400"
Date: Dec 14 2017
Solvent: cdcl3
Ambient temperature
Total 32 repetitions

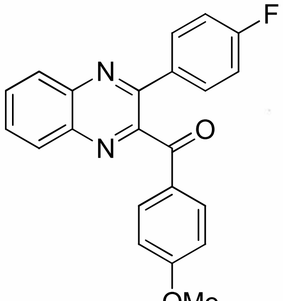

23b

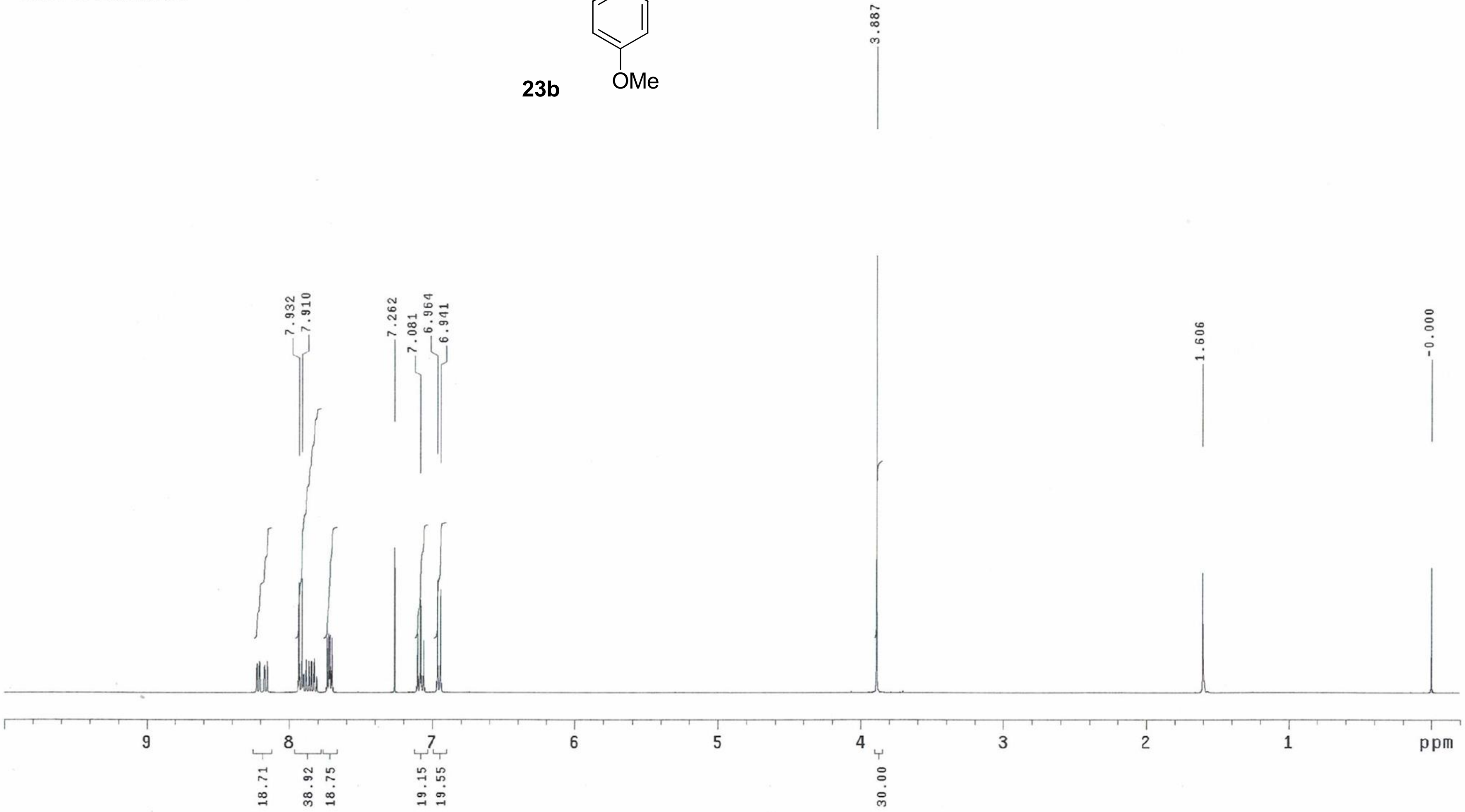

HCR-5780

Pulse Sequence: s2pul
Mercury-400BB "MerPlus400"
Date: Dec 15 2017
Solvent: cdcl3
Ambient temperature
Total 9136 repetitions

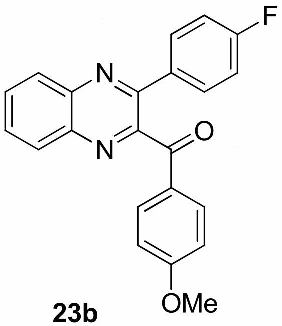

23b

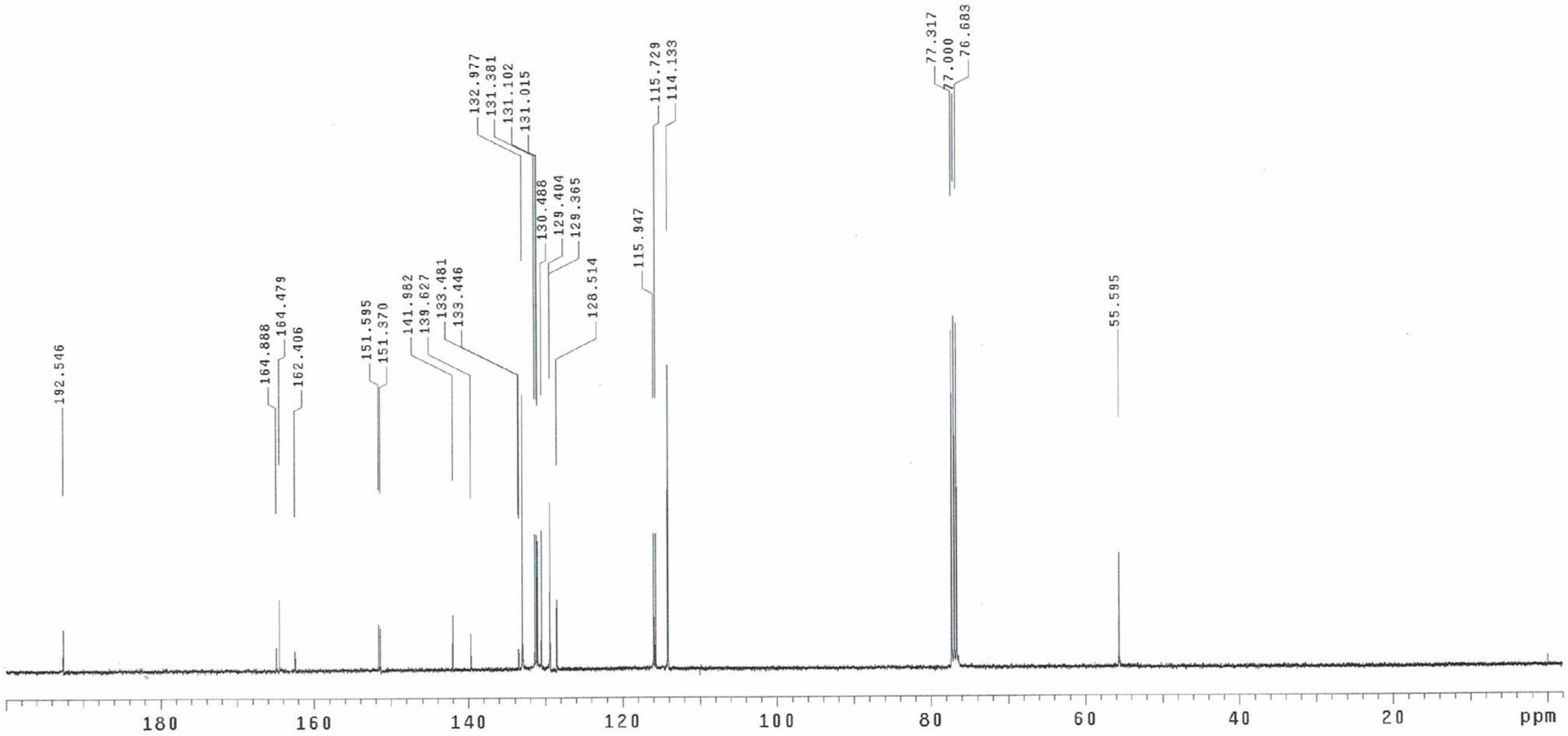

HCR-6052

Pulse Sequence: s2pul
Mercury-400BB "MerPlus400"
Date: Aug 9 2018
Solvent: cdcl3
Ambient temperature
Total 32 repetitions

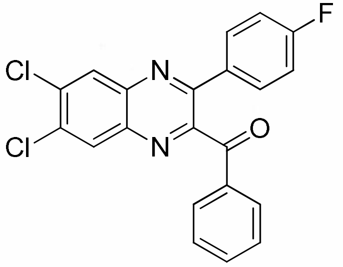

24a

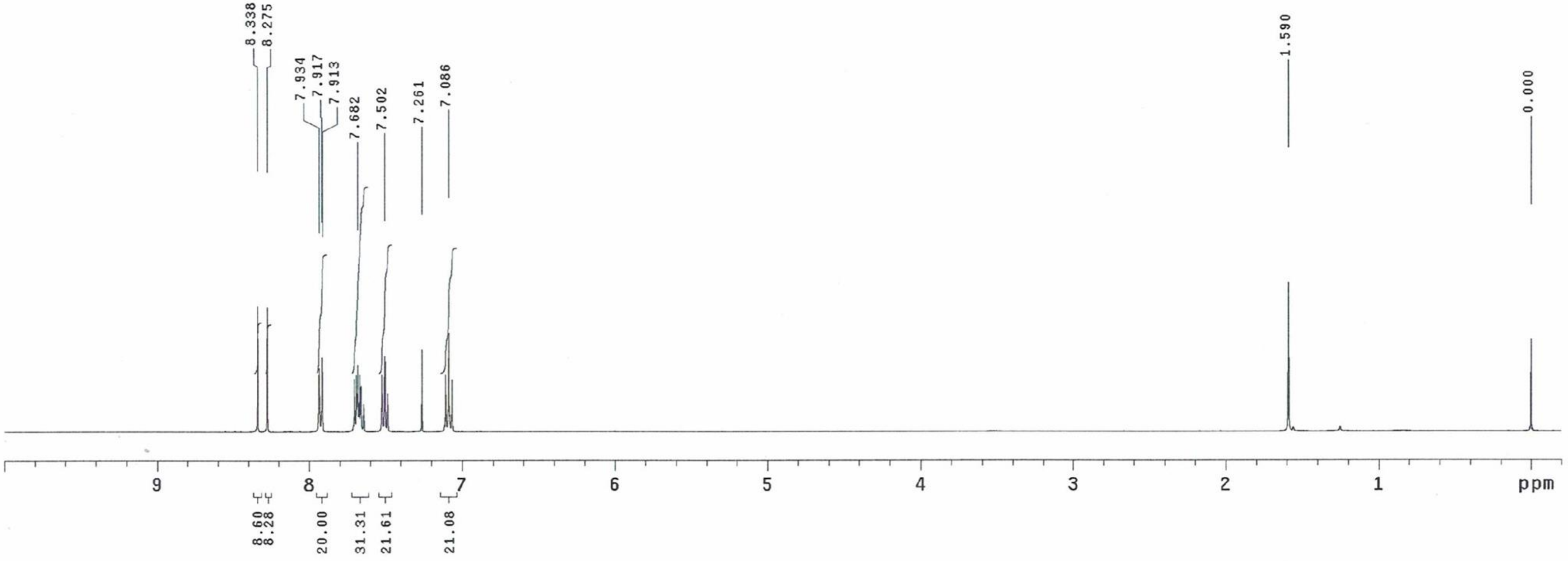

HCR-6052

Pulse Sequence: s2pul
Mercury-400BB "MerPlus400"
Date: Aug 9 2018
Solvent: cdcl3
Ambient temperature
Total 1408 repetitions

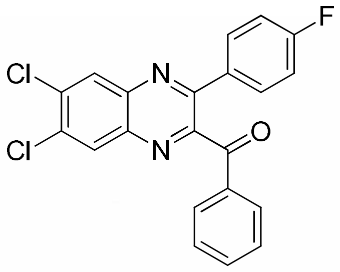

24a

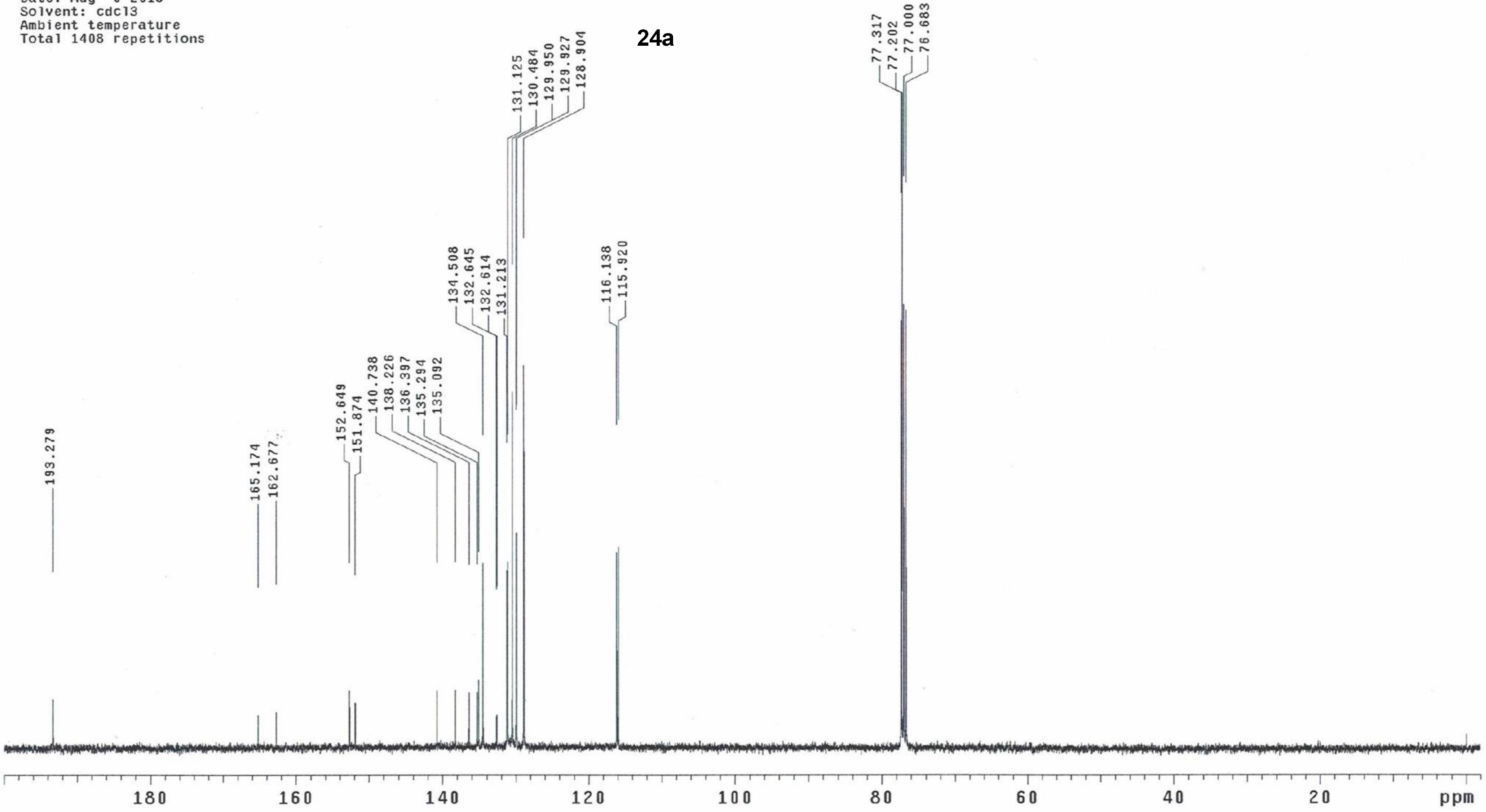

HCR-5766

Pulse Sequence: s2pul
Mercury-400BB "MerPlus400"
Date: Aug 9 2017
Solvent: cdcl3
Ambient temperature
Total 32 repetitions

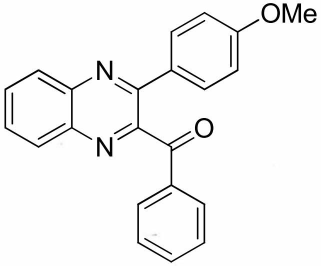

25a

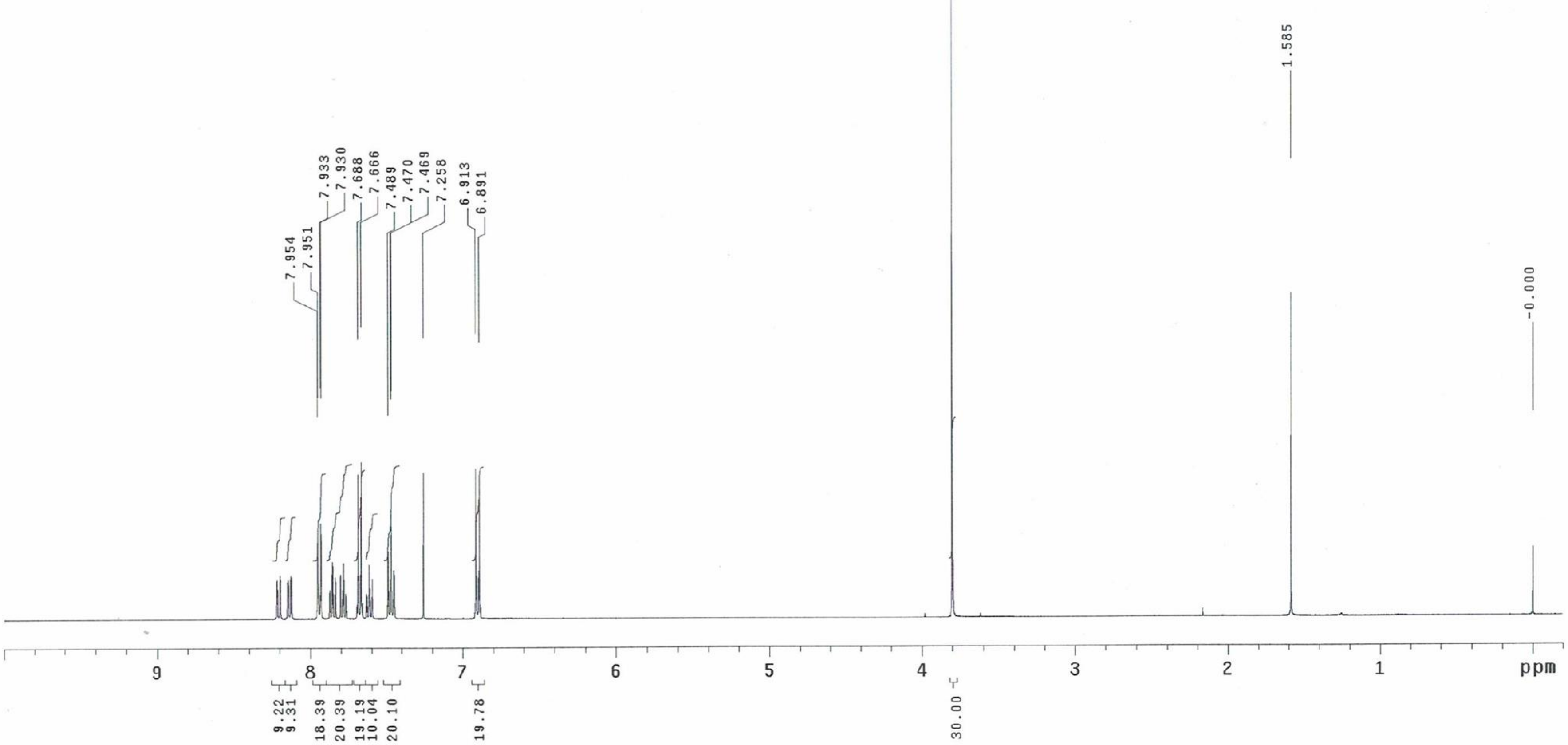

HCR-5766

Pulse Sequence: s2pul
Mercury-400BB "MerPlus400"
Date: Aug 9 2017
Solvent: cdcl3
Ambient temperature
Total 2096 repetitions

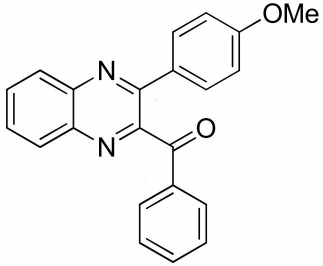

25a

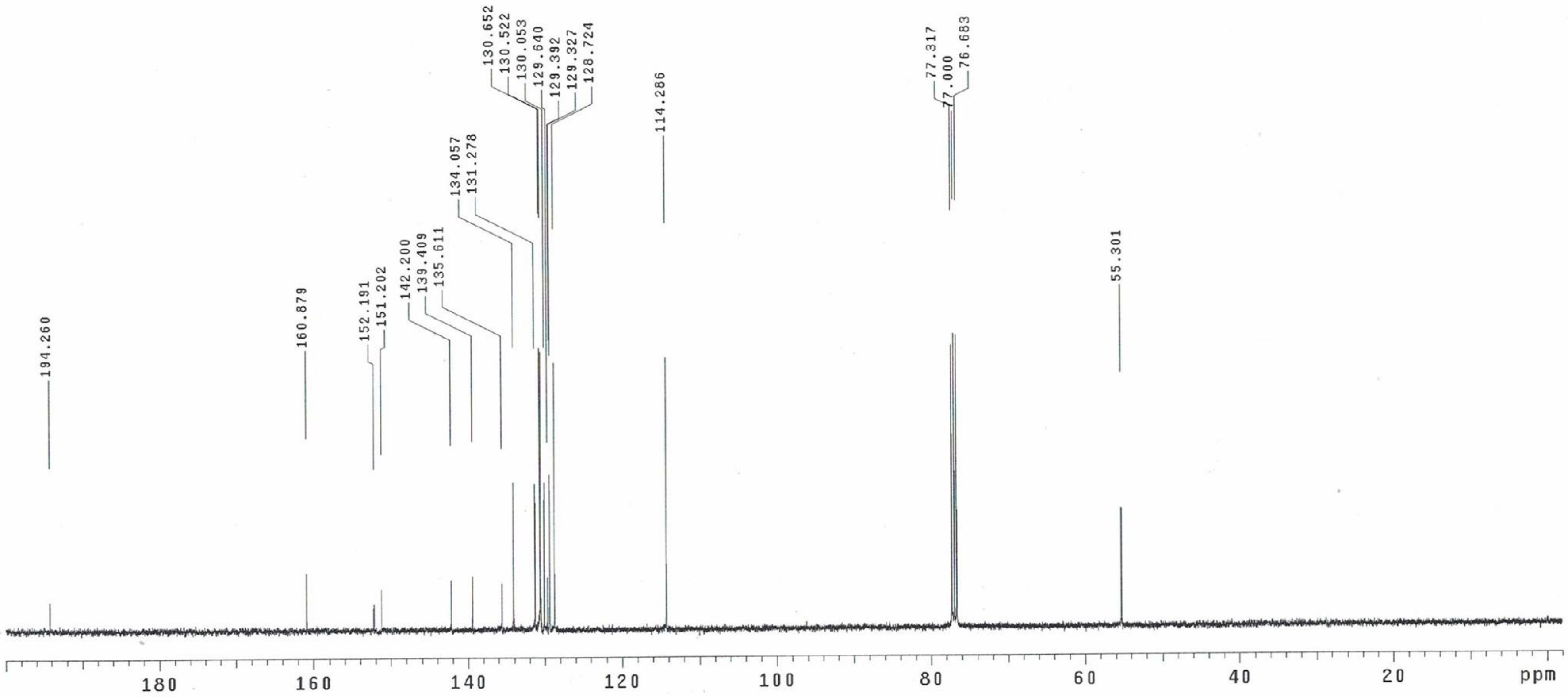

HCR-5768

Pulse Sequence: s2pul
Mercury-400BB "MerPlus400"
Date: Jun 28 2018
Solvent: cdcl3
Ambient temperature
Total 32 repetitions

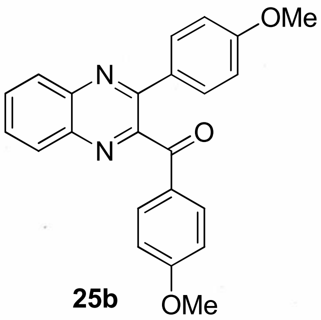

25b

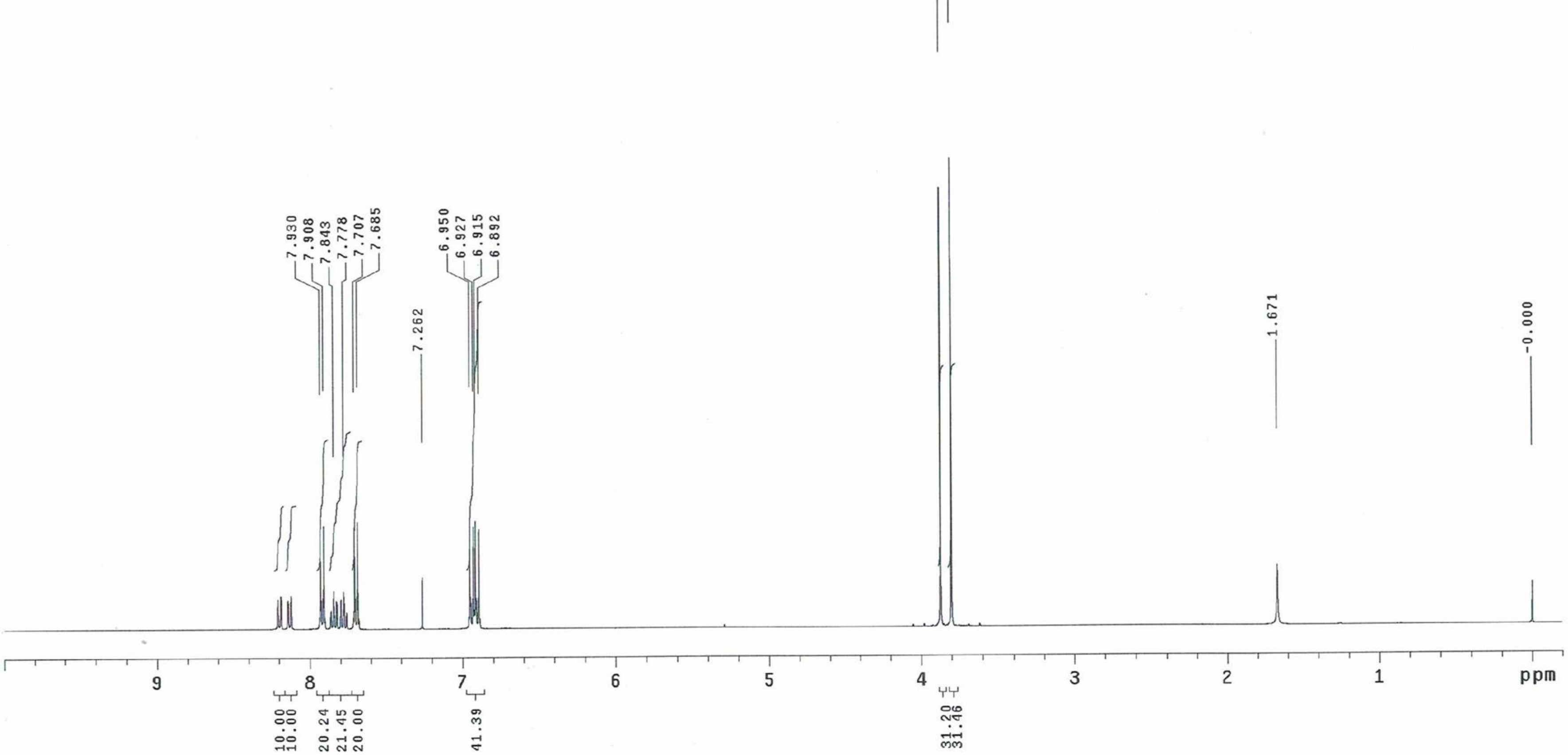

HCR-5768

Pulse Sequence: s2pul
Mercury-400BB "MerPlus400"
Date: Jun 28 2018
Solvent: cdcl3
Ambient temperature
Total 736 repetitions

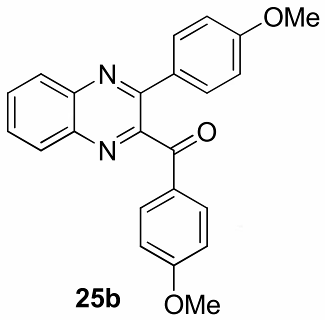

25b

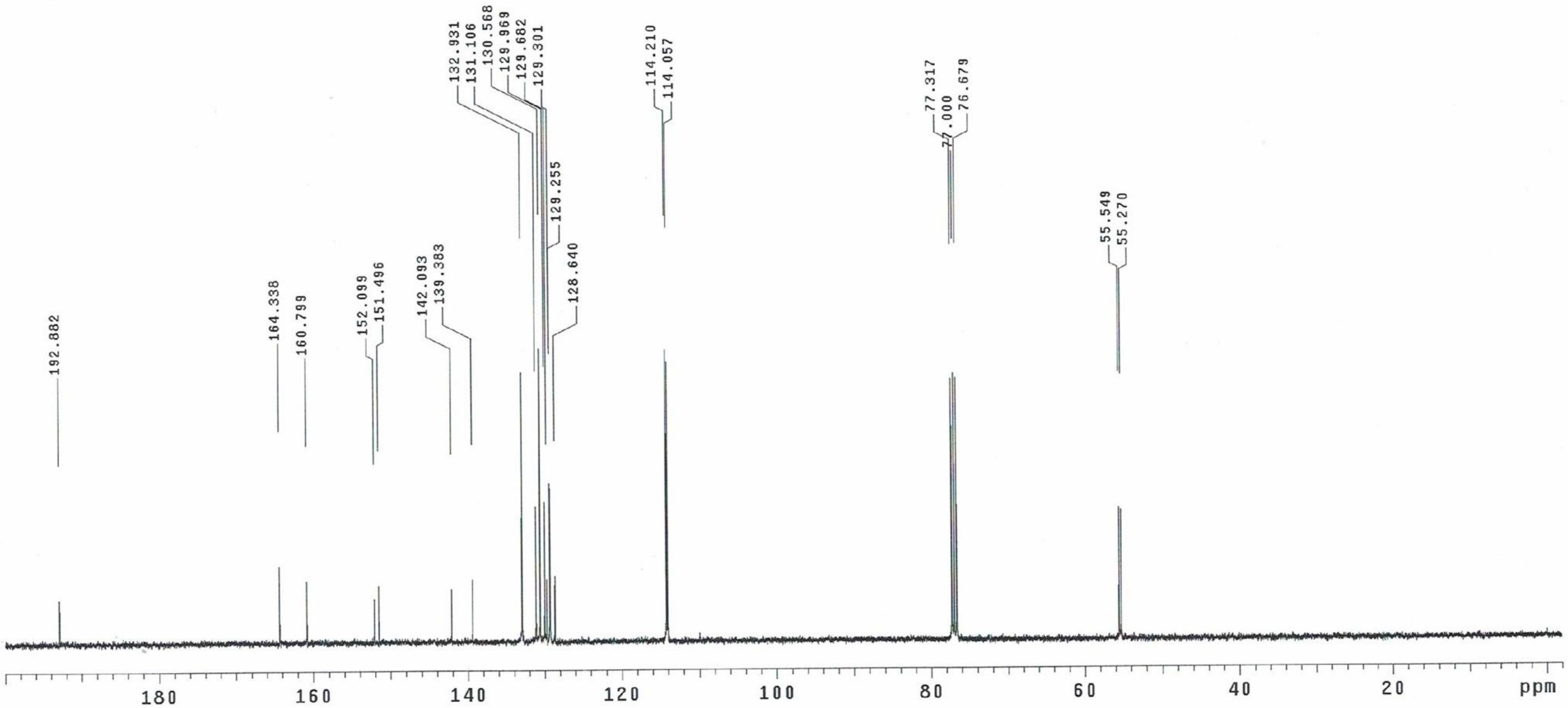

HCR-5784

Pulse Sequence: s2pul
UNITYplus-400 "unity400"
Date: Jan 30 2018
Solvent: CDCl3
Ambient temperature
Total 64 repetitions

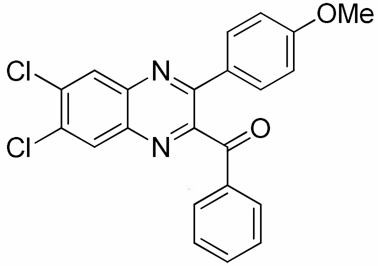

26a

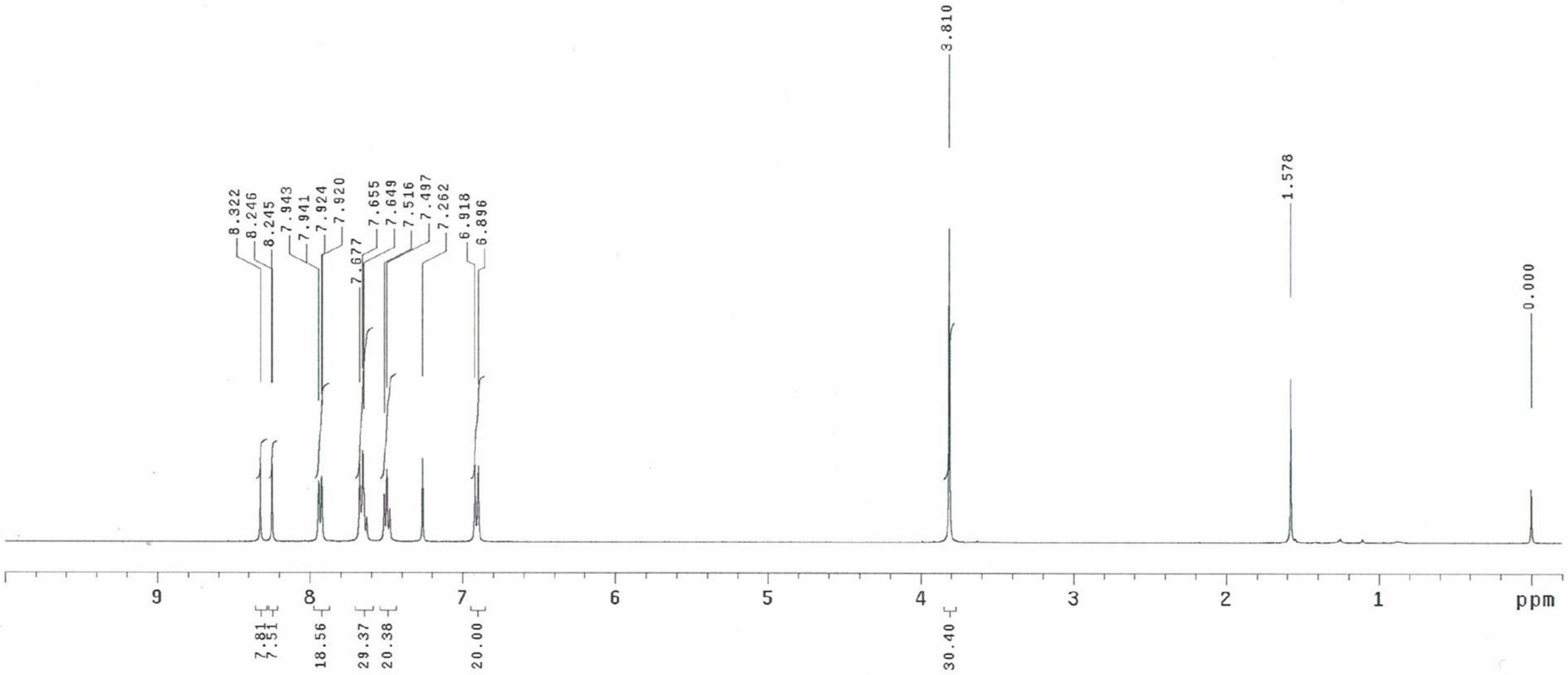

HCR-5784

Pulse Sequence: s2pul
UNITYplus-400 "unity400"
Date: Jan 30 2018
Solvent: CDCl3
Ambient temperature
Total 20480 repetitions

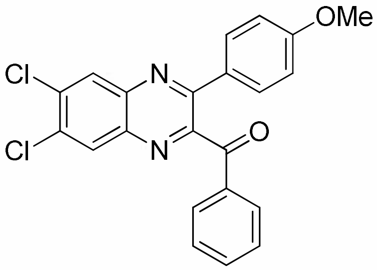

26a

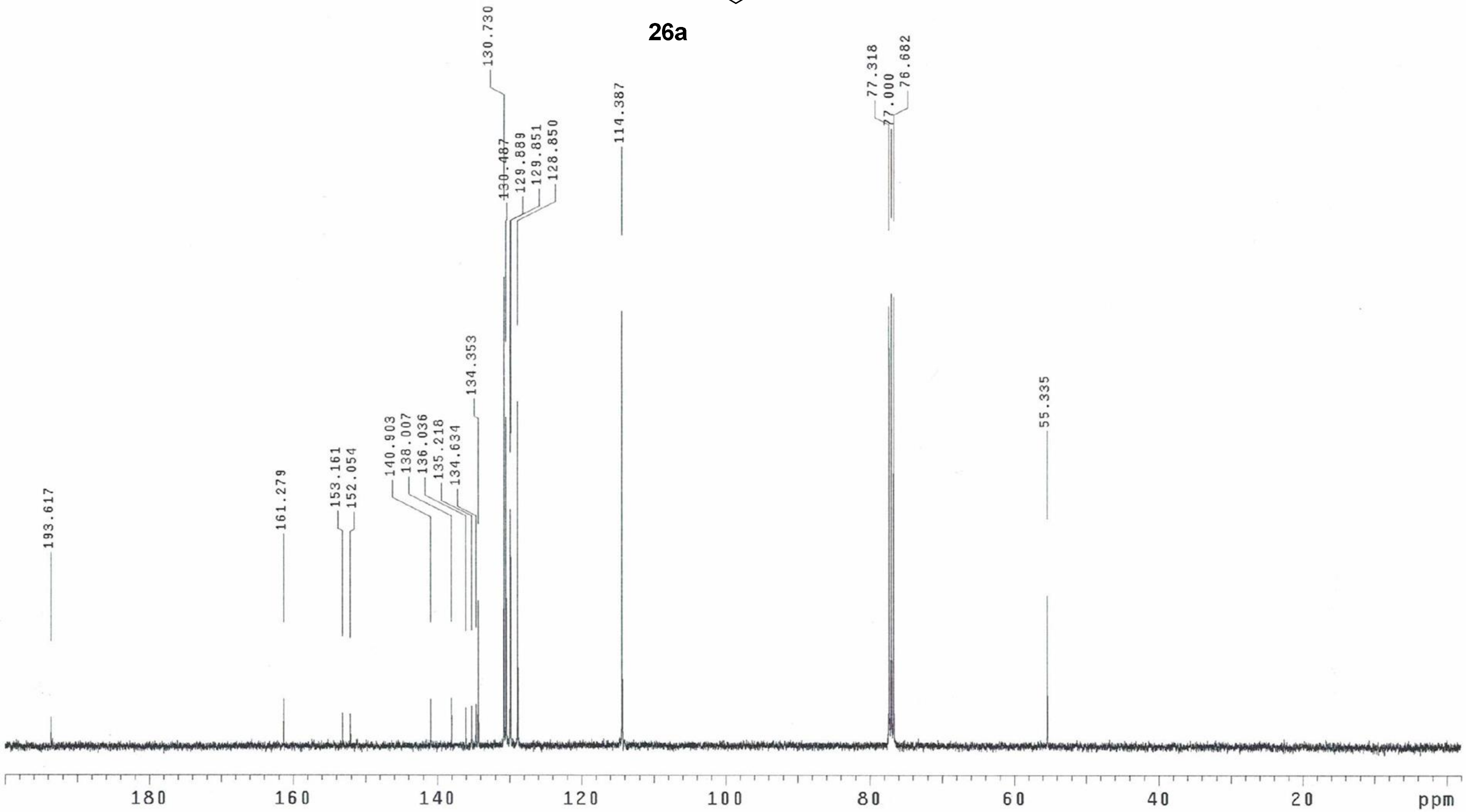

HCR-5786

Pulse Sequence: s2pul
UNITYplus-400 "unity400"
Date: Feb 23 2018
Solvent: CDCl3
Ambient temperature
Total 32 repetitions

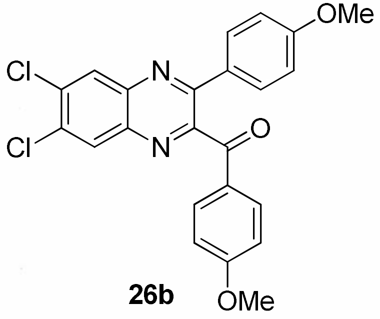

26b

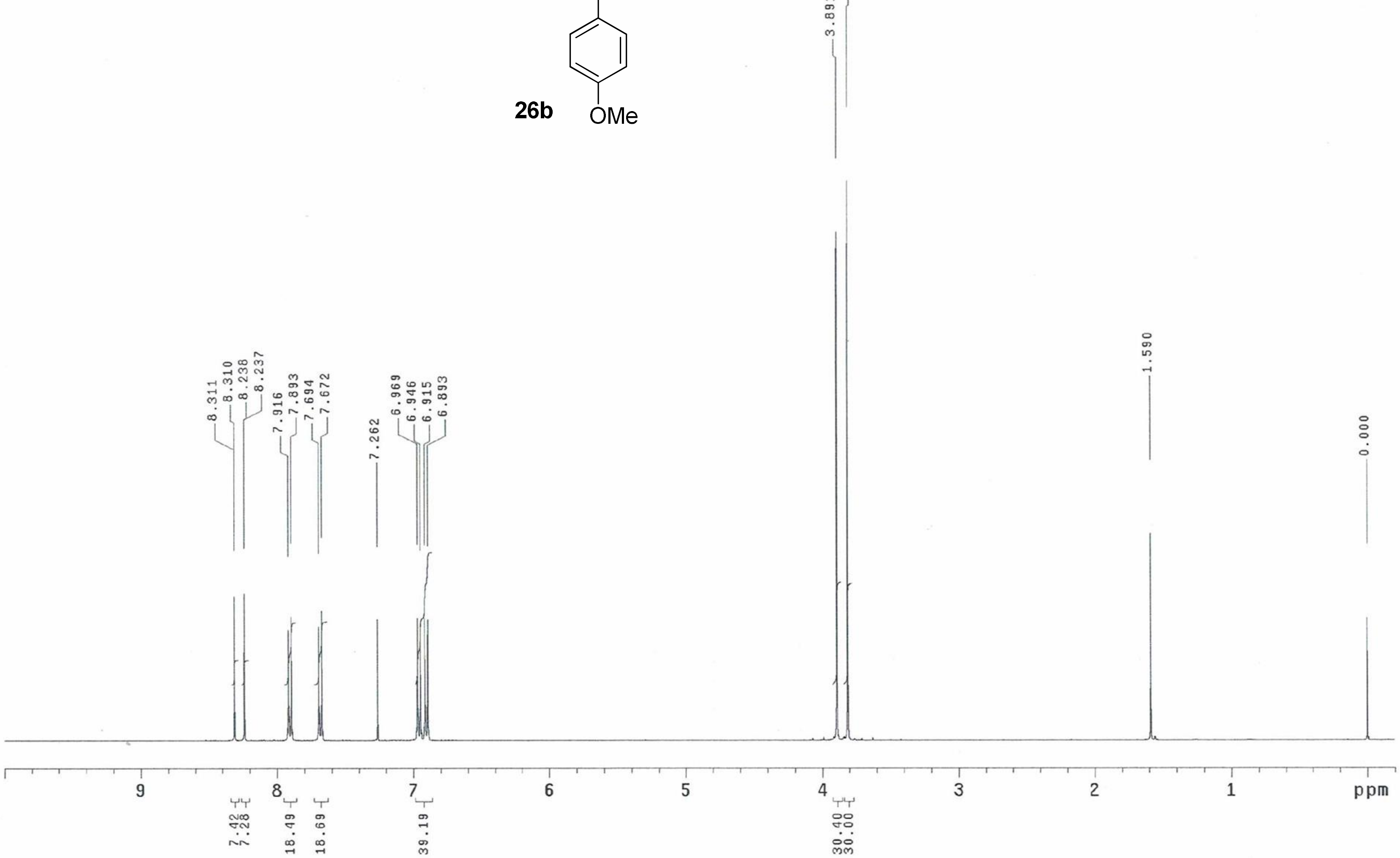

HCR-5786

Pulse Sequence: s2pul

UNITYplus-400 "unity400"
Date: Feb 23 2018
Solvent: CDCl3
Ambient temperature
Total 5760 repetitions

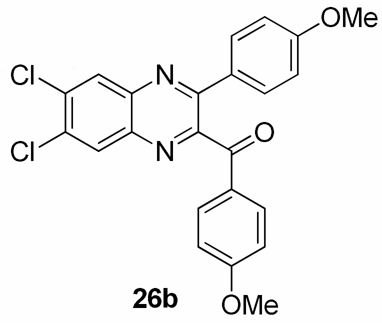

26b

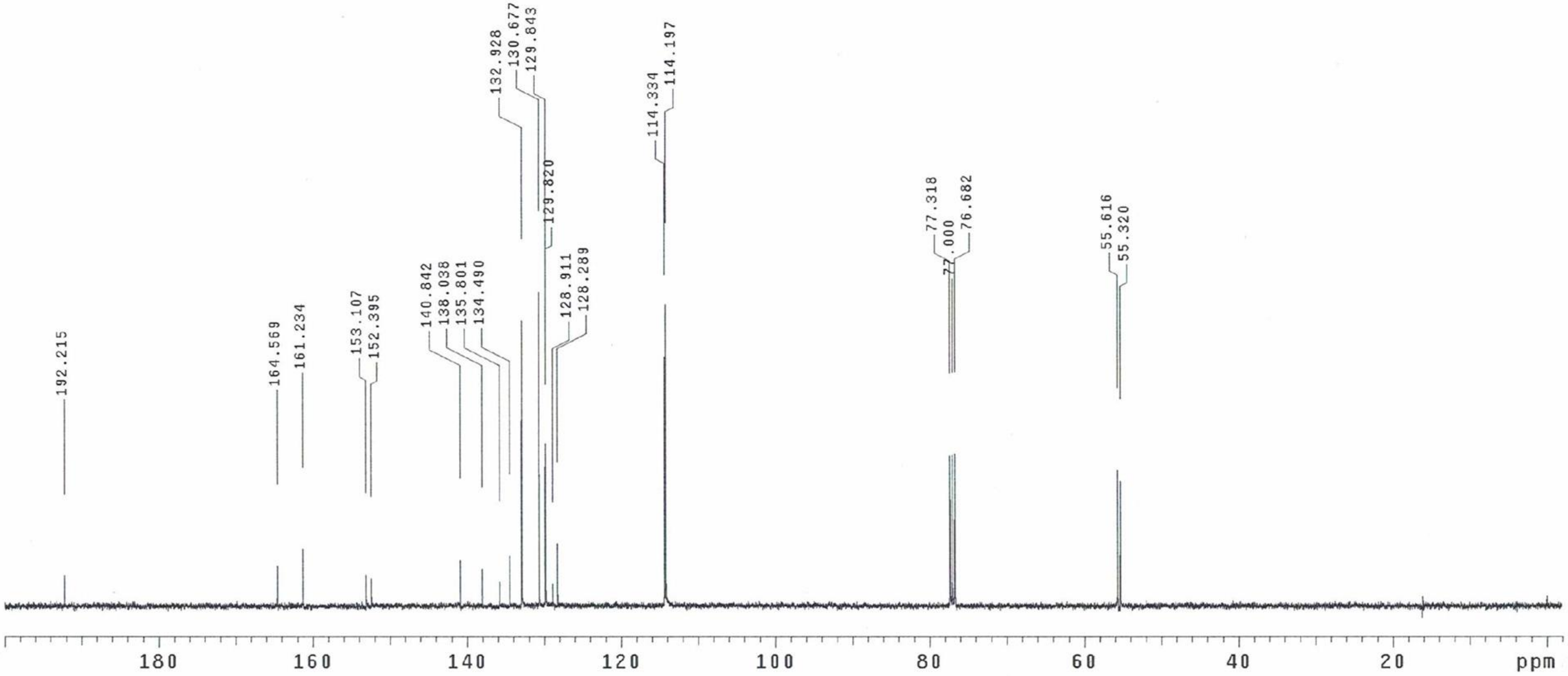

HCR-5771

Pulse Sequence: s2pul
UNITYplus-400 "unity400"
Date: Sep 20 2017
Solvent: DMSO
Ambient temperature
Total 32 repetitions

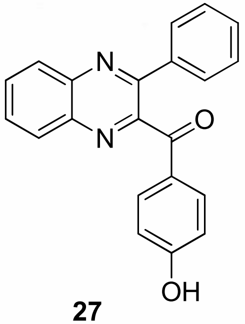

27

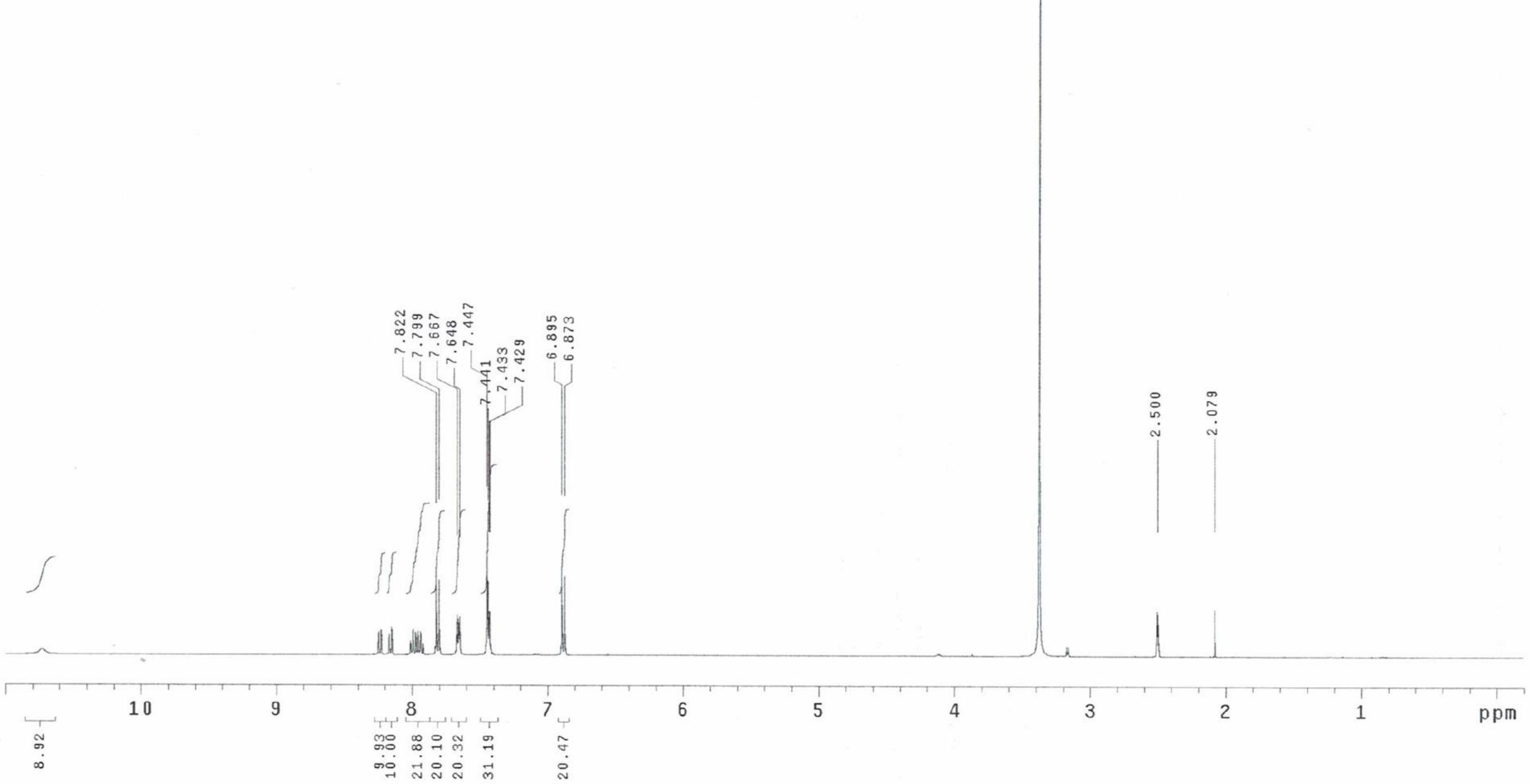

HCR-5771

Pulse Sequence: s2pul
UNITYplus-400 "unity400"
Date: Sep 20 2017
Solvent: DMSO
Ambient temperature
Total 1744 repetitions

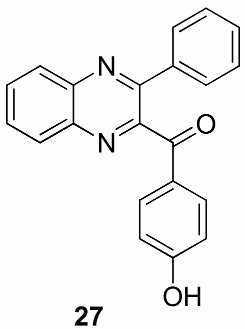

27

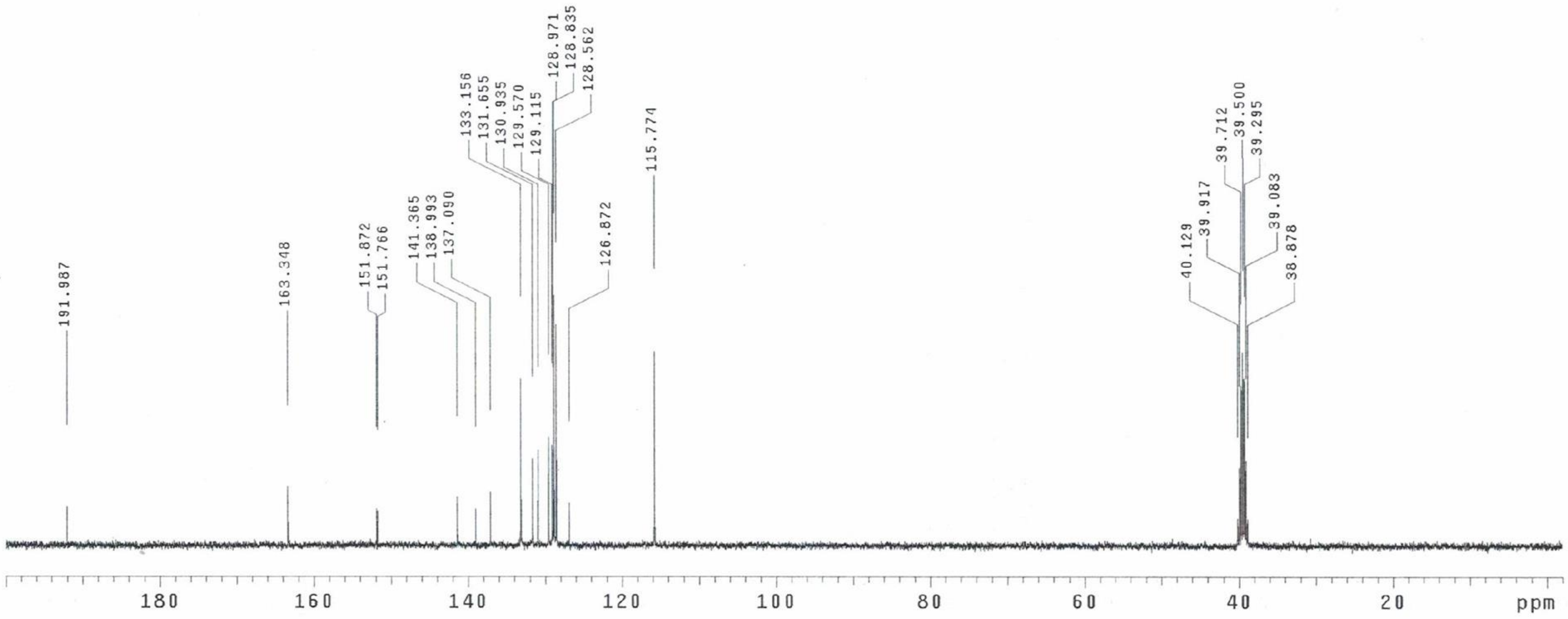

HCR-5775

Pulse Sequence: s2pul
Mercury-400BB "MerPlus400"
Date: Dec 14 2017
Solvent: dmso
Ambient temperature
Total 32 repetitions

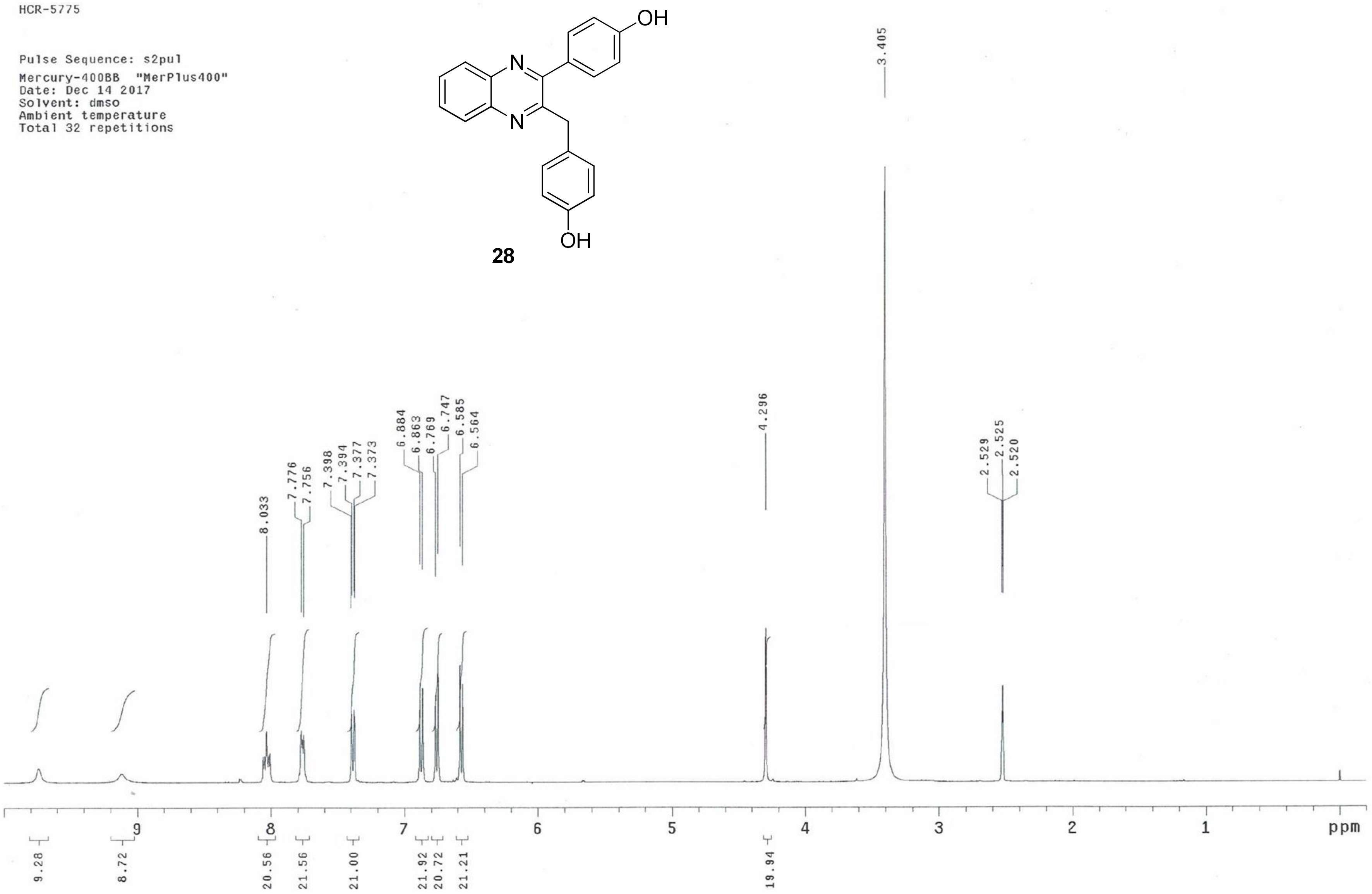

HCR-5775

Pulse Sequence: s2pul
Mercury-400BB "MerPlus400"
Date: Dec 14 2017
Solvent: dmso
Ambient temperature
Total 2048 repetitions

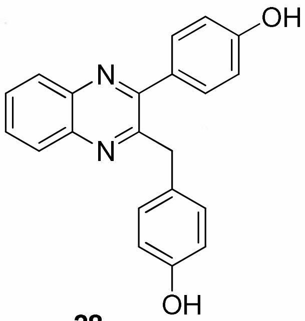

28

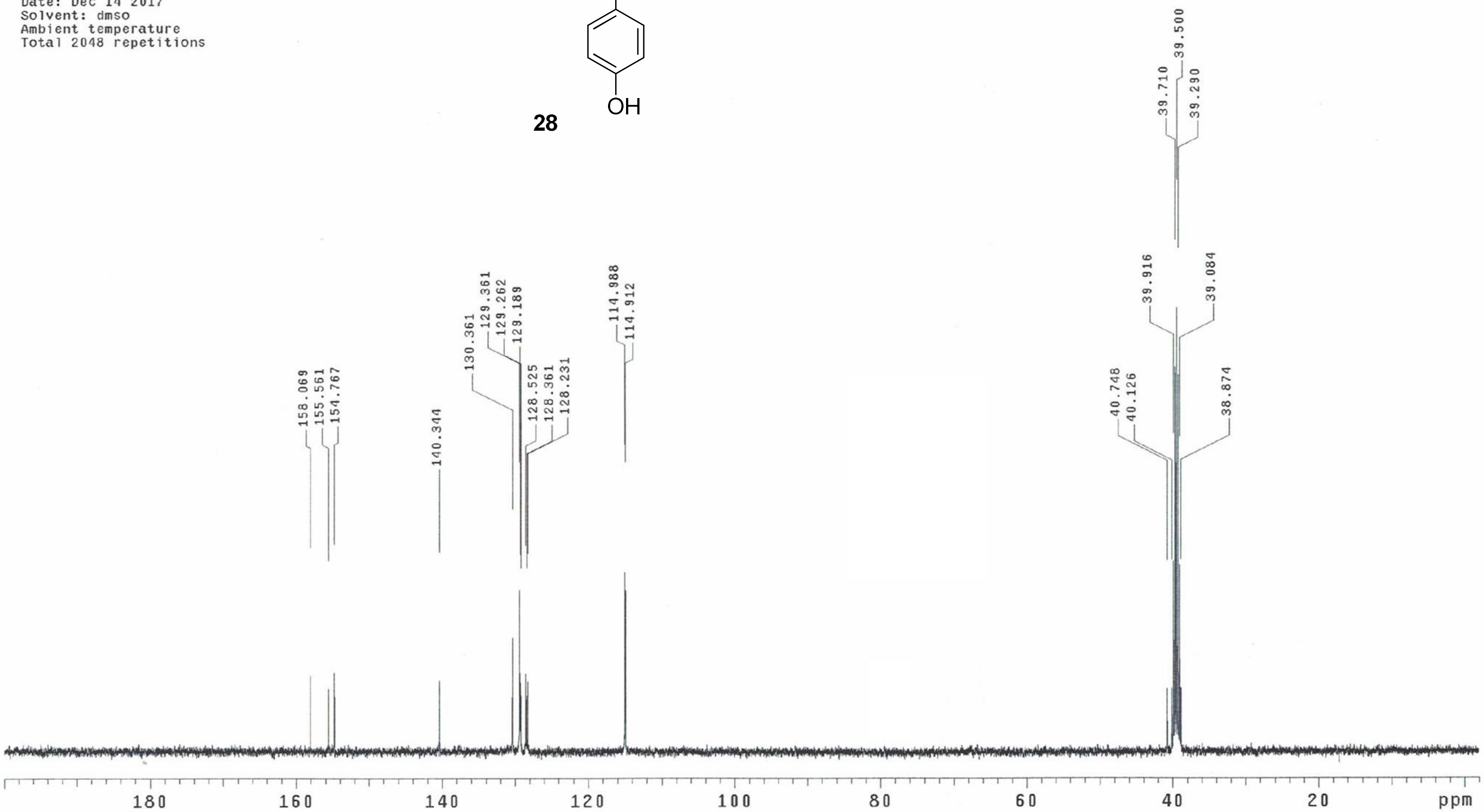

HCR-6054

Pulse Sequence: s2pul
Mercury-400BB "MerPlus400"
Date: Oct 2 2018
Solvent: dmso
Ambient temperature
Total 32 repetitions

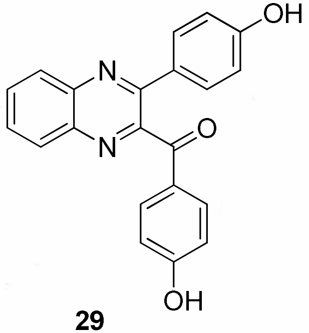

29

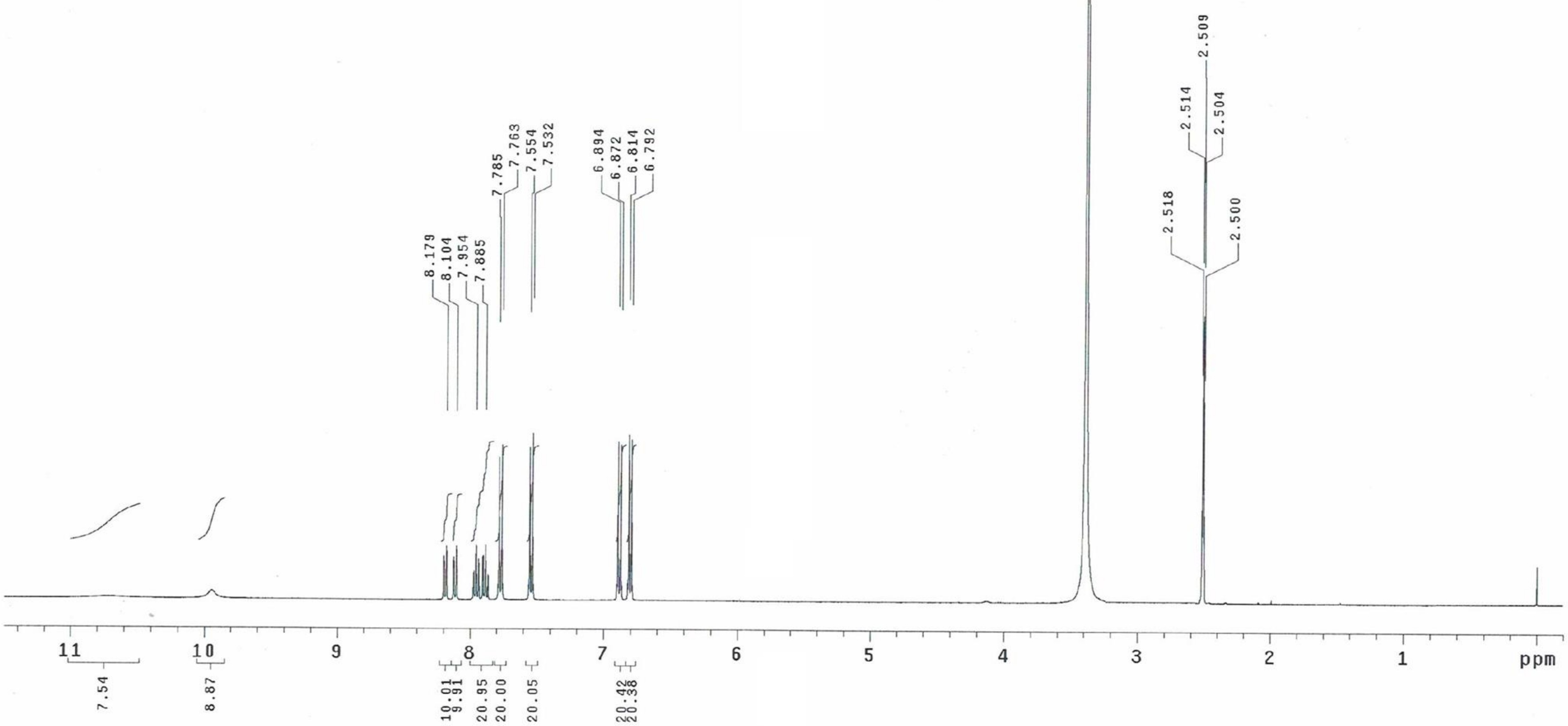

HCR-6054

Pulse Sequence: s2pul
Mercury-400BB "MerPlus400"
Date: Oct 2 2018
Solvent: dmso
Ambient temperature
Total 720 repetitions

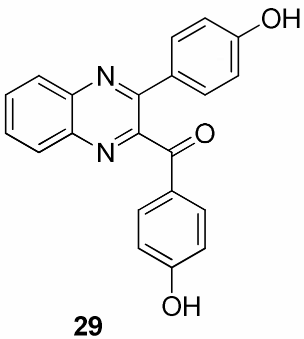

29

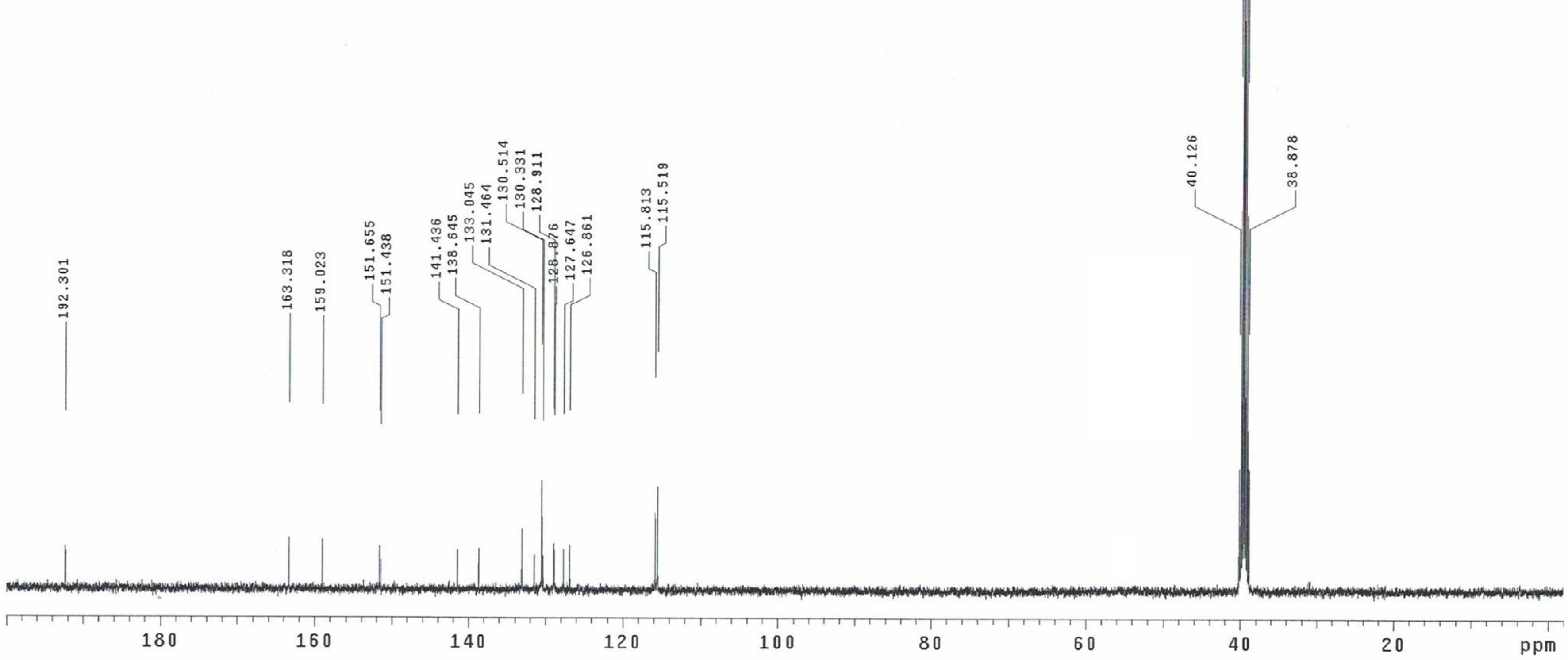

Supplement: Supplementary file 1 [file ijms-20-04786-s001.pdf]
